# Cytokinin-supplemented medium drives *de novo* shoot regeneration in petunia (*Petunia hybrida*) by bypassing the root-meristem program

**DOI:** 10.3389/fpls.2026.1918195

**Published:** 2026-08-28

**Authors:** Xiaohan Zhang, Yajie Yue, Letong Cai, Yulong Guo

**Affiliations:** 1 College of Horticulture and Landscape Architecture, Southwest University, Chongqing, China; 2 Chongqing Engineering Research Center for Floriculture, Beibei District, Chongqing, China

**Keywords:** *ARF5*, cytokinin, petunia, root meristem, shoot regeneration, *WIND1*

## Abstract

*In vitro* shoot regeneration is critical for plant propagation and serves as a model for cellular reprogramming. The classic Arabidopsis *thaliana* two-step system requires an auxin-rich callus-inducing medium to generate pluripotent callus with lateral root primordium (LRP) identity. However, whether this LRP-dependent pathway is universally required in one-step protocols using cytokinin-supplemented medium remains unclear. Here, we investigated one-step shoot regeneration from petunia (*Petunia hybrida*) leaf explants on cytokinin-supplemented medium using time-course RNA-seq. Clustering analysis of 7,357 differentially expressed genes identified six temporal expression patterns associated with wound response, metabolic activation, cell division, and shoot meristem formation. Strikingly, key LRP-associated pluripotency markers—*WUSCHEL-RELATED HOMEOBOX 5*, *PLETHORA 1a/b*, and *LATERAL ORGAN BOUNDARIES DOMAIN 16/29*—were not activated on cytokinin-supplemented medium, although they were readily induced on auxin-containing medium. These findings indicate that shoot regeneration proceeds via a root-meristem-independent pathway. Instead, on cytokinin-supplemented medium, shoot apical meristem (SAM) regulators were activated, including *ENHANCER OF SHOOT REGENERATION 1a* (*ESR1a*, day 4), *WUSCHEL* (day 4), *SHOOT MERISTEMLESS* and *NO APICAL MERISTEM* (day 7). The auxin pathway was transiently suppressed but was subsequently reconfigured, with specific upregulation of *AUXIN RESPONSE FACTOR 5* (*ARF5*), the sole induced ARF member. Overexpression of petunia *WOUND INDUCED DEDIFFERENTIATION 1a* (*WIND1a*), a key wound-induced regulator in *Arabidopsis*, did not enhance regeneration, suggesting the canonical *WIND-ESR1* axis is bypassed. We propose a model in which wounding and cytokinin coordinately drive callus proliferation, induce SAM genes, and upregulate *PhARF5* without establishing an LRP-like state. This study reveals a streamlined, root-meristem-independent route to shoot regeneration, underscoring the plasticity of plant regenerative programs.

## Introduction

In plant tissue culture, cytokinins and auxins function synergistically as primary regulators of *in vitro* organogenesis, with their relative ratio determining developmental fate: a low auxin-to-cytokinin ratio promotes shoot induction, whereas a high ratio favors root formation; an intermediate ratio typically triggers callus proliferation (Skoog and Miller, 1957; Raspor et al., 2021; Šmeringai et al., 2023). However, due to substantial inter- and intraspecific variation in explant responsiveness, no universal protocol exists for efficient regeneration. Consequently, optimization of phytohormone type and concentration must be empirically tailored to the genotype and explant source, resulting in a wide spectrum of species- and explant-specific regeneration workflows.

A typical protocol for *de novo* shoot organogenesis (DNSO) from *Arabidopsis thaliana* root explants consists of two sequential steps: explants are first cultured on auxin-rich callus-inducing medium (CIM) for 3–5 d and then transferred to cytokinin-rich shoot-inducing medium (SIM) to promote organ formation (Valvekens et al., 1988; Motte et al., 2014; Maqbool et al., 2025). During incubation on CIM, auxin promotes the degradation of INDOLE-3-ACETIC ACID INDUCIBLE 14 (IAA14), releasing AUXIN RESPONSE FACTOR 7 (ARF7) and ARF19 from repression and allowing them to directly induce the expression of *LATERAL ORGAN BOUNDARIES DOMAIN* (*LBD*) genes (Okushima et al., 2007; Fan et al., 2012). LBD protein complexes (e.g., with bZIP59) activate the cell cycle regulator E2Fa to drive cellular reprogramming (Berckmans et al., 2011) and upregulate cell wall modifiers (e.g., *FAD-BINDING BERBERINE*, *PECTIN METHYLESTERASE 2*, *EXPANSIN14*) to remodel cell walls (Lee et al., 2013; Xu et al., 2018b, Xu et al., 2018a). These actions collectively enable cells to re-enter the cell cycle and proliferate.

In parallel, ARF7/19 regulate the expression of key root meristem genes through two distinct transcriptional modules. First, they promote the transcription of *WRKY23*, which directly binds to and activates the promoters of *PLETHORA 3* (*PLT3*) and *PLT7* (Xu et al., 2023). The PLT3 and PLT7 proteins in turn induce the expression of their downstream targets, *PLT1*, *PLT2*, and *WUSCHEL-RELATED HOMEOBOX* 5 (*WOX5*). Second, ARF7/19-activated LBD proteins induce the degradation of the transcriptional repressor BASIC HELIX-LOOP-HELIX 041 (bHLH041), thereby relieving its repression on *PLT1*, *PLT2*, and *WOX5* (Xu et al., 2023). These two pathways act synergistically to establish a gene expression network resembling that of the lateral root primordium (LRP). Auxin also upregulates other LRP-associated genes, including direct targets such as *WOX11* (Hu and Xu, 2016; Liu et al., 2018; Wan et al., 2023). Additionally, genes such as *SCARECROW* (*SCR*) and *SHORT-ROOT* (*SHR*) are induced, although the precise regulatory link to auxin signaling remains less clear (Sugimoto et al., 2010; Bustillo-Avendaño et al., 2018; Kim et al., 2018; Zhou et al., 2019). Their induction is consistent with their established roles in patterning root-like structures.

Notably, irrespective of whether the explant originates from root or aerial tissues, the resulting callus originates from pericycle or pericycle-like cells and exhibits a transcriptional and developmental profile similar to that of LRP, as evidenced by the expression of canonical LRP marker genes (Atta et al., 2009; Sugimoto et al., 2010; Ikeuchi et al., 2016; Chen et al., 2024). Moreover, single-cell transcriptome analyses have demonstrated that LRP-like cells within the callus play a crucial role in shoot meristem formation (Zhai and Xu, 2021; Yin et al., 2024). Thus, auxin serves as a master regulator for the acquisition of pluripotency in callus cells by establishing an LRP-like genetic program (Sugimoto et al., 2010; Radhakrishnan et al., 2018; Chen et al., 2024).

Upon transfer from CIM to SIM, PLT3/5/7 activate the expression of *CUP-SHAPED COTYLEDON1* (*CUC1*) and *CUC2* in the pre-established pluripotent callus cells (Kareem et al., 2015), thereby initiating shoot regeneration. Subsequently, cytokinin signaling triggers the phosphorylation of B-type *Arabidopsis* RESPONSE REGULATORS (B-ARRs), which directly bind to the *WUSCHEL* (*WUS*) promoter to activate its transcription (Meng et al., 2017; Zubo et al., 2017). This activation is further reinforced through a complex formation between B-ARRs and HD-ZIP III transcription factors—PHABULOSA (PHB), PHAVOLUTA (PHV), and REVOLUTA (REV) (Zhang et al., 2017). The induced WUS protein ensures the proper expression of *SHOOT MERISTEMLESS* (*STM*), a master regulator of shoot apical meristem formation (Mayer et al., 1998); additionally, *STM* expression is also directly promoted by HD-ZIP III transcription factors (Shi et al., 2016). This coordinated gene regulatory network ultimately drives the *de novo* formation of the shoot apical meristem.

In contrast to hormone-dependent pathways, wounding can also induce callus formation without exogenous auxin. On hormone-free medium, wounding prompts *Arabidopsis* hypocotyls to form callus at cut sites. This is mediated by the transcription factor WOUND INDUCED DEDIFFERENTIATION *1* (*WIND1*), which triggers cytokinin biosynthesis, upregulates cytokinin-responsive cell-cycle genes like *CYCLIN D3* (*CYCD3*), and activates APETALA2/Ethylene Responsive Factor (AP2/ERF) regulators including *ERF115* and *PLT*s (Iwase et al., 2011; Ikeuchi et al., 2017). Although the pluripotency of such wound-induced callus has not been directly assessed (Ikeuchi et al., 2017), its regenerative capacity is evidenced by its ability to form shoots in certain tomato species (Harada et al., 2005). Furthermore, molecular evidence supports this regenerative potential: during the two-step shoot regeneration protocol, WIND transcription factors directly bind to the promoter of *ENHANCER OF SHOOT REGENERATION1* (*ESR1*) to activate its expression. *ESR1*, in turn, initiates a regulatory cascade that upregulates its paralog *ESR2*, as well as key shoot meristem genes such as *CUC1*, *STM*, and *WUS*, thereby promoting *de novo* shoot organogenesis (Iwase et al., 2017). These data indicate that at least a subset of callus induced by wounding, either alone or in combination with cytokinin, possesses the competence for shoot regeneration.

Indeed, the capacity for auxin-independent regeneration is further supported by one-step shoot regeneration protocols using a cytokinin-supplemented medium (i.e., MS medium containing cytokinin as the sole exogenous plant growth regulator) in various plant species (Mangat and Roy, 1986; Attfield and Evans, 1991; Kelkar and Krishnamurthy, 1998; Premkumar et al., 2011; Cappelletti et al., 2016; Alvarez et al., 2020; Shishodia et al., 2025). This one-step protocol parallels the auxin-independent, wound-induced callus formation described previously, and raises two intriguing questions. First, are LRP genes—which are typically induced by auxins to mark pluripotency in standard regeneration—also employed in this auxin-free context? Second, is the pivotal role of *WIND* genes, so clearly established in *Arabidopsis* wound-response and two-step regeneration, conserved during this direct organogenesis in other plants?

Petunia (*Petunia hybrida*) serves as a popular bedding plant and an established model in plant comparative biology (Gerats and Vandenbussche, 2005; Vandenbussche et al., 2016). In this species, *de novo* shoot regeneration from leaf discs can be achieved through a one-step protocol on a cytokinin-supplemented medium, a process initiated by callus formation at the wound sites (Rao et al., 1973; Vakili et al., 2019; Guo et al., 2021). This callus is likely induced by the combined stimuli of wounding and cytokinin signaling, analogous to the wound-induced callus in *Arabidopsis*. Thus, to investigate the molecular basis of this one-step regeneration pathway, we conducted a global gene expression analysis during shoot induction in petunia leaf discs on cytokinin-supplemented medium, with a particular focus on the expression of LRP genes. Additionally, we explored the function of *PhWIND1a* overexpression in petunia leaf tissue culture. This study provides insights into the divergent molecular mechanisms underlying different tissue culture systems across plant species.

## Materials and methods

### Plant materials and culture conditions

The plant material used was the *Petunia hybrida* inbred line ‘Mitchell Diploid’ (MD). The basal medium for plant tissue culture contained MS salts, B5 vitamins, 30 g/L sucrose, and 7 g/L agar (pH adjusted to 5.8 and autoclaved at 121 °C for 20 min).

Seeds were surface-sterilized in 2% NaClO (2% active chlorine) for 7 min, rinsed thoroughly with sterile deionized water five times, and sown onto solid half-strength MS medium for germination. After 14 days, the hypocotyls of sterile seedlings were cut in the middle, and the apical buds from the upper segments were transferred to fresh half-strength MS medium for subculture. Following another 21 days, the fifth leaf was excised, and leaf discs (0.8–1.0 cm²) from the middle leaf section were placed on full-strength MS medium supplemented with 2.5 μmol/L 6-benzylaminopurine (6-BA) to induce adventitious shoots.

All cultures were incubated at 24 ± 2 °C under a 16-h light/8-h dark cycle with a light intensity of 50 μmol m^-^² s^-^¹.

### Transcriptome sequencing and analysis

**Figure 1:**
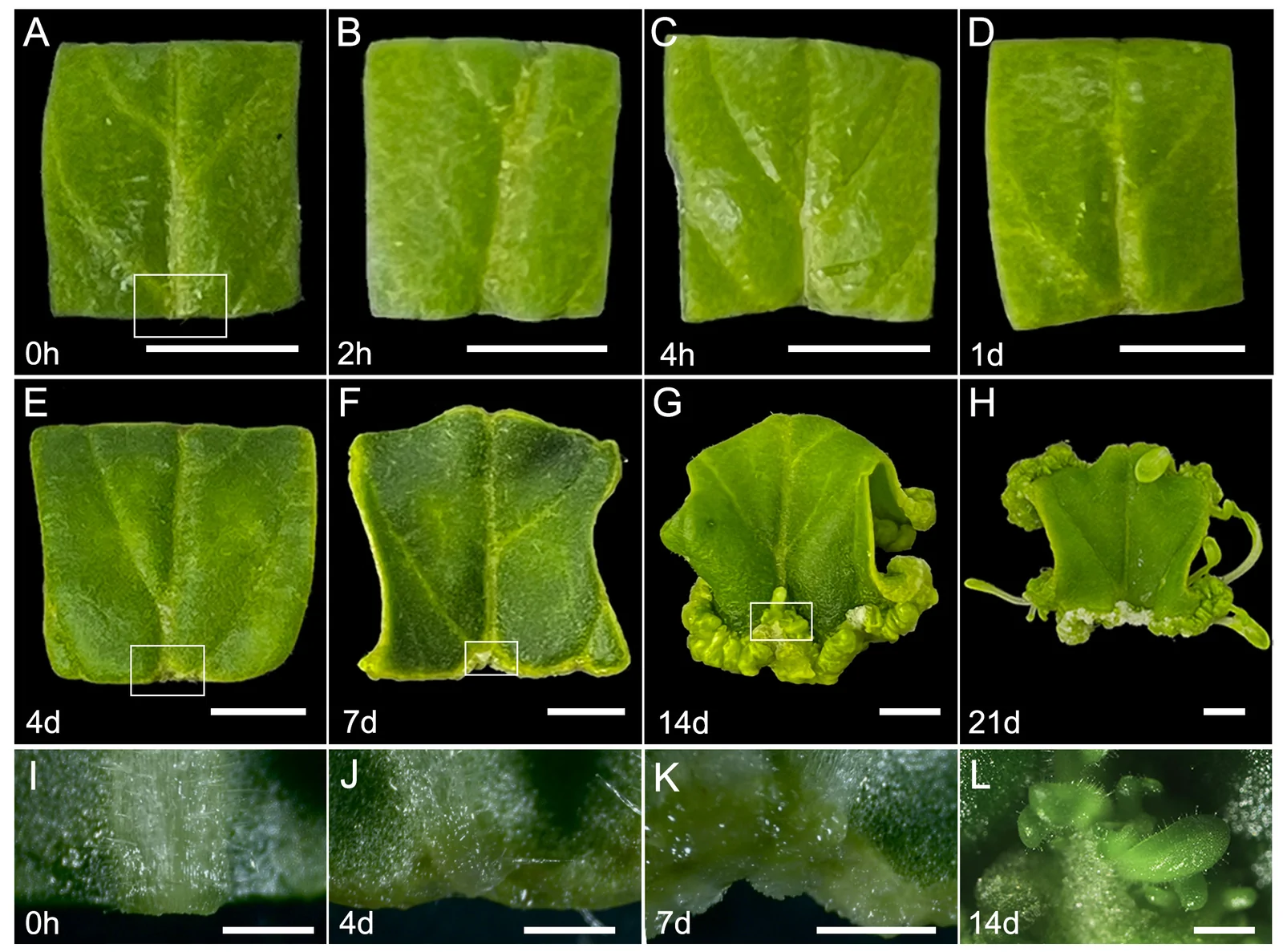
Time course of adventitious shoot regeneration and sampling time points for RNA-seq analysis. **(A–F)** Morphology of explants at the indicated time points for RNA-seq sampling. **(G, H)** Adventitious shoot formation at the cut sites of explants after 14 and 21 days of culture, respectively. **(I–L)** Morphological changes at the midrib region of leaf explants at different time points after culture. Samples for RNA-seq analysis were collected at 0 h, 2 h, 4 h, 1 d, 4 d, and 7 d. Scale bars: **(A–H)** = 0.5 cm; **(I–L)** = 0.1 cm.

To investigate the transcriptional changes during early adventitious shoot formation, we performed RNA-seq analysis on petunia leaf explants harvested at various time points after culture (0 h, 2 h, 4 h, 1 d, 4 d, and 7 d; **Figure 1**). For each time point, three independent biological replicates were collected, with each replicate consisting of approximately 200 mg of pooled leaf discs (five discs per replicate) ground in liquid nitrogen. Total RNA was extracted using Trizol reagent. RNA quality was assessed following a multi-step procedure: concentration and purity were measured with a Nanodrop2000, integrity was verified by agarose gel electrophoresis, and the RQN value was determined using an Agilent 5300 system.

RNA samples meeting the quality thresholds (concentration ≥ 30 ng/μL, RQN > 6.5, and OD260/280 ratio between 1.8 and 2.2) were used for library construction. Briefly, 1 μg of qualified total RNA was subjected to mRNA enrichment using oligo (dT) beads. The enriched mRNA was fragmented into approximately 300 bp pieces using fragmentation buffer. First-strand cDNA was synthesized using random primers, followed by second-strand synthesis to create double-stranded cDNA. The cDNA ends were repaired, adenylated at the 3’ ends, and then ligated to adapters. The adapter-ligated products were purified, size-selected, and amplified by PCR to generate the final sequencing libraries. The constructed libraries were quantified using a Qubit 4.0 and sequenced on a NovaSeq X Plus platform (PE150) with the NovaSeq Reagent Kit. The raw sequence data reported in this paper have been deposited in the Genome Sequence Archive (Genomics, Proteomics & Bioinformatics 2025) (Zhang et al., 2025) at the National Genomics Data Center, China National Center for Bioinformation/Beijing Institute of Genomics, Chinese Academy of Sciences (Nucleic Acids Res, 2026) (CNCB–NGDC Members and Partners et al., 2026), under accession number CRA044133, and are publicly accessible at https://ngdc.cncb.ac.cn/gsa.

For read alignment, the sequenced reads were quality-controlled and then aligned to the *Petunia axillaris* (v1.6.2) reference genome using HiSat2 (http://ccb.jhu.edu/software/hisat2/index.shtml). This genome was selected because the MD accession used in this study derives >78% of its genes from *P. axillaris* (Bombarely et al., 2016), and preliminary analysis showed a high mapping rate of >93%. Gene and transcript abundance were quantified using RSEM (https://github.com/deweylab/RSEM). Differential gene expression analysis between samples was performed with DESeq2 (https://bioconductor.org/packages/release/bioc/html/DESeq2.html), and genes with |log2FoldChange| ≥ 1 and an FDR < 0.05 were defined as significantly differentially expressed genes (DEGs). Functional annotation of the DEGs was performed for GO terms and KEGG pathways using the GO (Version 2022.0915) and KEGG (Version 2022.10) databases, respectively. GO functional enrichment and KEGG pathway analysis were carried out by Goatools and Python scipy software, respectively. All bioinformatics analyses were conducted on the Majorbio Cloud Platform (https://cloud.majorbio.com/).

### Selection of cytokinin and auxin metabolism and signaling genes

Genes involved in cytokinin and auxin biosynthesis were retrieved from the PaxillarisCyc database (version 3.0.0; https://pmn.plantcyc.org/PAXILLARIS/NEW-IMAGE?type=ECOCYC-CLASS&object=Plant-Hormone-Biosynthesis) . Genes associated with cytokinin and auxin signaling were identified based on KEGG pathway annotations.

### Prediction of regeneration-associated genes

Putative regeneration-associated genes in petunia were identified through a homology-based approach. Protein sequences of *Arabidopsis thaliana* genes known to be involved in *in vitro* regeneration were used as queries in BLASTP searches against the annotated protein sequences of the *Petunia axillaris* draft genome (v1.6.2). For each query, the top 10 most similar petunia sequences were retrieved. These sequences were then combined with homologous genes identified by blasting other known regeneration-associated genes from the same family. After removing duplicates, phylogenetic trees were constructed. Putative orthologs of *Arabidopsis* regeneration genes in petunia were identified as follows: In cases of one-to-one correspondence, the single petunia gene was designated as the ortholog. For *Arabidopsis* genes lacking a one-to-one ortholog (e.g., those with closer paralogs in *Arabidopsis*), the ortholog was defined as the petunia gene residing within the same orthogroup as the query *Arabidopsis* gene.

*Arabidopsis* genes involved in *in vitro* regeneration reported prior to 2019 were primarily compiled from the review by Ikeuchi et al (Ikeuchi et al., 2019). Genes identified after 2019 were collected by searching the literature using the keyword “Plant regeneration”.

### Nomenclature of petunia regeneration-associated genes

Genes were named following a hierarchy of criteria that integrated phylogenetic tree analysis with *Arabidopsis* and the *Petunia axillaris* genome annotation. First, the standard prefix “A” for *Arabidopsis* was changed to “Ph” (e.g., *ARR* became *PhRR*). If phylogenetic analysis confirmed a one-to-one orthology, the gene was named after its *Arabidopsis* ortholog (e.g., *PhARF5*). For *Arabidopsis* genes that lacked a one-to-one ortholog and were instead associated with a clade containing multiple petunia paralogs, these petunia genes were assigned sequential alphanumeric suffixes based on their phylogenetic relationship (e.g., *PhWIND1a*, *PhWIND1b*). The annotated gene name from the *Petunia axillaris* genome was preferentially retained when it did not conflict with the phylogeny-based naming.

### RT-qPCR and RT-PCR analysis of differentially expressed genes

Total RNA was extracted as described for transcriptome sequencing. For validation of the RNA-seq data, the same RNA samples used for sequencing were analyzed by RT-qPCR. For all other RT-qPCR and RT-PCR analyses, we used RNA extracted from independently harvested biological replicates. Complementary DNA (cDNA) was synthesized from mRNA using a TransScript All-in-One First-Strand cDNA Synthesis SuperMix kit (TransGen Biotech, AE311-03) following the manufacturer’s protocol.

For RT-qPCR, amplification was performed on a Bio-Rad CFX96 real-time PCR system with the following thermal cycling profile: initial denaturation at 95 °C for 30 s; 40 cycles of 95 °C for 5 s and 60 °C for 5 s; followed by melt curve analysis to confirm amplification specificity. Relative expression levels were calculated using the comparative 2^-ΔΔCt^ method (Schmittgen and Livak, 2008). Two reference genes, *ACTIN* and *RPS13*, were used for normalization.

For RT-PCR analysis of root and shoot meristem genes, leaf discs were cultured on full-strength MS medium supplemented with 0.5 mg/L 6-BA (approximately 2.2 μmol/L) or 1.0 mg/L NAA (approximately 5.4 μmol/L) and harvested at various time points (0 h, 2d, 4 d, 7 d and 10d) for RNA extraction. Amplifications were performed using EasyTaq DNA Polymerase. The cycling conditions were: 94 °C for 5 min; 30 and 35 cycles of 94 °C for 30 s, 57–60 °C (depending on primer Tm) for 30 s, and 72 °C for 30 s; followed by a final extension at 72 °C for 10 min. This 6-BA concentration (0.5 mg/L) is biologically equivalent to the 2.5 μmol/L 6-BA used in the RNA-seq experiments, based on our routine regeneration assays. The NAA-treated samples were included as internal controls to confirm that the absence of PCR products in cytokinin-treated samples was not due to technical failure, and to verify the overall functionality of the PCR system. Primer sequences for both RT-qPCR and RT-PCR are provided in **Supplementary Table 1**.

### Generation and functional analysis of *PhWIND1a* overexpression lines

The complete coding sequence of *PhWIND1a* was amplified by PCR from the cDNA of MD plants, using forward primer 5′-catgccATGGCAGCAGCTATGGA-3′ (with the *Nco*I site underlined) and reverse primer 5′-cgggatccTTATAGAGAGGCCCAATC-3′ (with the *BamH* I site underlined), and cloned into the pMD19 (simple) T-vector (Takara, Dalian, China) for sequencing. Following sequence verification, the *PhWIND1a* fragment was excised using *Nco*I and *Bam*H I sites and subcloned into a modified pGreenII0229 vector, placing it under the control of a double 35S promoter and a 35S terminator amplified from pSAT6-RFP-N1 (Tzfira et al., 2005). The resulting overexpression construct (2×35S:*PhWIND1a*) was introduced into *Agrobacterium tumefaciens* strain GV3101 via electroporation and subsequently transformed into petunia as previously described (Zhang et al., 2016a).

Plants exhibiting BASTA resistance were transferred to the greenhouse for phenotypic observation. Transgene integration was confirmed by PCR using primers specific to the *PhWIND1a* coding sequence (5′-GAAGAGCATTGACTCCAAGACGAAGA-3′) and the 35S terminator (5′-CGAAACCCTATAAGAACCCTA-3′). The PCR cycling conditions were: 94 °C for 5 min; 30 cycles of 94 °C for 30 s, 53 °C for 30 s, and 72 °C for 45 s; followed by a final extension at 72 °C for 10 min. The expression level of *PhWIND1a* in transgenic plants was then determined by RT-qPCR, and independent lines with varying expression levels were selected for further analysis.

Leaf explants from transgenic plants were cultured on hormone-free MS medium or MS medium supplemented with 2.5 μmol/L 6-BA. For each line and treatment, 30 leaf discs were used as explants (six replicate culture vessels, each containing five leaf discs). Callus formation was defined as the emergence of amorphous cell masses >1 mm in diameter from the wounded sites of explants after four weeks of culture, and shoot regeneration was defined as the development of a structure with a meristem and at least two young leaves. After documentation, callus size was measured using ImageJ software.

The significance of differences between transgenic lines and the wild-type (WT) in callus formation frequency, callus size, and adventitious shoot number per explant was analyzed using Jamovi (version 2.6). For each parameter, normality and homogeneity of variances were first assessed using the Shapiro–Wilk test and Levene’s test, respectively. If the data met both assumptions, statistical significance was evaluated using Fisher’s ANOVA; if variances were unequal, Welch’s ANOVA was applied. For data that deviated from normality, the non-parametric Kruskal–Wallis test was used. The expression level of *PhWIND1a* in the induced callus was additionally validated by RT-qPCR.

## Results

### Transcriptome dynamics during early adventitious shoot regeneration on cytokinin-supplemented medium

When cultured on MS medium supplemented with 2.5 μmol/L 6-BA, leaf disc explants of petunia ‘Mitchell Diploid’ (MD) formed visible shoot primordia within 14 days (**Figure 1**). To capture the transcriptional dynamics underlying this process, we profiled the transcriptome of explants harvested at these key time points (0 h, 2 h, 4 h, 1 d, 4 d, and 7 d; as illustrated in **Figure 1**). DEGs were identified by comparing each time point against the 0-hour control, applying a threshold of |log2FoldChange| > 1 and an adjusted p-value (*P*_adjust_) < 0.01 (Benjamini-Hochberg procedure for FDR control). This analysis identified a total of 10,821 DEGs. To validate the reliability of the RNA-seq data, we performed RT-qPCR analysis on several genes, including *PhCYCD3*s—key downstream targets of the cytokinin signaling pathway known to promote cell proliferation through transcriptional up-regulation (Riou-Khamlichi et al., 1999; Müller and Sheen, 2007). The results confirmed the expression trends observed in the sequencing data (**Supplementary Figure 1**).

**Figure 2:**
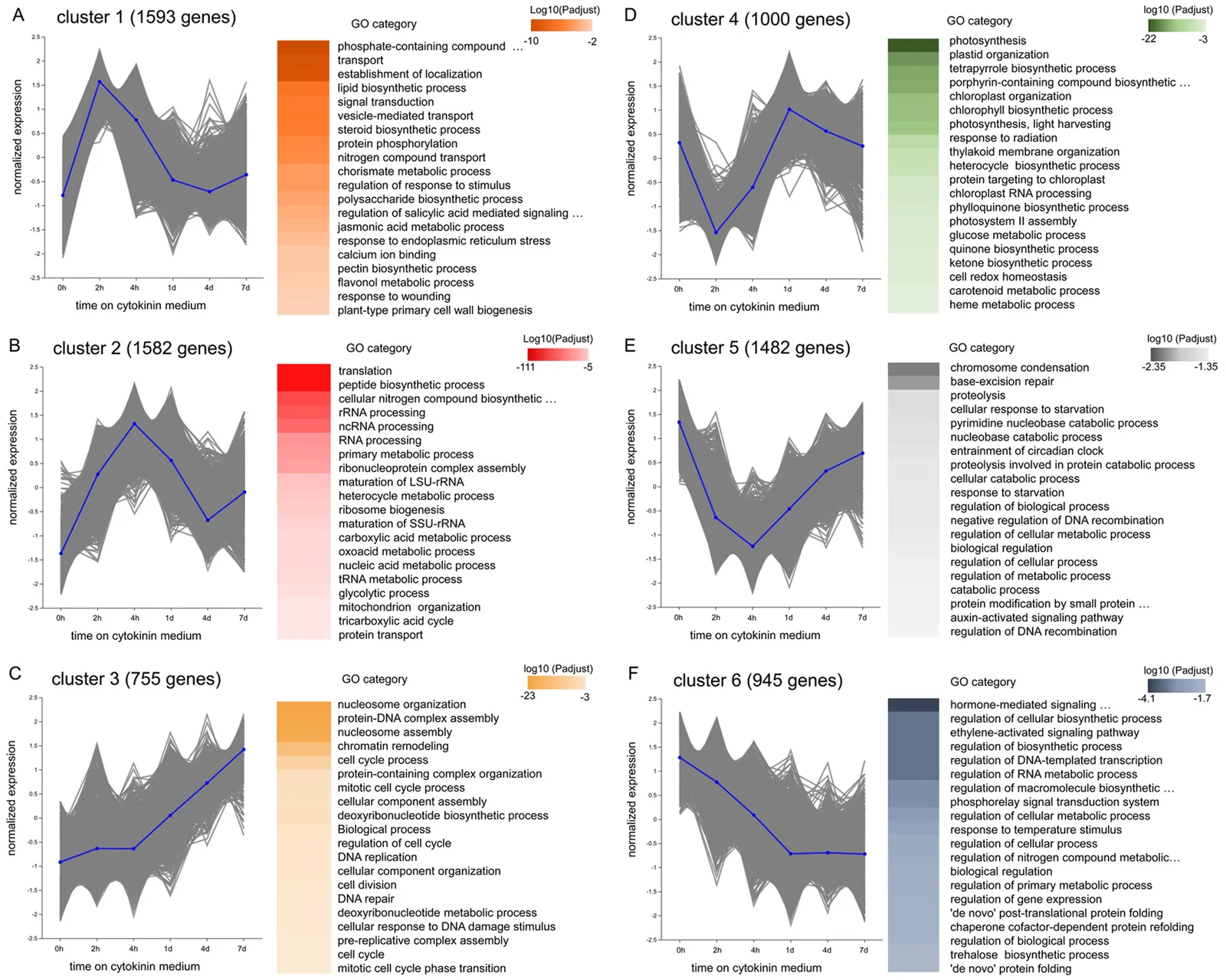
Clustering analysis of time-course RNA-seq reveals six characteristic transcriptional changes during shoot regeneration. **(A)** Cluster 1 contains 1,593 genes that are transiently induced within 2 h. GO categories related to cell membrane and wall repair, as well as wound response, are overrepresented. **(B)**, Cluster 2 includes 1,582 genes induced at 4 h. GO terms of protein synthesis, nucleotide and energy metabolism are enriched. **(C)**, Cluster 3 comprises 755 genes induced after 4 h. GO categories such as DNA replication and cell cycle are overrepresented. **(D)**, The 1,000 genes in cluster 4 are initially down-regulated but subsequently up-regulated. Photosynthesis-related GO terms are enriched. **(E)**, Cluster 5 contains 1,482 genes that are down-regulated and then recovered. GO categories of negative regulation of cell division are overrepresented. **(F)**, Cluster 6 includes 945 genes that are down-regulated after culture. Hormone signaling pathways are enriched. Blue lines indicate the centroid of each cluster. Top 20 representative GO categories are shown with color codes representing the log_10_ (*P*_adjust_).

To delineate the overarching expression dynamics, we focused on 7,357 genes with a transcripts per million (TPM) value > 1 in at least one time point for K-means clustering. After evaluating different cluster numbers, we determined that partitioning the genes into six clusters optimally captured the major temporal expression patterns (**Figure 2**; **Supplementary Table 2**). These clusters effectively represent distinct transcriptional programs activated during organogenesis.

Cluster 1, comprising 1,593 genes, exhibited rapid induction upon culture of leaf disc explants, with expression levels peaking within 2 hours (**Figure 2A**). Gene Ontology (GO) enrichment analysis revealed a significant overrepresentation of terms related to cellular membrane and cell wall repair processes, including phosphate-containing compound metabolic process (*P*_adjust_ = 1.00E-10), establishment of localization (4.66E-10), and lipid biosynthetic process (1.80E-7), as well as polysaccharide biosynthetic process (7.58E-5) and pectin biosynthetic process (3.20E-3). As expected for the wounding response inherent to explant excision, GO terms associated with stress and signaling were also highly enriched, such as regulation of response to stimulus (2.72E-5), response to wounding (8.00E-3), and the biosynthesis of key signaling molecules like jasmonic acid (jasmonic acid metabolic process, 4.10E-4). Interestingly, we also found a significant enrichment for the chorismate biosynthetic process (2.55E-5), a precursor for salicylic acid, and calcium ion binding (3.20E-3), indicating the involvement of multiple signaling pathways in this early transcriptional response.

Cluster 2 encompassed 1,582 genes whose expression peaked at 4 hours (**Figure 2B**). These genes were overwhelmingly associated with terms broadly related to primary metabolism, including primary metabolic process (*P*_adjust_ = 6.72E-41). This encompassed essential cellular functions such as protein synthesis, with highly enriched terms for translation (1.84E-111), RNA processing (3.12E-44), and ribosome biogenesis (4.72E-18). Key energy metabolism pathways were also strongly represented, including glycolytic process (1.06E-9), tricarboxylic acid (TCA) cycle (3.99E-6), and carboxylic acid metabolic process (1.59E-12), alongside nucleic acid metabolic process (5.62E-12). This pattern indicates that fundamental cellular metabolism was activated under our culture conditions, reaching its peak at 4 hours and subsequently maintaining a higher level than in the initial leaf explants throughout the culture period.

A distinct temporal pattern was observed for cluster 3 (755 genes), where expression began to rise at 4 hours and continued throughout the 7-day culture (**Figure 2C**). This cluster was strongly linked to DNA replication and cell division, including nucleosome organization (*P*_adjust_ = 9.28E-23), cell cycle process (1.34E-11), and DNA replication (3.29E-5). This expression pattern indicates that active cell division was initiated around 1 day of culture and intensified thereafter.

In contrast, cluster 4 (1,000 genes) displayed an initial suppression, with expression reaching a nadir at 2 hours before recovering and exceeding baseline by day 1 (**Figure 2D**). This cluster was notably enriched for photosynthesis-related terms, such as photosynthesis (1.94E-22), chlorophyll biosynthetic process (3.96E-14), and response to radiation (9.38E-10). This pattern suggests a transient disruption and subsequent reinforcement of photosynthetic capacity.

Like cluster 4, cluster 5 (1,482 genes) showed an initial decline, bottoming at 4 hours before gradually recovering (**Figure 2E**). This cluster showed enrichment for GO terms such as chromosome condensation (*P*_adjust_ = 4.90E-3), proteolysis (1.70E-2), and negative regulation of DNA recombination (2.90E-2), reflecting the dynamics of negative regulators involved in cell division and metabolic processes. Notably, the auxin-activated signaling pathway (4.30E-2) was also enriched in this cluster, suggesting a potential transient suppression of auxin response during the early culture stages on the cytokinin-supplemented medium.

Finally, cluster 6 (945 genes) was defined by a sustained downregulation over the first 24 hours (**Figure 2F**). Significant GO enrichments in this cluster included hormone-mediated signaling pathway (9.80E-5) and the ethylene-activated signaling pathway (4.20E-4). Key genes within this cluster comprised ethylene response factors (*ERF*s), auxin signaling components such as IAAs and auxin response factors (*ARF*s), as well as genes encoding the abscisic acid receptor PYR1 and brassinosteroid signaling regulators like the BZR1 family proteins. This pattern likely reflects the complex crosstalk between cytokinin and other hormonal pathways during the regeneration process.

In summary, the transcriptomic profiling of petunia leaf explants cultured on a cytokinin-supplemented medium revealed a coordinated sequence of events: an initial wound and stress response was followed by the activation of primary metabolism and cell division. Concurrently, negative regulators of cell division and metabolism were transiently suppressed before recovering to a steady state. Photosynthesis-related genes exhibited a transient down-regulation followed by sustained up-regulation, while genes involved in multiple hormone signaling pathways were generally suppressed.

### Dynamics of cytokinin biosynthesis and signaling genes

Auxin and cytokinin responses are pivotal factors determining plant *in vitro* regeneration. The petunia genome encodes key components of the cytokinin pathway, including 7 isopentenyl transferases (*IPT*s), 2 cytochrome P450 monooxygenases (*CYP735A*s), 11 *LONELY GUY* (*LOG*) genes, and 7 cytokinin oxidases (*CKX*s) (**Supplementary Table 3**). Transcriptome sequencing of leaf discs over 0–7 days of culture revealed distinct expression patterns among these genes. Of these, three *IPT*s, six *LOG*s, and six *CKX*s showed substantial expression (TPM >1 in at least one time point; **Supplementary Table 3**). Furthermore, nine of these expressed genes exhibited significant differential expression (FDR < 0.01; **Table 1**). Most *CKX* genes were upregulated, whereas most *LOG*s were downregulated, collectively indicating activated cytokinin degradation and feedback inhibition of biosynthesis on the cytokinin-containing medium. Notably, *PhLOG3* was transiently upregulated at 2 hours, potentially associated with wound-induced cytokinin synthesis. In contrast, *PhIPT1a* was markedly induced at days 4 and 7, likely linked to localized endogenous cytokinin production that promotes shoot apical meristem gene expression.

**Table 1:** Heat-map representation of cytokinin-induced transcriptional changes for genes implicated in cytokinin metabolism and signaling (compare to 0h and *P*_adjust_ < 0.01).

Seq_id	Name	KO_name	Fold change (log_2_FC)^a^				
			2h	4h	1d	4d	7d
Peaxi162Scf00106g00224	*PhIPT1a*	IPT				3.9730	4.9979
Peaxi162Scf00438g00511	*PhLOG1b*	LOG		-1.3510	-2.3999	-2.5713	-2.7498
Peaxi162Scf00270g00019	*PhLOG3*	LOG	1.0460	-2.6299	-2.7017	-2.5031	-1.6138
Peaxi162Scf00123g00047	*PhLOG4a*	LOG					-1.1150
Peaxi162Scf01278g00007	*PhLOG4b*	LOG		-1.9263			-1.5952
Peaxi162Scf00498g00427	*PhCKX3a*	CKX	6.1749	6.5014	6.7442	6.5730	5.3602
Peaxi162Scf00189g00922	*PhCKX5*	CKX	1.5575	1.3734	2.4740	1.0224	
Peaxi162Scf01115g00115	*PhCKX6*	CKX	2.0477	2.7625			
Peaxi162Scf00101g01910	*PhCKX7*	CKX	1.2474		1.0961	1.0074	1.0428
Peaxi162Scf00776g00011	*PhHK2*	AHK2_3_4	-1.2489				
Peaxi162Scf00128g01329	*PhHK3*	AHK2_3_4	-1.8980	-2.5998	-1.4425	-1.4929	-1.4697
Peaxi162Scf00337g00622	*PhHK4*	AHK2_3_4	1.2592	1.8791	1.8778	1.9453	2.3789
Peaxi162Scf00434g00728	*PhHP1a*	AHP		-2.9580			2.2942
Peaxi162Scf01039g00232	*PhHP1c*	AHP	-1.2274	-1.7800			
Peaxi162Scf00006g03911	*PhHP1d*	AHP					-1.0590
Peaxi162Scf00247g00048	*PhHP4a*	AHP	-2.8766	-3.3619	-4.5971	-4.1566	-5.6935
Peaxi162Scf00699g10015	*PhHP4b*	AHP	-1.4085	-1.9174	-3.4932	-5.4722	-5.3492
Peaxi162Scf00828g00013	*PhRR3*	ARR-A	2.8705	2.6776	1.9812	1.5390	1.5942
Peaxi162Scf00496g00025	*PhRR6*	ARR-A	2.4953	2.7076			
Peaxi162Scf00570g00004	*PhRR9b*	ARR-A	2.4629	1.8746			
Peaxi162Scf01029g00212	*PhRR9c*	ARR-A	1.5360	1.0922		1.6507	1.7310
Peaxi162Scf01378g00113	*PhRR9d*	ARR-A				1.0201	
Peaxi162Scf00017g02232	*PhRR15*	ARR-A	1.3371	3.1481	2.3707	1.6746	2.0928
Peaxi162Scf00304g00619	*PhRR17a*	ARR-A		1.4491	1.5989	2.2717	
Peaxi162Scf00739g00426	*PhRR17b*	ARR-A	4.6480	4.1600	3.6045	3.1094	3.0152
Peaxi162Scf00605g00513	*PhRR17c*	ARR-A	4.0029	3.2603		4.8577	4.1746
Peaxi162Scf00337g00718	*PhRR2*	ARR-B	-1.3199				
Peaxi162Scf00128g00723	*PhRR11*	ARR-B	-1.6447	-2.6616	-1.4012	-1.2037	
Peaxi162Scf00108g00420	*PhRR12a*	ARR-B		-1.0031			
Peaxi162Scf00347g00627	*PhRR12b*	ARR-B	1.5205				
Peaxi162Scf00312g00089	*PhRR19c*	ARR-B	-1.7497	-1.8831	-1.4834		
Peaxi162Scf00497g00086	*PhRR19d*	ARR-B		-1.3555			

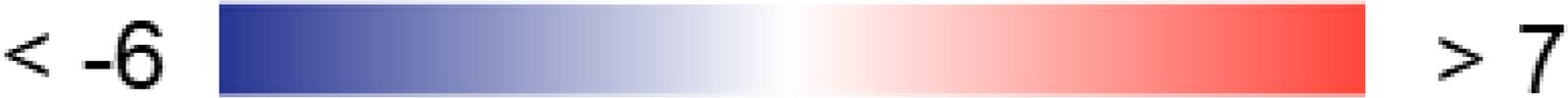

^a^ Only genes with |log2FC|≥1 and *P*_adjust_ *≤* 0.01 are presented.

KEGG and phylogenetic analyses annotated 34 cytokinin signaling genes in the petunia genome (**Supplementary Table 3**). Of these, 29 had TPM >1 during culture, and 23 showed significant expression changes (**Table 1**). Type-A response regulators (*RR*s) were uniformly upregulated, while most type-B *RR*s were downregulated (**Table 1**). Among the histidine kinases (HKs), *PhHK4* was persistently upregulated, whereas *PhHK2* and *PhHK3* were downregulated. Most histidine phosphotransfer protein (HP) genes were downregulated, except for *PhHP1a*, which was significantly upregulated by day 7. Collectively, our data suggest that the expression dynamics of cytokinin signaling genes are dually regulated by hormonal feedback and developmental programming.

### Dynamics of auxin biosynthesis and signaling genes

The primary auxin biosynthesis pathway in plants utilizes tryptophan as a substrate, involving two key enzymatic steps: tryptophan aminotransferase (TAA) and YUCCA (YUC) flavin monooxygenase. TAA catalyzes a reversible reaction, while YUC acts as the rate-limiting enzyme (Mashiguchi et al., 2011; Won et al., 2011; Zhao, 2012; Solanki and Shukla, 2023). Auxin degradation is primarily regulated by genes such as 2-oxoglutarate-dependent dioxygenase (DAO) (Porco et al., 2016; Zhang et al., 2016b; Solanki and Shukla, 2023). The petunia genome contains 3 TAA, 19 YUC, and 3 DAO genes. Of these, only seven genes showed substantial expression (TPM > 1; **Supplementary Table 4**), and five exhibited significant differential expression during the culture period (**Table 2**). KEGG and phylogenetic analyses annotated 178 genes involved in auxin signaling. Of these, 102 were substantially expressed (TPM > 1; **Supplementary Table 4**), with 49 showing significant expression changes in at least one time point. These included nine SAUR genes, which are not discussed further due to functional redundancy among the many family members (Zhang et al., 2021a). Expression changes for the remaining 40 genes are shown in **Table 2**.

**Table 2:** Heat-map representation of cytokinin-induced transcriptional changes for genes implicated in auxin metabolism and signaling (compare to 0h and *P*_adjust_ < 0.01).

Seq_id	Name	KO_name	Fold change (log_2_FC)^a^				
			2h	4h	1d	4d	7d
Peaxi162Scf00067g00414	*PhYUC10a*	YUCCA	-1.8278		-1.2553	-1.7960	-2.6909
Peaxi162Scf00876g00114	*PhYUC10b*	YUCCA			-1.5005		-1.1326
Peaxi162Scf00089g01520	*PhTAA1a*	TAA1	1.7403	1.2238			
Peaxi162Scf00128g00028	*PhTAR2*	TAA1					4.1773
Peaxi162Scf00981g00113	*PhDAO1a*	DAO		-1.1698			
Peaxi162Scf00042g02726	*PhLAX2a*	AUX1, LAX			2.2126	3.3785	3.5419
Peaxi162Scf00362g00846	*PhLAX2b*	AUX1, LAX	-2.3657	-1.1491		1.1098	1.6848
Peaxi162Scf00683g00461	*PhPIN1a*	PIN	-3.8162	-2.2296	-1.3338		
Peaxi162Scf00003g04923	*PhPIN1b*	PIN	-2.3877			2.4378	3.2437
Peaxi162Scf00159g00068	*PhPIN3a*	PIN	-1.8777	-2.1230	-1.8780	-1.2422	
Peaxi162Scf00253g01129	*PhPIN3b*	PIN	-3.8852	-3.1511	-2.2546	-2.6504	-2.2317
Peaxi162Scf00976g00113	*PhPIN5a*	PIN				4.2064	5.0686
Peaxi162Scf00033g00711	*PhPIN6*	PIN	-2.8772			3.1301	3.0749
Peaxi162Scf00152g01635	*PhARF1c*	ARF	-2.0282	-1.7187	-1.5463	-1.5691	-1.2338
Peaxi162Scf00099g00127	*PhARF3*	ARF	-1.2309				
Peaxi162Scf00287g00193	*PhARF5*	ARF			1.1053	2.2578	3.3217
Peaxi162Scf00345g00027	*PhARF9a*	ARF	-1.6168	-2.2359	-1.3901	-1.4992	
Peaxi162Scf00074g00537	*PhARF9b*	ARF	-3.4223	-3.1751	-3.5368	-3.5215	-2.6101
Peaxi162Scf00547g00610	*PhARF19*	ARF	-1.2276	-1.4404	-1.8417	-1.8500	-1.4831
Peaxi162Scf01306g00016	*PhGH3.5a*	GH3	2.9300	1.2359			
Peaxi162Scf00007g01617	*PhGH3.5b*	GH3	-4.1666	-3.7274	-3.8050		
Peaxi162Scf00572g00811	*PhIAA1a*	IAA		-3.6339	-2.8496		-2.7538
Peaxi162Scf01311g00016	*PhIAA1b*	IAA		-2.1894	-2.9154	-2.7643	-4.0263
Peaxi162Scf00049g02220	*PhIAA1c*	IAA	-1.4781	-2.3035	-2.7118	-2.2852	-2.6605
Peaxi162Scf00578g00523	*PhIAA3b*	IAA	-1.8070	-1.6319	-2.0881	-1.6288	-1.7238
Peaxi162Scf00207g00839	*PhIAA6b*	IAA	-2.4428	-2.2209	-2.5062		2.0869
Peaxi162Scf00015g00237	*PhIAA6c*	IAA	-2.4589	-3.0006		-2.6374	-6.2092
Peaxi162Scf00034g01013	*PhIAA6d*	IAA	-3.5904	-2.6004	-2.7764	-4.3713	-3.3739
Peaxi162Scf01044g00226	*PhIAA13b*	IAA		-1.9139	-2.0944	-1.1255	
Peaxi162Scf01416g00118	*PhIAA14a*	IAA				-1.4849	-2.4518
Peaxi162Scf00578g00316	*PhIAA14b*	IAA	-2.0570	-2.8246	-3.4823	-3.0811	-3.9343
Peaxi162Scf00282g00515	*PhIAA16a*	IAA	-1.9189				-1.0105
Peaxi162Scf00572g00812	*PhIAA16b*	IAA	-2.3905	-3.9261	-4.8272	-3.9031	-4.7170
Peaxi162Scf00015g00015	*PhIAA27a*	IAA	-1.2303	-1.2679	-1.1892	-1.2278	-1.5222
Peaxi162Scf00665g00013	*PhIAA27c*	IAA	-1.9389			1.4763	1.8093
Peaxi162Scf00643g00021	*PhIAA29b*	IAA	-3.1021	-2.9276	-2.3447	-1.9225	-2.4252
Peaxi162Scf00088g01715	*PhIAA29c*	IAA	-2.2770	-2.3880	-2.5974		

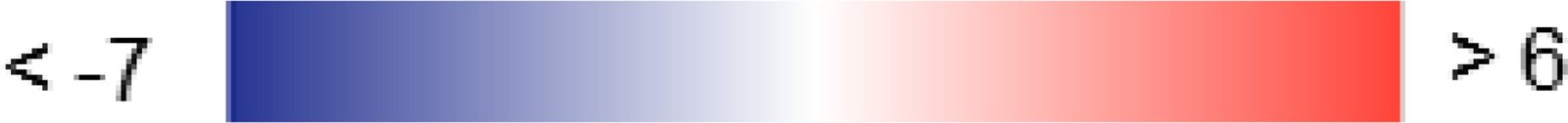

^a^ Only genes with |log2FC|≥1 and *P*_adjust_ *≤* 0.01 are presented.

Analysis of expression trends revealed an overall suppression of auxin response during early culture stages: key biosynthetic genes (e.g., YUC) were downregulated, and early auxin response genes (*Aux*/*IAA*s) as well as *PIN* auxin efflux carriers were suppressed within the first day. However, after 4 days of culture, a distinct shift was observed. The early response genes *PhIAA6b* and *PhIAA27c*, along with *LAX* auxin influx (*PhLAX2a*, *PhLAX2b*) and efflux carriers (*PhPIN1b*, *PhPIN5a*, *PhPIN6*), were markedly upregulated (**Table 2**), suggesting a reactivation of auxin response and potential auxin polar transport in specific regions. (*PIN* stands for *PIN*-*FORMED*; *LAX* stands for *LIKE AUXIN RESISTANT*).

Notably, *PhARF5* was the sole *ARF* member significantly upregulated throughout the culture (**Table 2**). Given that *ARF* activity is not strictly dependent on transcript abundance, the increased *PhARF5* expression under low-auxin conditions (without exogenous auxin) is notable in the context of cell division and callus formation, suggesting a potential association with adventitious shoot regeneration in this system.

Collectively, these findings outline a biphasic auxin response: an initial broad suppression followed by the specific activation of key transporters and *PhARF5*, which is critical for regeneration.

### Dynamics of shoot regeneration regulator genes

Plant *in vitro* regeneration is a complex and diverse process, implying the involvement of numerous genes. Through literature mining, we compiled 134 regeneration-associated genes (**Supplementary Table 5**). Homology-based analysis (BLAST and phylogeny) identified 175 petunia orthologs of these *Arabidopsis* genes (**Supplementary Table 5**; see **Supplementary Document 1** for phylogenetic trees). The expression of 44 genes involved in cytokinin and auxin metabolism and signaling has been described previously. The expression profiles of the remaining 131 genes are listed in **Supplementary Table 6**, with 78 showing significant differential expression (FDR < 0.05) during culture.

In *Arabidopsis*, adventitious shoot regeneration hinges on cellular reprogramming for pluripotency and shoot meristem formation. We defined 33 key *Arabidopsis* transcription factors governing these processes as Core Shoot Regeneration Regulators (CSRRs), including *WIND1-4*, *LBD16*/*17*/*18*/*29*, *PLT1*/*2*/*3*/*5*/*7*, *ERF113*/*114*/*115*, *ESR1*/*2*, *SCR*, *SHR*, *WOX5*/*7*/*11-14*, *WUS*, *PHB*, *PHV*, *REV*, *CUC1*/*2*, and *STM* (**Supplementary Document 1**). We propose their expression dynamics can serve as indicators of mechanistic differences between regeneration systems. In petunia genome we identified 31 genes which were putative orthologs of *Arabidopsis* CSRRs (**Supplementary Table 5**). Among them, 19 were differentially expressed (**Table 3**), suggesting potential functional conservation across species and regeneration systems.

**Table 3:** Heat-map representation of cytokinin-induced transcriptional changes for core shoot regeneration regulator genes (compare to 0h and *P_adjust_* < 0.05). .

Seq_id	Name	*Arabidopsis* homolog	Classification	Fold change (log_2_FC)^a^				
				2h	4h	1d	4d	7d
Peaxi162Scf01142g00028	*PhPLT3a*	*PLT3/7*	AP2/ERF				3.5698	4.4439
Peaxi162Scf00085g02316	*PhPLT3b*	*PLT3/7*	AP2/ERF					2.4938
Peaxi162Scf00196g00322	*PhPLT3c*	*PLT3/7*	AP2/ERF				4.5095	6.3128
Peaxi162Scf00231g00729	*PhPLT5*	*PLT5*	AP2/ERF	-2.3670				
Peaxi162Scf00041g00061	*PhWIND1a*	*WIND1/2*	AP2/ERF					-1.4627
Peaxi162Scf00160g00148	*PhWIND1b*	*WIND1/2*	AP2/ERF		-1.6140	-1.4353	-1.4226	-1.5932
Peaxi162Scf00422g00012	*PhWIND1c*	*WIND3/4*	AP2/ERF			-1.4570	-1.0470	
Peaxi162Scf00314g00628	*PhERF113a*	*ERF113/114/115*	AP2/ERF		2.2584	2.5037	2.5984	3.0596
Peaxi162Scf00135g01433	*PhERF113b*	*ERF113/114/115*	AP2/ERF			4.4910	4.1782	4.5964
Peaxi162Scf00128g10022	*PhESR1a*	*ESR1/2*	AP2/ERF				5.4112	7.5121
Peaxi162Scf00374g00723	*PhSCR*	*SCR*	GRAS					2.7102
Peaxi162Scf00267g00059	*PhPHB*	*PHB/PHV*	HD-ZIPIII	-1.8533	-1.7158			2.0714
Peaxi162Scf00021g01519	*PhLBD18a*	*LBD18/30*	LOB		-2.3783			
Peaxi162Scf00238g01417	*PhLBD18c*	*LBD18/30*	LOB					4.6449
Peaxi162Scf00069g01832	*NAM*	*CUC1/2*	NAC					6.8385
Peaxi162Scf00708g00039	*PhSTM*	*STM*	KNOX					3.9458
Peaxi162Scf00122g02520	*PhWOX13*	*WOX13/14*	WOX		-1.2933			
Peaxi162Scf01475g00015	*PhWOX14*	*WOX13/14*	WOX					1.6555
Peaxi162Scf00083g00516	*PhWUS/TER*	*WUSCHEL*	WOX				5.7794	7.7700

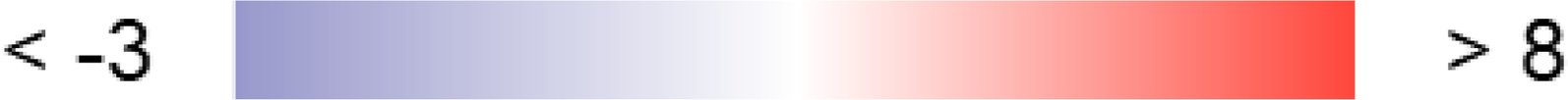

^a^ Only genes with |log2FC|≥1 and *P_adjust_ ≤* 0.05 are presented, with the exception of *PhSTM* (*P_adjust_* = 0.0521, *P* value = 0.0185).

*WIND* genes in *Arabidopsis* are wound-induced master regulators that promote regeneration potentially by enhancing cytokinin signaling and activating *ESR1* (Iwase et al., 2011, Iwase et al., 2017). The three *WIND* orthologs in petunia did not show significant upregulation during regeneration (**Table 3**; **Supplementary Table 6**). However, the *ESR1* ortholog *PhESR1a* was markedly induced by day 4 (**Table 3**; **Supplementary Table 6**), consistent with its role in promoting shoot formation. This suggests that *PhESR1a* activation in petunia may occur through a *WIND*-independent pathway.

**Figure 3:**
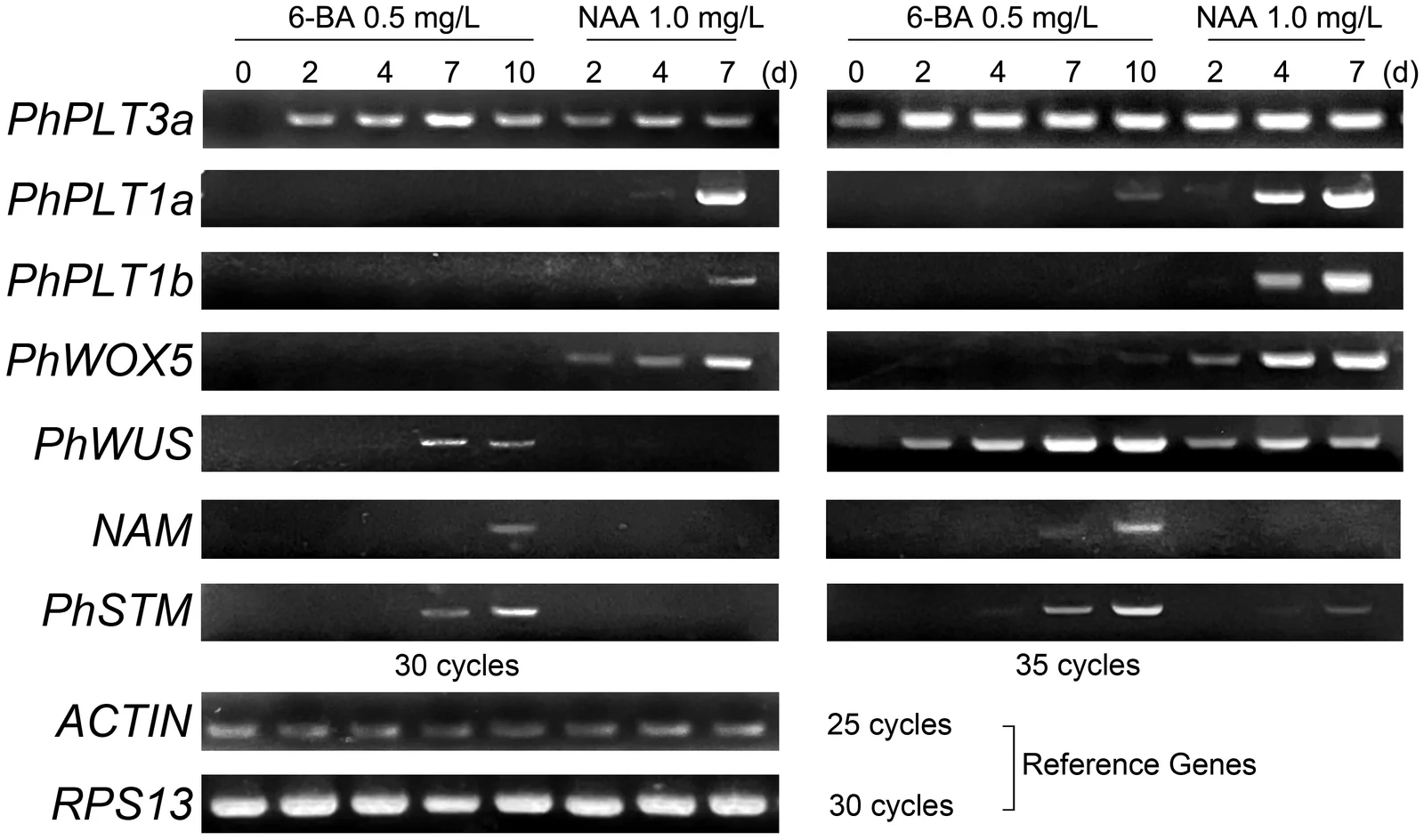
RT-qPCR validation of key root and shoot meristem gene expression during shoot regeneration on cytokinin-supplemented medium. *PhPLT3a*, *PhPLT1a*, *PhPLT1b*, *PhWOX5*, *PhWUS*, *NAM*, and *PhSTM* were analyzed by RT-PCR in leaf explants cultured on MS medium containing either 0.5 mg/L 6-BA or 1.0 mg/L NAA. Total RNA was extracted at the indicated time points (0, 2, 4, 7, 10 d for 6-BA treatment; 0, 2, 4, 7 d for NAA treatment). Amplification was performed for 30 or 35 cycles as indicated; *ACTIN* (25 cycles) and *RPS13* (30 cycles) served as reference genes. PCR products were separated on 1% agarose gels and visualized with GoldView nucleic acid stain. All experiments were repeated independently three times with similar results.

*PLT3*, *PLT5*, and *PLT7* are key regulators of lateral root development. In classic two-step regeneration, their auxin-induced expression promotes root quiescent center markers like *WOX5* and *PLT1/2*, conferring root primordium identity and pluripotency (Aida et al., 2004; Kareem et al., 2015; Xu et al., 2023). On cytokinin-supplemented medium, petunia *PLT3*/*7* orthologs were upregulated before day 4 (**Figure 3**; **Table 3**; **Supplementary Table 6**), consistent with a pro-regenerative role. In contrast, *PhPLT5* was transiently downregulated at 2 h and remained low (**Table 3**). Notably, the putative downstream targets *PhWOX5 and PhPLT1b* were not expressed, and *PhPLT1a* was detected at only minimal levels (**Figure 3**; **Supplementary Table 6**) by day 10. However, on medium with NAA 1.0 mg/L, *PhWOX5* was expressed by day 2, and *PhPLT1a/b* was expressed by day 4 (**Supplementary Table 6**; **Figure 3**). Furthermore, on cytokinin-supplemented medium, the root meristem marker *PhSHR* showed no significant change, and *PhSCR* was only induced by day 7 (**Table 3**, **S6**). Together, these results indicate that *PLT3/5/7*-mediated activation of *WOX5* and *PLT1*/*2* may require additional factors absent in this system, and the induced callus likely lacks distinct root primordium identity.

*Arabidopsis ERF113* (*RAP2.6L*) promotes shoot regeneration from callus (Che et al., 2006), while its close paralog *ERF115* is crucial for wound-induced callus formation and root stem cell repair (Heyman et al., 2016; Ikeuchi et al., 2017).The petunia orthologs *PhERF113a*/*b* were induced within 4 h and 1 d, respectively, and maintained high expression (**Table 3**; **Supplementary Table 6**), suggesting their functional role in petunia shoot regeneration.

LBD16/17/18/29 are key root formation regulators acting downstream of the IAA14–ARF7/19 module. They form a complex with bZIP59 to promote callus formation and pluripotency acquisition, essential for shoot regeneration in two-step systems (Fan et al., 2012; Liu et al., 2018; Xu et al., 2018b). During petunia regeneration on cytokinin-supplemented medium, significant expression changes were observed only for *PhLBD18a* (downregulated at 4 h) and *PhLBD18c* (upregulated at 7d). In contrast, *PhLBD16a*/*b*, *PhLBD18b* and *PhLBD29* showed no significant changes, with *PhLBD29* not detected under our experimental conditions (**Table 3**, **S6**). This suggests the LBD pathway may be dispensable for cytokinin-mediated petunia regeneration.

*Arabidopsis WOX13* and *WUS* mutually inhibit each other. *WOX13* promotes cell differentiation and expansion by inducing cell wall modifiers (e.g., *EXPANSIN*s, *MANNASE*7) and represses shoot meristem regulators (e.g., *WUS*, *STM*, *CUC1*, *ESR2*), thereby inhibiting shoot regeneration (Ogura et al., 2023). In contrast, its paralog *WOX14* promotes shoot regeneration (Wang et al., 2023). Petunia has two genes in this clade: *PhWOX13* (lacking a WOX-box) (Haecker et al., 2004) and *PhWOX14* (with a WOX-box). Transcriptome data showed *PhWOX13* was suppressed at 4 h, while *PhWOX14* was upregulated at day 7 (**Table 3**), indicating a role for this module in promoting regeneration in this system.

In *Arabidopsis* two-step regeneration, HD-ZIP III transcription factors (PHB, PHV, REV) form complexes with B-type ARRs to activate *WUS* (Zhang et al., 2017). During petunia regeneration, *PhPHB* was initially suppressed (2–4 h) but significantly activated by day 7 (**Table 3**), suggesting a conserved role in activating WUS.

Shoot apical meristem (SAM) genes *WUS*, *STM*, and *CUC*s are critical for SAM formation. Our transcriptome data showed *PhWUS* was significantly induced by day 4, while *PhSTM* and *PhNAM* were activated by day 7 (**Figure 3**; **Table 3**), coinciding with morphological signs of shoot initiation (**Figure 1**) and indicating SAM formation was underway by day 7.

Collectively, our transcriptomic profiling reveals a reconfigured regulatory network for *de novo* shoot regeneration in petunia. This network is characterized by the absence of key root-pluripotency markers *PhWOX5* and *PhPLT1b*, alongside distinct integration of factors such as *PhERF113*/*115* and the *PhWOX13*/*14* module, culminating in the conserved upregulation of shoot meristem genes *WUS* and *STM*.

### Functional analysis of petunia *WIND*s during adventitious shoot regeneration from leaf discs

Transcriptomic data indicated that during adventitious shoot regeneration on a cytokinin-supplemented medium, the expression of *WIND* genes was primarily downregulated or did not reach a significant level of induction (log2FC ≥ 1, *P_adjust_* ≤ 0.05; **Table 3**). This contrasts with the typical wound-induced *WIND* expression that promotes callus formation and shoot regeneration in *Arabidopsis*. We hypothesized that either cytokinin in the medium suppressed their expression, or they were transiently activated too rapidly, with their expression peak occurring before our first time point at 2 hours.

**Figure 4:**
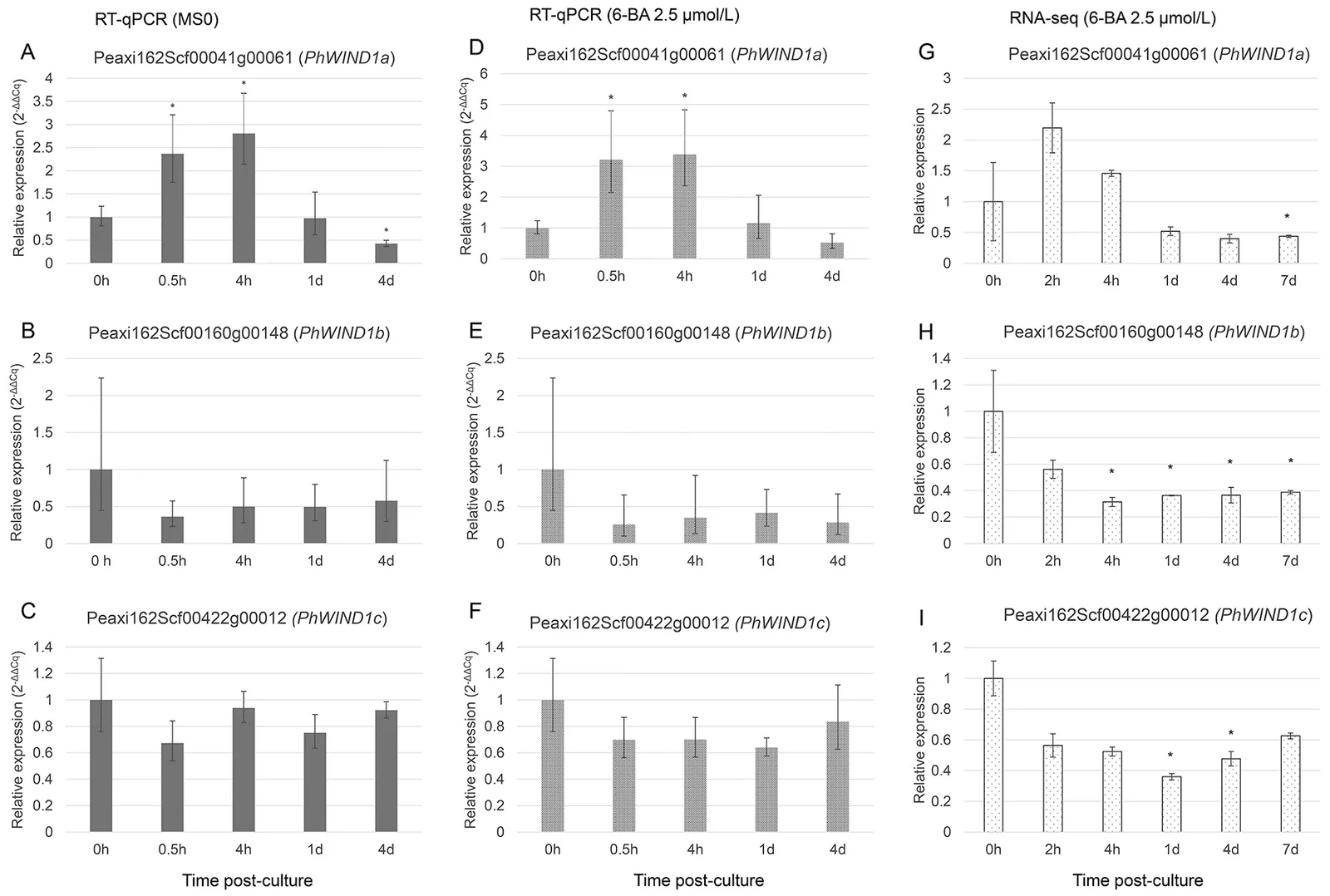
Expression dynamics of *PhWIND* genes in leaf explants cultured on hormone-free MS medium or MS medium supplemented with 2.5 μmol/L 6-BA. **(A–F)** RT-qPCR validation of *PhWIND1a*, *PhWIND1b*, and *PhWIND1c* transcript levels. Samples were collected at 0 h, 0.5 h, 4 h, 1 d, and 4 d after culture. Relative expression was calculated using the 2^-ΔΔCt^ method; statistical significance was assessed on 2^-ΔCt^ values. Error bars represent 2^(−ΔΔCt ± SD)^ (n = 3 biological replicates). Asterisks indicate significant differences between each time point and the 0-h control (*P* < 0.05, Student’s t-test). **(G–I)** Transcript levels of *PhWIND* genes determined by RNA-seq (TPM). Asterisks indicate adjusted P < 0.05 for comparisons versus the 0-h control (Benjamini–Hochberg procedure for FDR control).

To investigate this, we performed RT-qPCR analysis. The results confirmed the trends observed in the RNA-seq data. Among the three *WIND* genes, only *PhWIND1a* showed any upregulation. This induction was not suppressed by the addition of 6-BA, and the magnitude of upregulation was similar at 0.5 h and 4 h. However, this induction was transient, lasting less than 24 hours (**Figure 4**).

**Figure 5:**
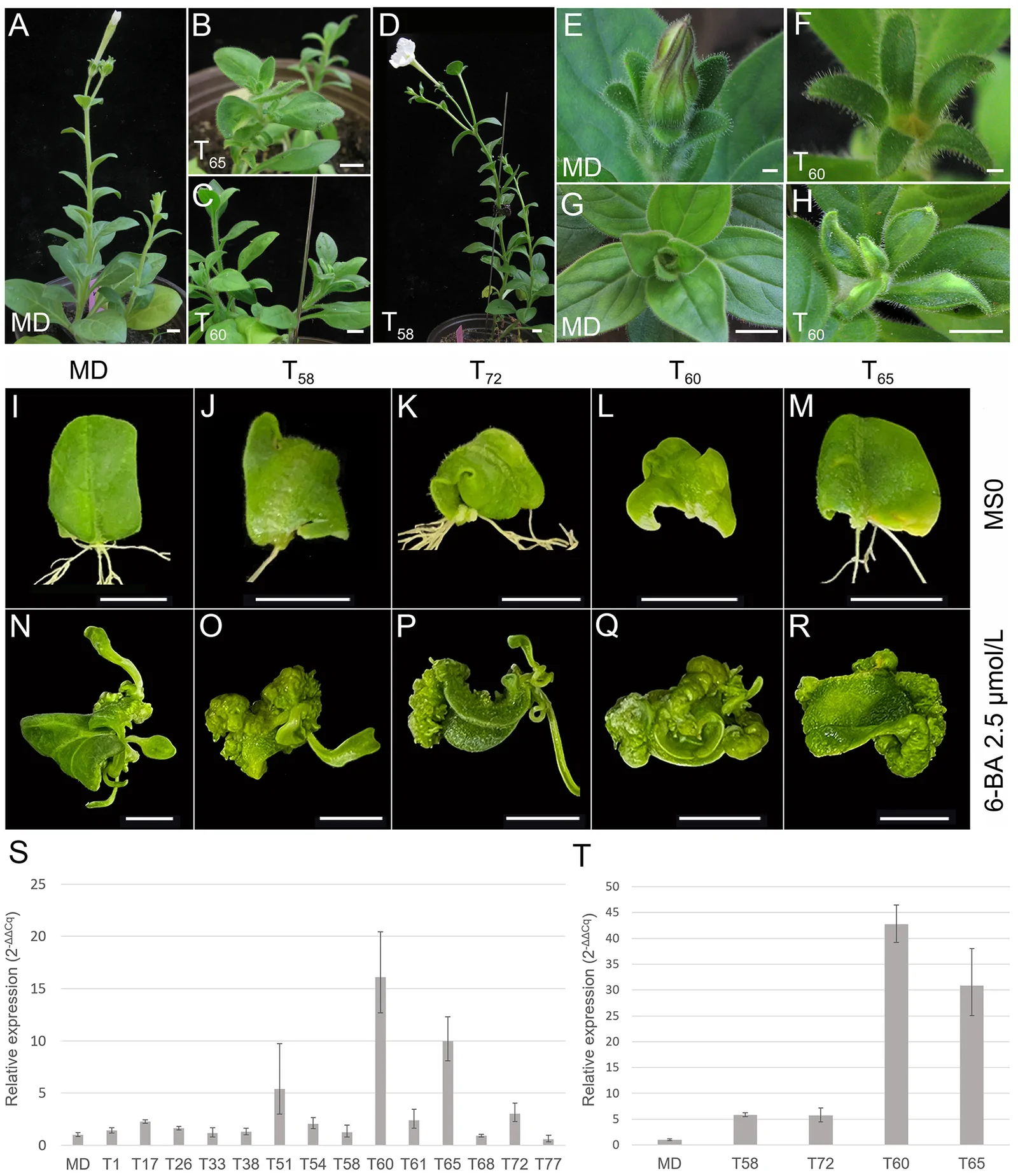
*PhWIND1* overexpression fails to enhance callus proliferation or adventitious shoot regeneration from petunia leaf explants. **(A–D)** Phenotypes of wild-type MD **(A)**, *PhWIND1a*-OE lines **(B, C)**, and a transgenic line with comparable expression expression **(D)**. **(E–F)** *PhWIND1a* overexpression induced flower bud abortion. **(G, H)** Abnormal leaf development in *PhWIND1a*-OE plants. **(I–M)** Leaf explants from *PhWIND1a* transgenic lines did not promote callus formation on hormone-free medium. **(N–R)** Leaf explants from *PhWIND1a* transgenic lines did not promote callus or shoot formation on cytokinin-supplemented medium. **(S)** Relative *PhWIND1a* expression levels in transgenic leaves. **(T)** *PhWIND1a* expression in callus induced on cytokinin-supplemented medium. Scale bars: (**A–D, G–R)** = 1 cm; **(E–F)** = 0.1 cm.

Given the differing expression dynamics of *WIND* genes between petunia leaf discs and wounded *Arabidopsis* explants, we sought to determine whether *PhWIND1a* function is conserved. In *Arabidopsis*, constitutive expression of *WIND1* is sufficient to induce callus formation and adventitious shoot regeneration, even on hormone-free medium (Iwase et al., 2011). To test this in petunia, we generated plants overexpressing *PhWIND1a* under the control of the 35S promoter (2×35S:*PhWIND1a*). During the transformation and selection process, we actively monitored but did not observe any spontaneous callus formation that is typical of *Arabidopsis* overexpressing WIND1. From 18 BASTA-resistant lines, PCR confirmed transgene integration. RT-qPCR analysis of 14 lines identified two lines with 10- to 16-fold higher *PhWIND1a* expression than the wild-type, which we designated as *WIND1a*-OE lines; most of the remaining lines showed expression levels within 3-fold of the wild-type level, indicating comparable expression (**Figure 5S**).

The *WIND1a*-OE plants exhibited growth retardation, characterized by reduced plant height, smaller leaves, and bud abortion (**Figures 5A–F**). Additionally, some shoot apices produced curved young leaves with white margins (**Figures 5G, H**). These phenotypes indicate that constitutive *PhWIND1a* expression severely disrupts normal development in petunia.

When cultured on hormone-free MS medium, leaf discs from two independent *WIND1a*-OE lines and two lines with only modestly increased expression did not exhibit enhanced callus formation (**Figures 5I–M**; **Supplementary Figure 2**). Furthermore, on regeneration medium containing 2.5 μmol/L 6-BA, all transgenic lines showed no significant difference from the wild-type in callus formation frequency, callus size, or shoot number (**Figures 5N–R**; **Supplementary Figure 2**). Notably, the expression level of *PhWIND1a* in the callus induced on this cytokinin- supplemented medium was comparable to that in the original leaves (**Figures 5S, T**). These results demonstrate that, under our experimental conditions, *PhWIND1a* overexpression does not enhance *in vitro* regeneration in petunia.

## Discussion

### Transcriptomic profiles during callus and shoot formation from petunia leaf discs on cytokinin-supplemented medium

The regeneration of adventitious shoots from petunia leaf discs on cytokinin-supplemented MS medium proceeds through callus formation, representing an indirect organogenesis pathway that shares similarities with the classic two-step regeneration system in *Arabidopsis*. To elucidate the molecular dynamics underlying this process, we conducted RNA-seq analysis on explants collected at various time points, ranging from hours to days after culture. Our results uncover both conserved and distinct features compared with other species and regeneration protocols.

The transcriptional program delineates several distinct yet interconnected phases during *de novo* shoot regeneration: an initial wound stress response, activation of macromolecule biosynthesis, onset of cell division, and eventual establishment of shoot apical meristems. This phased progression exhibits remarkable conservation across plant species, suggesting fundamental principles that govern *in vitro* regeneration.

The first cluster of differentially expressed genes (DEGs), peaking at 2 hours, was enriched for processes related to membrane and cell wall repair, wound signaling, jasmonic acid metabolism, and calcium ion binding. This indicates that explants rapidly activate damage response mechanisms, consistent with the findings that wound signaling serves as a crucial initial trigger for plant regeneration (Iwase et al., 2015; Ikeuchi et al., 2016, Ikeuchi et al., 2020; Xu and Yang, 2025). The early activation of calcium signaling and jasmonic acid metabolism aligns with their established roles as key secondary messengers in wound response and cellular reprogramming (Zhang et al., 2019, Zhang et al., 2023; Li et al., 2025), suggesting a universal alarm-and-repair phase preceding cell fate redirection.

The second cluster, peaking at 4 hours, showed strong enrichment for primary metabolic processes, translation, ribosome biogenesis, and energy metabolism, indicating rapid reprogramming toward biosynthetic activation. The coordinated upregulation of glycolytic and TCA cycle genes points to enhanced energy production to fuel subsequent regenerative events. This metabolic activation likely provides the essential resources for proliferative growth, as evidenced by the temporal coupling between these biosynthetic genes and DNA replication markers in Cluster 3. This proliferative phase corresponds to the formation of callus tissue, which serves as an intermediate progenitor pool for organogenesis.

This pattern of metabolic and proliferative activation is not unique to our system but represents a conserved regenerative strategy. Enhanced energy metabolism and protein synthesis preceding cell cycle re-entry have also been observed during wound-induced callus formation in *Arabidopsis* hypocotyls and in protoplast culture (Chupeau et al., 2013; Ikeuchi et al., 2017). In tobacco protoplasts, RNA, DNA, and protein synthesis were activated prior to the first cell division observed at 40 hours of culture (Zelcer and Galun, 1976). Similarly, cell division genes were upregulated around 48 hours during *Arabidopsis* callus initiation (Xu et al., 2012). A sequential activation of protein biosynthesis, cell division, and shoot development has been documented during direct shoot regeneration in *Torenia fournieri* (Morinaka et al., 2021), and dynamic regulation of stress response, metabolic, and mitotic pathways has been reported at key developmental switches during somatic embryogenesis in *Coffea arabica* (Awada et al., 2023).

The recovery and enhancement of photosynthetic gene expression (Cluster 4) after an initial decline may support the substantial energy demands of the later morphogenetic stages. Dynamic expression of photosynthetic genes during *in vitro* culture has been observed in various species and protocols, including *Arabidopsis* (Che et al., 2002, Che et al., 2006), tomato (Song et al., 2023), and *Coffea arabica* (Awada et al., 2023), underscoring the importance of flexible energy metabolism during *de novo* organogenesis.

However, the transient suppression of auxin signaling genes (Cluster 5) in our system highlights the complex hormonal rebalancing required for callus formation in a cytokinin-dominated environment. This contrasts with the auxin-rich conditions typically used for callus induction in the *Arabidopsis* two-step regeneration system (Motte et al., 2014). Furthermore, the coordinated downregulation of ethylene response pathways (Cluster 6) likely removes a key inhibitory signal for shoot regeneration, consistent with reports that ethylene signaling acts as a general repressor of regeneration across multiple species (Biddington, 1992; Pua and Lee, 1995; Hu et al., 2006). These findings suggest that the molecular nature of callus induced in a cytokinin-supplemented medium may differ from that of auxin-induced callus.

In summary, the dynamic transcriptional changes we observed outline a coordinated genetic program underpinning adventitious shoot regeneration. The sequential activation from wound healing to metabolic activation and cell division constitutes a critical preparatory phase for organogenesis. This core program appears to be a common requirement for successful *de novo* organogenesis across diverse *in vitro* culture systems and regeneration protocols.

### Cytokinin-auxin interaction during shoot organogenesis on cytokinin-supplemented medium

The molecular orchestration of *de novo* organogenesis *in vitro* provides a powerful system for understanding cell fate reprogramming. Our transcriptomic analysis of petunia leaf explants cultured on a cytokinin-supplemented medium reveals a dynamic and sequential interplay between cytokinin and auxin pathways, critical for driving the one-step regeneration of adventitious shoots.

Our data demonstrate that the explants mounted a canonical cytokinin response upon culture. The widespread upregulation of A-type Response Regulator (RR) genes is a hallmark of activated cytokinin signaling (Hwang et al., 2012). Concurrently, the metabolic landscape of CK was reshaped to modulate its homeostasis, as evidenced by the induction of *CKX* genes, which promote CK degradation, and the general suppression of *LOG* genes, which are responsible for the final step of active CK synthesis (Kurakawa et al., 2007). This coordinated transcriptional reprogramming indicates that the exogenous 6-BA was perceived and transduced into a robust signaling cascade, establishing a high-cytokinin state within the explant—a prerequisite for shoot progenitor fate determination (Gordon et al., 2007).

A pivotal finding of this study is how this high-cytokinin environment profoundly influenced the endogenous auxin pathway in the absence of exogenous auxin. The initial suppression of the auxin pathway—characterized by the downregulation of the key biosynthetic gene *YUC*, early-responsive *Aux*/*IAA* genes, and polar auxin transporters like *PIN*s within the first day—suggests a rapid dampening of auxin signaling capacity. This early repression may be a critical step to release cells from their original, differentiated state and to suppress root-promoting pathways, effectively clearing the developmental slate for subsequent shoot formation (Meng et al., 2017).

However, the narrative of auxin suppression is not absolute. The upregulation of *PhTAA1a* before 4 hours and *PhTAR2* at 7 days implies that auxin biosynthesis may be enhanced in specific cell populations. The subsequent upregulation of specific components from day 4 onwards, including *PhIAA6b*/*27c*, *PhPIN1b/5a/6*, and *PhLAX2a/b*, strongly suggests a spatially and temporally restricted reactivation of auxin signaling and transport. This late-phase induction likely signifies the establishment of localized auxin response maxima and polar auxin transport channels, which are indispensable for the patterning and initiation of meristematic centers (Reinhardt et al., 2003; Su et al., 2011; Cheng et al., 2013).

Within this context, the specific and sustained upregulation of *PhARF5* emerges as a potentially critical event. In *Arabidopsis*, *ARF5*/*MONOPTEROS* is a master regulator of shoot stem cell maintenance and differentiation, partly achieved through negatively regulating *ARR7*, *ARR15*, and *ESR1* (Zhao et al., 2010; Luo et al., 2018; Wójcikowska et al., 2023). Its functional necessity is underscored by the finding that loss-of-function mutations in *ARF5* inhibit both callus formation and *de novo* shoot organogenesis (Zhang et al., 2021b), while an irrepressible variant is sufficient to promote shoot regeneration from callus (Ckurshumova et al., 2014; Gonzalez et al., 2021). Notably, genome-wide transcriptome analyses identified *ARF5* as the sole ARF family member upregulated during the early stage (within 96 hours) of callus initiation across multiple *Arabidopsis* organs (Xu et al., 2012). Furthermore, during somatic embryogenesis in both *Arabidopsis* and *Coffea arabica*, *ARF5* is the most highly upregulated ARF in embryogenic cells (Wójcikowska and Gaj, 2017; Awada et al., 2023). Based on this conserved role of ARF5 in other systems, together with its specific upregulation in our dataset, we hypothesize that *PhARF5* may function as a central integrator in petunia one step regeneration. However, we acknowledge that this hypothesis currently rests on correlative expression data and will require direct functional validation in future work.

**Figure 6:**
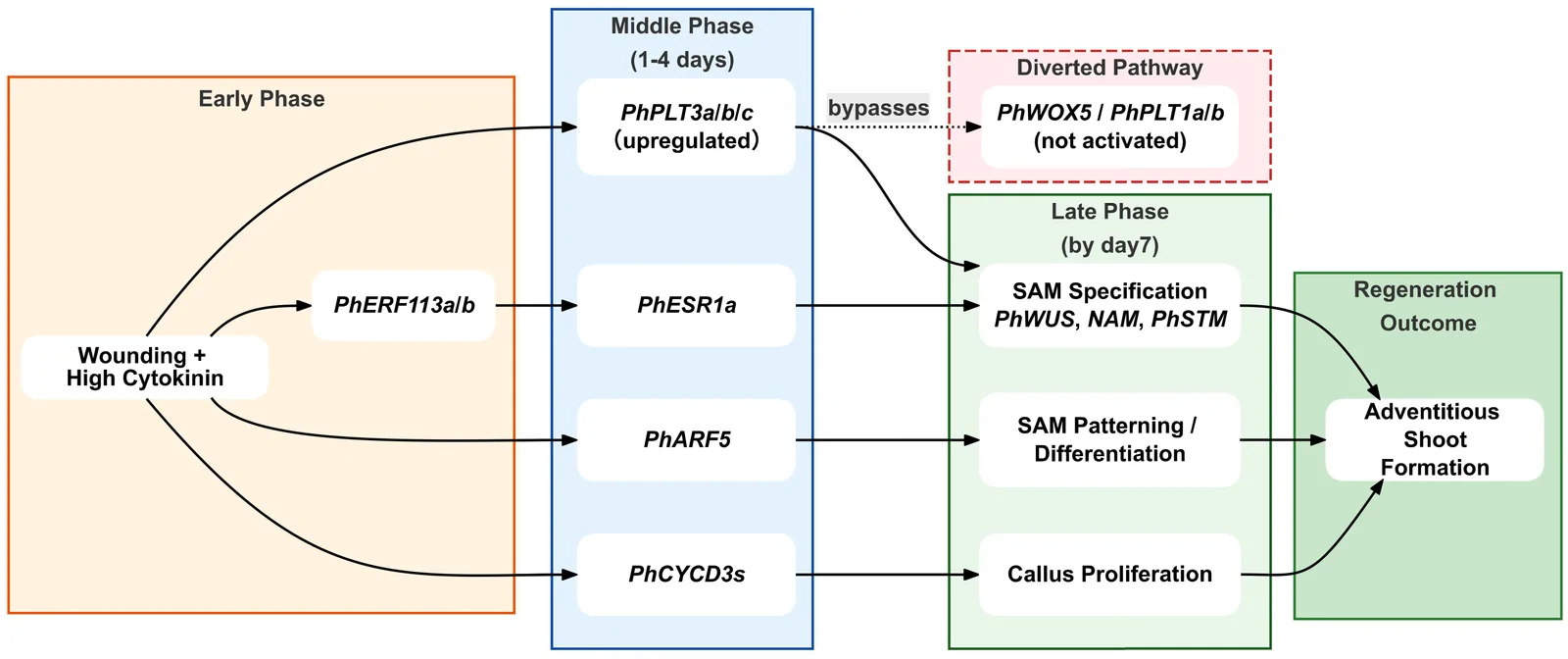
A simplified model for *de novo* shoot regeneration from petunia leaf explants on cytokinin-supplemented medium. Wounding and high cytokinin signaling activate a temporally coordinated transcriptional cascade. In the early phase (hours), *PhERF113a/b* are rapidly induced. During the middle phase (days 1–4), multiple downstream events occur in parallel: cytokinin promotes callus proliferation via *PhCYCD3s*, induces the shoot-promoting factor *PhESR1a*, reprograms the auxin pathway to upregulate *PhARF5*, and upregulates *PhPLT3a/b/c*. Notably, although *PhPLT3a/b/c* are upregulated, their canonical downstream root program targets (*PhWOX5*, *PhPLT1a/b*) remain inactive (dashed arrow). Instead, in this system they likely contribute to activating SAM genes (*PhWUS*, *NAM*, *PhSTM*), a function consistent with their role in *Arabidopsis*. In the late phase (by day 7), the convergence of callus proliferation, SAM specification, and SAM patterning/differentiation ultimately leads to adventitious shoot formation.

Building on this, we propose a working model for *de novo* shoot regeneration from petunia leaf explants on cytokinin-supplemented medium (**Figure 6**). In this model, wounding and high-cytokinin signals coordinately drive callus proliferation (potentially via *PhCYCD3*s), induce SAM genes (e.g., *PhWUS*), and reprogram the auxin pathway to upregulate *PhARF5*. We postulate that *PhARF5* may act as a key integrator, synergizing with cytokinin to ensure SAM patterning and differentiation, ultimately leading to adventitious shoot formation. Future experimental validation will be essential to test this hypothesis.

### A root-meristem-independent pathway for shoot regeneration

The molecular roadmap of *de novo* shoot regeneration has been extensively studied in model systems such as *Arabidopsis thaliana*, which typically requires a two-step procedure involving a CIM followed by a SIM. Our transcriptomic analysis of petunia leaf explants regenerating shoots directly on a cytokinin-supplemented medium uncovers both conserved and distinct aspects of the underlying transcriptional logic, pointing to alternative paths to the same developmental endpoint.

A key distinction lies in the absence of a canonical root-meristem signature during petunia shoot organogenesis. In the *Arabidopsis* two-step method, the auxin-rich CIM promotes the formation of a progenitor cell population with root meristem characteristics, driven by the induction of *PLT3/5/7* and subsequent activation of *PLT1/2* and *WOX5* (Kareem et al., 2015; Xu et al., 2023; Chen et al., 2024). Our data show that while the petunia homologs *PhPLT3a/b/c* were upregulated by day 4, their putative downstream targets, *PhPLT1a/b* and *PhWOX5*, remained silent. This uncoupling of the *PLT* cascade indicates that the activation of *PLT1/2* and *WOX5* is not an obligatory step for shoot regeneration. Consistently, homologs of other root stem cell regulators—including *LBD*s, *WOX11/12*, *SHR*, and *JACKDAW (*a direct target of *PLT1/2* required for pluripotency acquisition; Zhai et al., 2023)—were not activated (**Supplementary Table 6**), further reinforcing that auxin-dependent LRP-like structures were not induced in our regeneration system.

Why then do petunia leaf explants regenerate shoots efficiently despite the absence of canonical LRP markers? We consider two non-mutually exclusive possibilities. First, this may reflect species-specific differences in regeneration competence. In *Arabidopsis*, the two-step system appears to require an auxin-induced LRP-like intermediate to establish pluripotency before cytokinin can act. In contrast, other species, including *Torenia fournieri* (Morinaka et al., 2021, Morinaka et al., 2023) and petunia, appear capable of directly perceiving and responding to cytokinin signals without first acquiring an LRP-like identity—possibly due to differences in the basal expression of regeneration-associated genes or in their responsiveness to wounding. Second, the molecular definition of pluripotency itself warrants reconsideration. While *WOX5*, *PLT1/2* and other LRP markers are tightly associated with pluripotency in *Arabidopsis* callus, it is not established that their expression is the exclusive hallmark of a pluripotent state in all species or culture systems. The uncoupling of the *PLT3/5/7* cascade from its downstream LRP targets suggests that alternative gene networks may be sufficient to confer regeneration competence.

This interpretation is consistent with observations in *Torenia fournieri*, where direct shoot regeneration from stem epidermal cells on a cytokinin-containing medium occurs without activation of *WOX5*, *PLT1*/*2*, and *LBD16* (Morinaka et al., 2021, Morinaka et al., 2023). Wound-induced callus from *Arabidopsis* hypocotyls similarly lacks root meristem markers (Iwase et al., 2011; Ikeuchi et al., 2013) and *WOX5* is not detected in callus on hormone-free medium (Bustillo-Avendaño et al., 2018). As wound-induced callus formation depends on wound-induced cytokinin rather than auxin (Ikeuchi et al., 2017), callus formed in auxin-free environments may generally lack LRP characteristics.

Further highlighting the unique nature of this one-step regeneration, we observed a distinct configuration of the WOX gene regulatory network. In *Arabidopsis*, *WOX13*—which is upregulated after wounding (Ikeuchi et al., 2017)—represses shoot regeneration by inhibiting meristem genes like *WUS* (Ogura et al., 2023). In our system, *PhWOX13* was rapidly suppressed, thereby potentially releasing this brake on shoot promotion. Concurrently, *PhWOX14*, a homolog of the shoot-regeneration-promoting *AtWOX14* (Wang et al., 2023), was upregulated. This coordinated suppression of a repressor and induction of a promoter likely creates a permissive transcriptional environment for shoot fate specification.

Furthermore, classic shoot meristem regulators were activated with expected kinetics: the *ERF113/115* homologs were induced early, potentially in response to wounding and cytokinin signaling to promote cell proliferation (Che et al., 2006; Heyman et al., 2016; Yang et al., 2018; Lakehal et al., 2020). This was followed by the subsequent activation of *PhESR1a*, *PhWUS*, and *PhPHB*, and finally *PhSTM* and *PhNAM*, marking the establishment of a functional shoot apical meristem (Zhang et al., 2017). The observed kinetics alighn with a yeast-one-hybrid analysis that pinpointed *PLT3* and *ESR1* as hub genes in a reprogramming network (Ikeuchi et al., 2018).

We acknowledge that the absence of a time-matched hormone-free control limits our ability to formally distinguish cytokinin-specific effects from generic wound or culture-progression responses. However, we previously observed that hormone-free medium can induce SAM gene expression in petunia leaf explants, but at levels substantially lower than those on cytokinin-supplemented medium, and without leading to shoot formation (Guo et al., 2021). This suggests that while wounding and culture conditions can elicit a basal transcriptional response, cytokinin is required to amplify and sustain this program to achieve organogenesis.

In conclusion, although root meristem genes such as *WOX5*, *PLT1/2*, and *SHR* are considered key transcriptional regulators that confer pluripotency to callus cells during adventitious shoot regeneration in *Arabidopsis* (Ikeuchi et al., 2019; Sugimoto et al., 2019; Shin et al., 2020), our results demonstrate that shoot regeneration in petunia on cytokinin-supplemented medium orchestrates cell fate transition through a streamlined pathway that bypasses the root-meristem-like phase. Cytokinin signaling, potentially aided by specific *ERF* and *WOX* family members (e.g., *PhPLT3a/b/c*, *PhERF113a/b*, and the *PhWOX13/14* module), directly promotes the establishment of shoot meristem identity through *ESR1/2* and *WUS*, without activating the root-based pluripotency network involving *PLT1/2* and *WOX5* (**Figure 6**). This comparative analysis underscores the plasticity of plant regeneration pathways, showing that different species and protocols can recruit distinct yet equally effective transcriptional modules to achieve organogenesis.

### Divergent role of *WIND* genes in regeneration

Our data point to a divergent role for *WIND* genes in petunia regeneration compared to the established paradigm in *Arabidopsis*. In *Arabidopsis*, *WIND* genes are wound-induced master regulators that promote cell dedifferentiation and callus formation, operating in part through the activation of *ESR1* (Iwase et al., 2011, Iwase et al., 2017). However, the necessity and potency of this *WIND*-*ESR1* regulatory module are highly context-dependent. This is exemplified by studies in tomato: while ectopic expression of *AtWIND1* promotes callus formation in intact plants on hormone-free medium (Iwase et al., 2014), it fails to enhance regeneration from cotyledon and hypocotyl explants on hormone-containing media (Iwase et al., 2015). This distinction highlights that the regenerative pathway in a structured tissue explant system may bypass or override the *WIND* pathway. Most decisively, a *wind* quadruple mutant in *Arabidopsis* did not impair callus formation, suggesting redundant regulators (Iwase et al., 2021), while a recent study in tomato demonstrated clear gain- and loss-of-function phenotypes for *SlWIND1* (Yang et al., 2024). Together, this paints a picture of complex functional conservation.

In this landscape, petunia presents a compelling case in which the canonical *WIND*-*ESR1* axis is broken. Our transcriptomic and functional analyses argue against an essential role for *WIND* homologs in this system. The genes were not sustainably upregulated, and critically, *PhWIND1a* overexpression—while disruptive to development—failed to enhance regeneration. We acknowledge that the constitutive overexpression approach used here has inherent limitations, as the severe pleiotropic phenotypes (dwarfism, flower bud abortion, and leaf malformation) could potentially mask a genuine regenerative function. Nevertheless, it is worth noting that the *PhWIND* genes already exhibit relatively high basal expression in petunia leaves (average TPM at 0 h of 103.7 ± 46.5, 35.8 ± 7.9, and 248.4 ± 19.8 for *PhWIND1a*, *PhWIND1b*, and *PhWIND1c*, respectively). The fact that strong constitutive overexpression of *PhWIND1a* led to severe developmental abnormalities without enhancing regeneration suggests that simply increasing the transcript level of this gene is not sufficient to promote shoot organogenesis in this system, and may even be detrimental.

The most telling evidence is the strong induction of the key shoot-promoting factor *PhESR1a* despite the lack of sustained wound-inducible activation of the *WIND* homologs, indicating that *PhESR1a* activation occurs through a *WIND*-independent pathway.

Taken together, our findings suggest that the petunia regeneration program on cytokinin-supplemented medium bypasses the canonical *WIND*-dependent pathway. Instead, it appears to have co-opted a more direct route, potentially via cytokinin signaling itself, to activate the core regulators of organogenesis. Given the limitations of constitutive overexpression, we recognize that inducible or tissue specific expression systems would be more suitable for precisely dissecting the function of WIND genes in future studies.

Our decision to prioritize *PhWIND1a* for functional analysis was based on its established role as a wound-induced master regulator in *Arabidopsis* (Iwase et al., 2011) and its unique transient upregulation upon wounding among the three petunia WIND homologs. In contrast, the role of *PhARF5* in this system, while strongly suggested by its sustained upregulation and the conserved function of *ARF5* in *Arabidopsis*, remains correlative and will require direct experimental validation. Loss-of-function and gain-of-function studies of *PhARF5* are currently being planned as the next logical step to test its functional significance in the one-step regeneration pathway.

## Data Availability

The datasets presented in this study can be found in online repositories. The names of the repository/repositories and accession number(s) can be found in the article/**Supplementary Material**.

## Supplementary material

Supplementary Figure 1 Validation of RNA-seq-derived expression profiles by RT-qPCR

Supplementary Figure 2 Comparison of callus formation and shoot regeneration between *PhWIND1a* transgenic lines and wild-type (MD)

Supplementary Document 1 Phylogenetic trees of regeneration-associated genes

Supplementary Table 1 PCR primers used in this study

Supplementary Table 2 List of genes included in each of the six clusters

Supplementary Table 3 Nomenclature and expression levels of petunia genes implicated in cytokinin metabolism and signaling

Supplementary Table 4 Nomenclature and expression levels of petunia genes implicated in auxin metabolism and signaling

Supplementary Table 5 List of petunia orthologs of the *Arabidopsis* regeneration-associated genes

Supplementary Table 6 Expression levels of petunia regeneration-associated genes
