# Supplementary material for: Cytokinin-supplemented medium drives de novo shoot regeneration in petunia (Petunia hybrida) by bypassing the root-meristem program
Source: Front Plant Sci. 2026 Aug 28;17:1918195. doi: 10.3389/fpls.2026.1918195 (PMC13562028; doi:10.3389/fpls.2026.1918195)

Phylogenetic trees of regeneration regulators and genes involved in cytokinin and auxin metabolism and signaling

Directory of Phylogenetic Trees

Gene family	Slide No.		Gene family	Slide No.		Gene family	Slide No.
PLT	5		E2Fa	18		CEP	31
WIND	6		B3	19		FAD-BD	32
ERF113, 114, 115	7		NF-YB	20		KCS1	33
ESRs	8		RKD4	21		MSL1	34
CRF	9		GRF5	22		PME2	35
JKD	10		WRKY23	23		XTH	36
GRAS	11		bHLH41	24		CLF and SWN	37
HD-ZIPⅢ	12		bZip59	25		FIE	38
LOB	13		CYCD3	26		ATXR2	39
WOX	14		KMDs	27		JMJ30	40
NAC	15		CA2	28		HAG1/GCN5	41
KNOX	16		FLA1	29		MET1	42
Dof	17		SIZ1	30		IPT	44

Directory of Phylogenetic Trees

Gene family	Slide No.		Gene family	Slide No.		Gene family	Slide No.
LOG	45		ARF	59			
CKX	46		GH3	60			
HK	47						
HP	48						
Type-A RR	49						
Type-B RR	50						
TA	52						
YUCCA	53						
DAO	54						
ATL	55						
AEC	56						
TIR1	57						
Aux/IAA	58						

Phylogenetic trees of plant regeneration regulators

PLT genes

(**CSRRs: PLT1, 2, 3, 5, 7**)

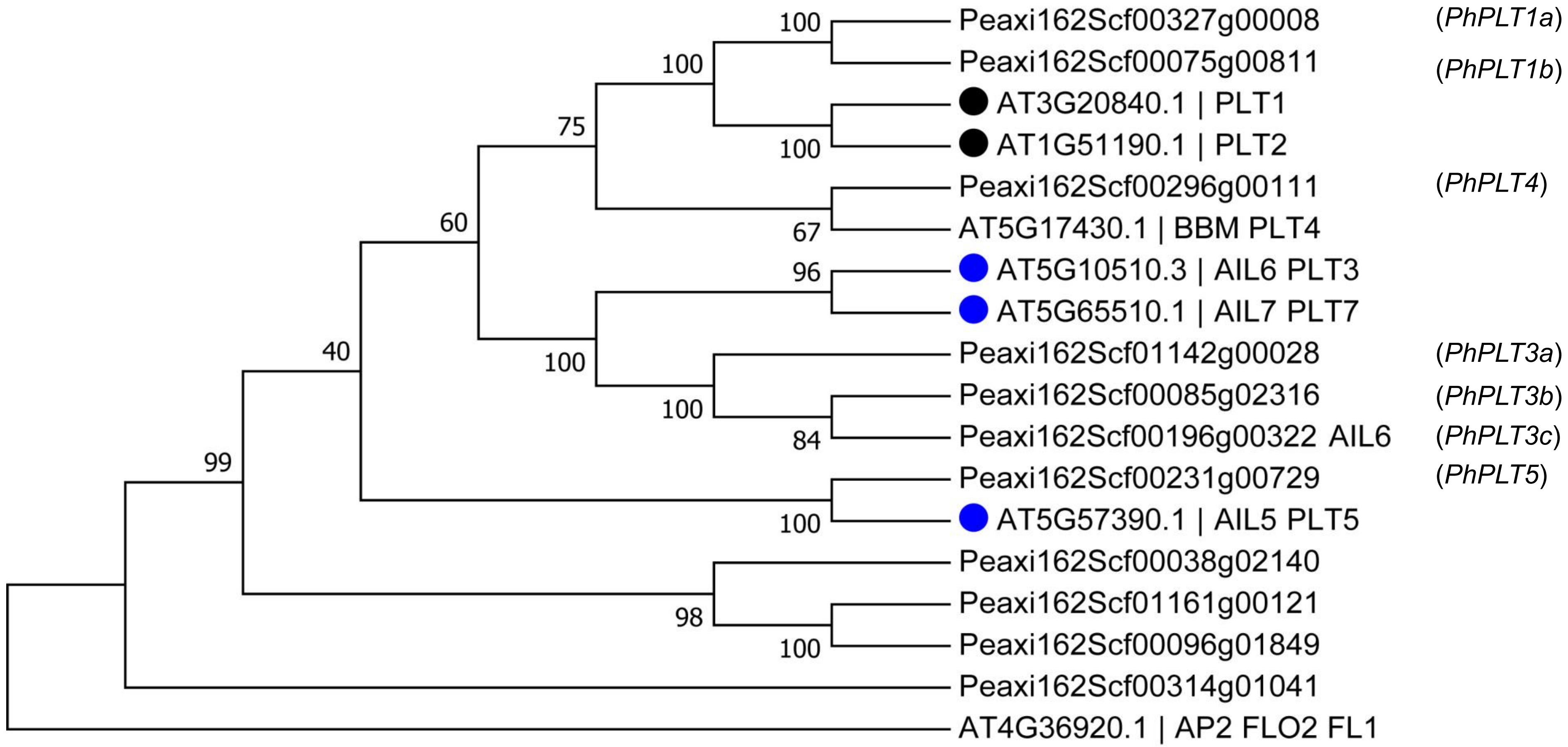

WINDs

(**CSRRs: WIND1, 2, 3, 4**)

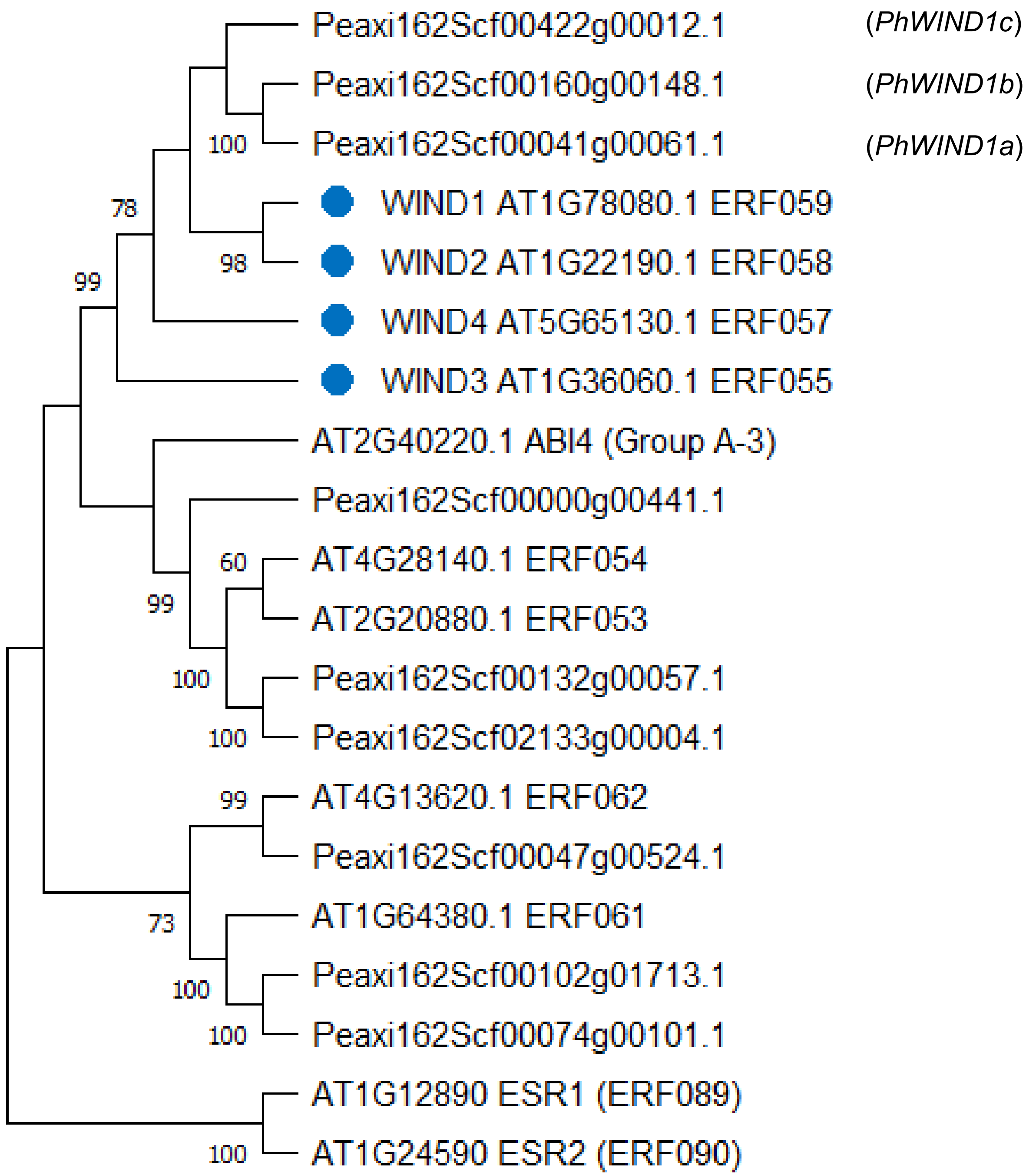

ERFs

(CSRR: ERF113,114,115)

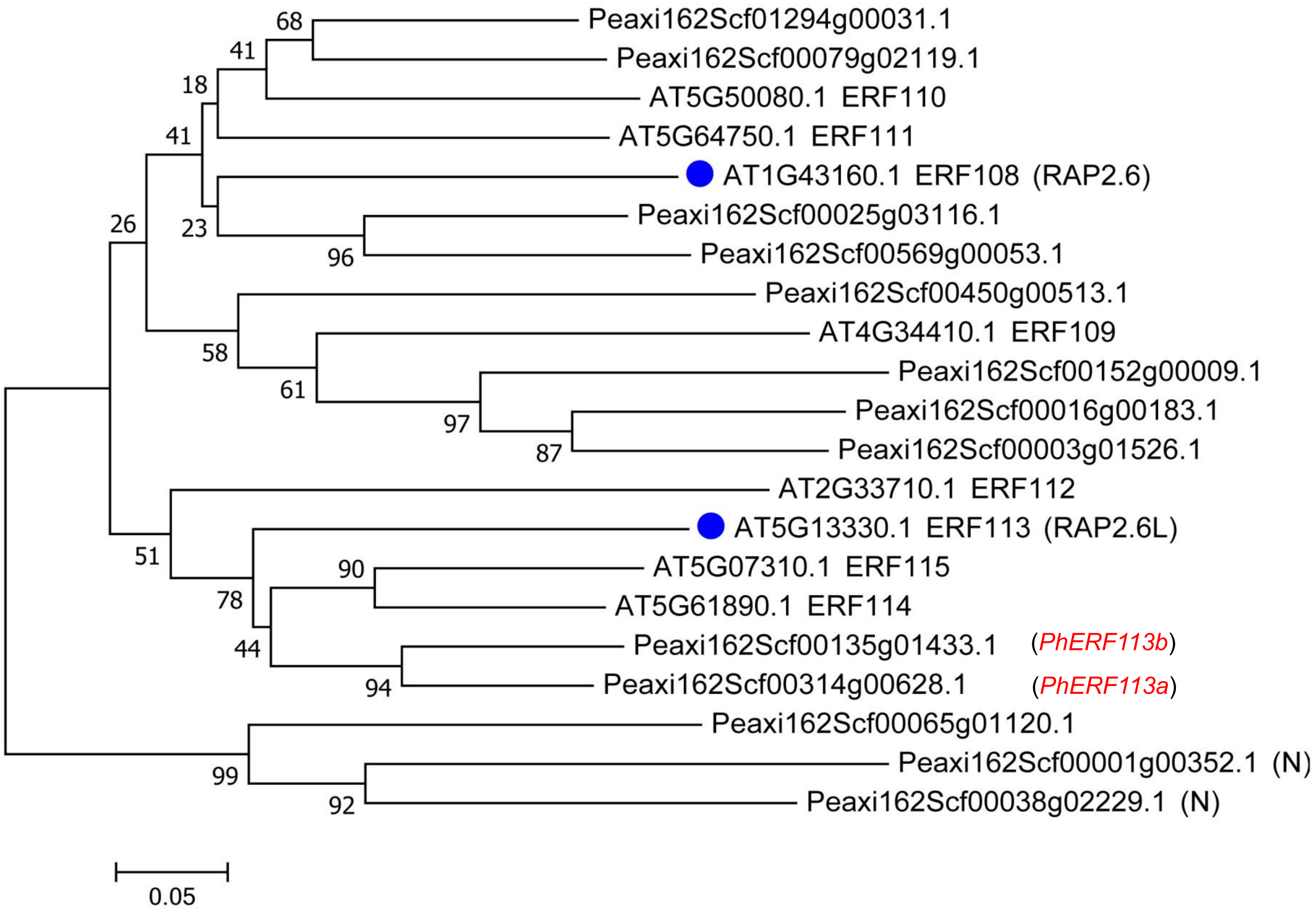

ESRs

(**CSRR: ESR1, 2**)

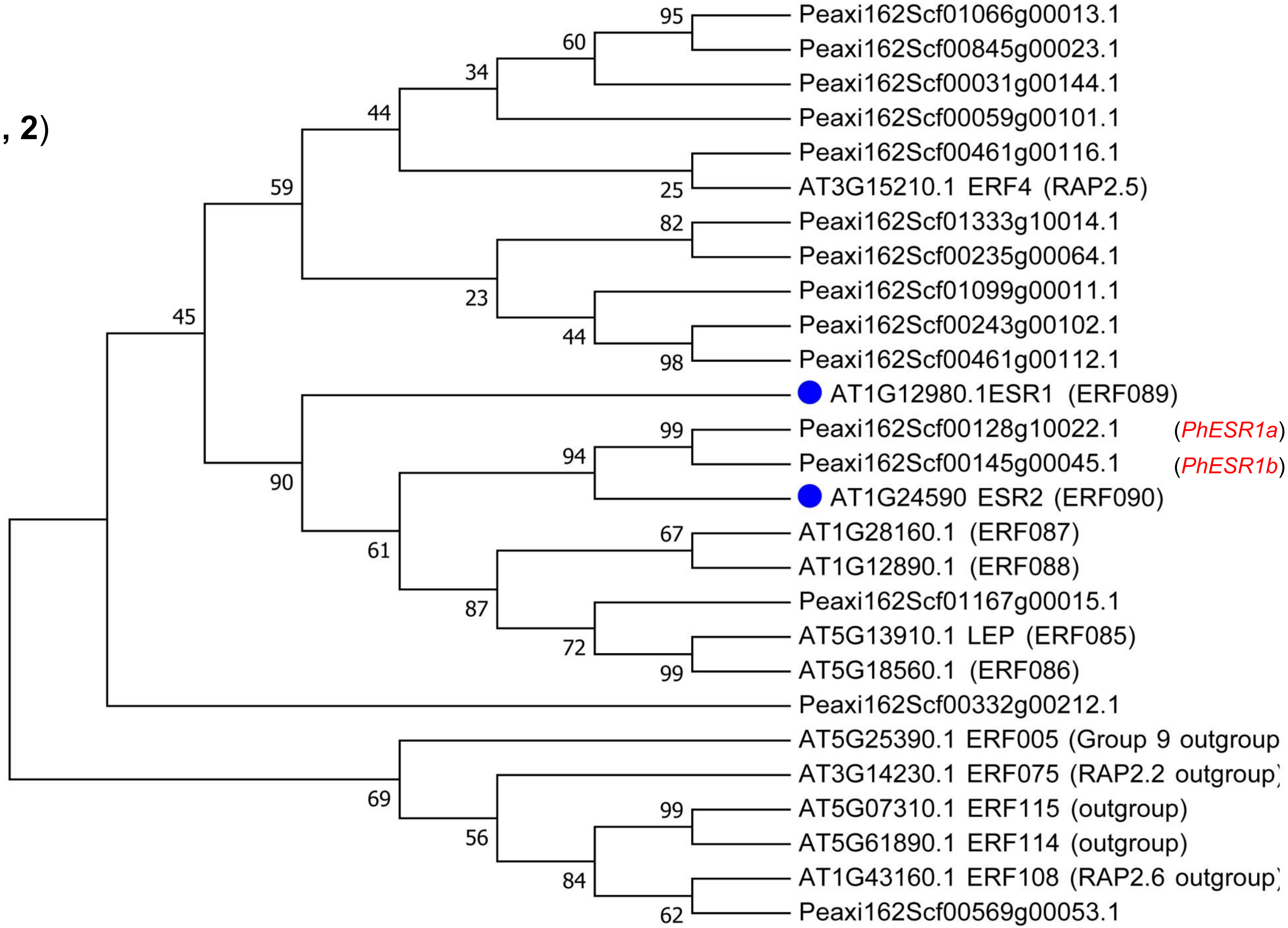

CRFs

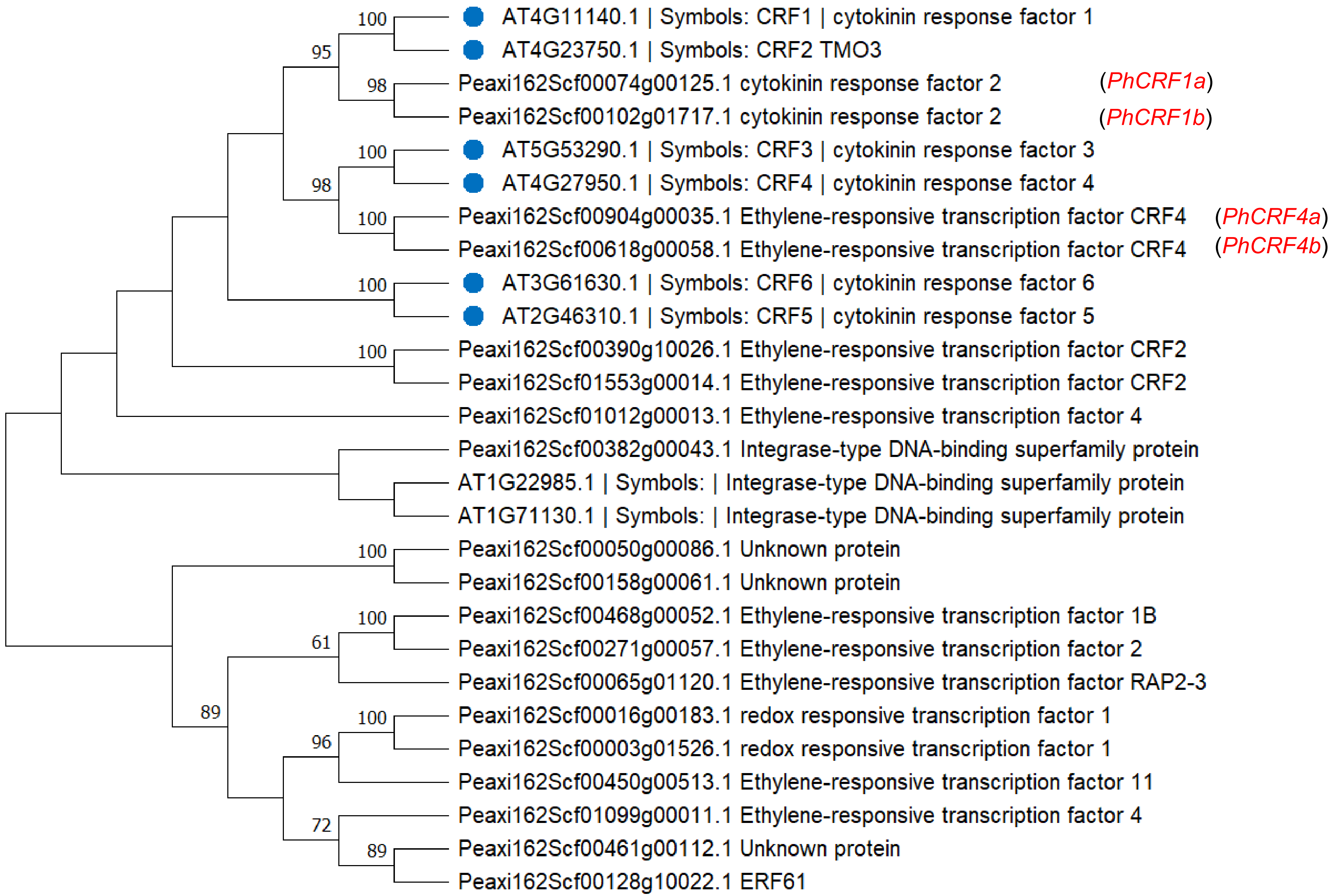
AT4G11140.1 | Symbols: CRF1 | cytokinin response factor 1
AT4G23750.1 | Symbols: CRF2 TMO3
Peaxi162Scf00074g00125.1 cytokinin response factor 2
(PhCRF1a)
Peaxi162Scf00102g01717.1 cytokinin response factor 2
(PhCRF1b)
AT5G53290.1 | Symbols: CRF3 | cytokinin response factor 3
AT4G27950.1 | Symbols: CRF4 | cytokinin response factor 4
Peaxi162Scf00904g00035.1 Ethylene-responsive transcription factor CRF4
(PhCRF4a)
Peaxi162Scf00618g00058.1 Ethylene-responsive transcription factor CRF4
(PhCRF4b)
AT3G61630.1 | Symbols: CRF6 | cytokinin response factor 6
AT2G46310.1 | Symbols: CRF5 | cytokinin response factor 5
Peaxi162Scf00390g10026.1 Ethylene-responsive transcription factor CRF2
Peaxi162Scf01553g00014.1 Ethylene-responsive transcription factor CRF2
Peaxi162Scf01012g00013.1 Ethylene-responsive transcription factor 4
Peaxi162Scf00382g00043.1 Integrase-type DNA-binding superfamily protein
AT1G22985.1 | Symbols: | Integrase-type DNA-binding superfamily protein
AT1G71130.1 | Symbols: | Integrase-type DNA-binding superfamily protein
Peaxi162Scf00050g00086.1 Unknown protein
Peaxi162Scf00158g00061.1 Unknown protein
Peaxi162Scf00468g00052.1 Ethylene-responsive transcription factor 1B
Peaxi162Scf00271g00057.1 Ethylene-responsive transcription factor 2
Peaxi162Scf00065g01120.1 Ethylene-responsive transcription factor RAP2-3
Peaxi162Scf00016g00183.1 redox responsive transcription factor 1
Peaxi162Scf00003g01526.1 redox responsive transcription factor 1
Peaxi162Scf00450g00513.1 Ethylene-responsive transcription factor 11
Peaxi162Scf01099g00011.1 Ethylene-responsive transcription factor 4
Peaxi162Scf00461g00112.1 Unknown protein
Peaxi162Scf00128g10022.1 ERF61
100
95
98
100
98
100
100
100
100
100
61
89
100
96
72
89

JKD genes

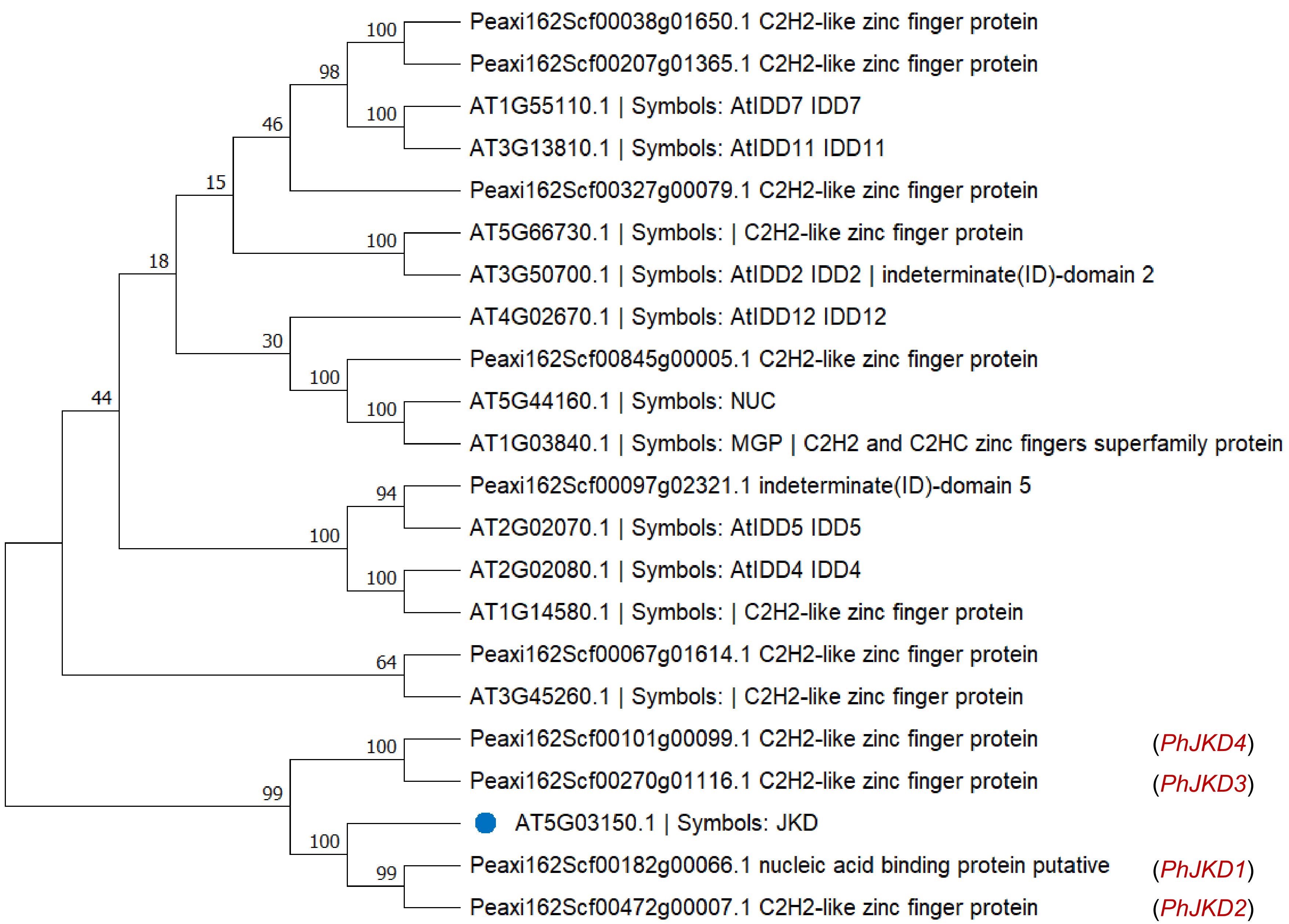

100
98
46
15
18
44
30
100
100
94
100
100
64
100
99
100
99
Peaxi162Scf00038g01650.1 C2H2-like zinc finger protein
Peaxi162Scf00207g01365.1 C2H2-like zinc finger protein
AT1G55110.1 | Symbols: AtIDD7 IDD7
AT3G13810.1 | Symbols: AtIDD11 IDD11
Peaxi162Scf00327g00079.1 C2H2-like zinc finger protein
AT5G66730.1 | Symbols: | C2H2-like zinc finger protein
AT3G50700.1 | Symbols: AtIDD2 IDD2 | indeterminate(ID)-domain 2
AT4G02670.1 | Symbols: AtIDD12 IDD12
Peaxi162Scf00845g00005.1 C2H2-like zinc finger protein
AT5G44160.1 | Symbols: NUC
AT1G03840.1 | Symbols: MGP | C2H2 and C2HC zinc fingers superfamily protein
Peaxi162Scf00097g02321.1 indeterminate(ID)-domain 5
AT2G02070.1 | Symbols: AtIDD5 IDD5
AT2G02080.1 | Symbols: AtIDD4 IDD4
AT1G14580.1 | Symbols: | C2H2-like zinc finger protein
Peaxi162Scf00067g01614.1 C2H2-like zinc finger protein
AT3G45260.1 | Symbols: | C2H2-like zinc finger protein
Peaxi162Scf00101g00099.1 C2H2-like zinc finger protein (PhJKD4)
Peaxi162Scf00270g01116.1 C2H2-like zinc finger protein (PhJKD3)
AT5G03150.1 | Symbols: JKD
Peaxi162Scf00182g00066.1 nucleic acid binding protein putative (PhJKD1)
Peaxi162Scf00472g00007.1 C2H2-like zinc finger protein (PhJKD2)

GRAS

(**CSRRs: SCR, SHR**)

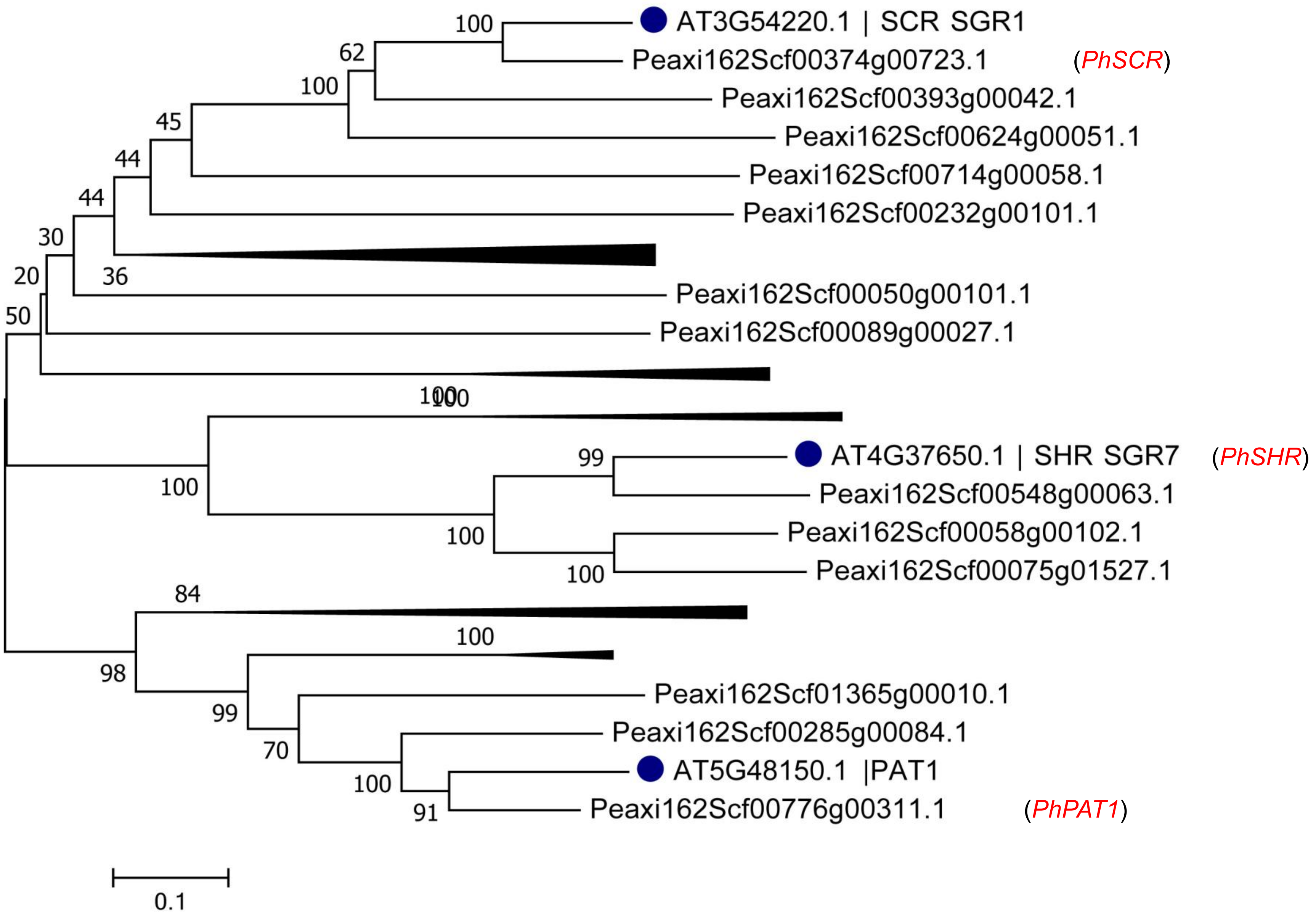

HD-ZIPⅢ family

(**CSRRs: PHB, PHV and REV**)

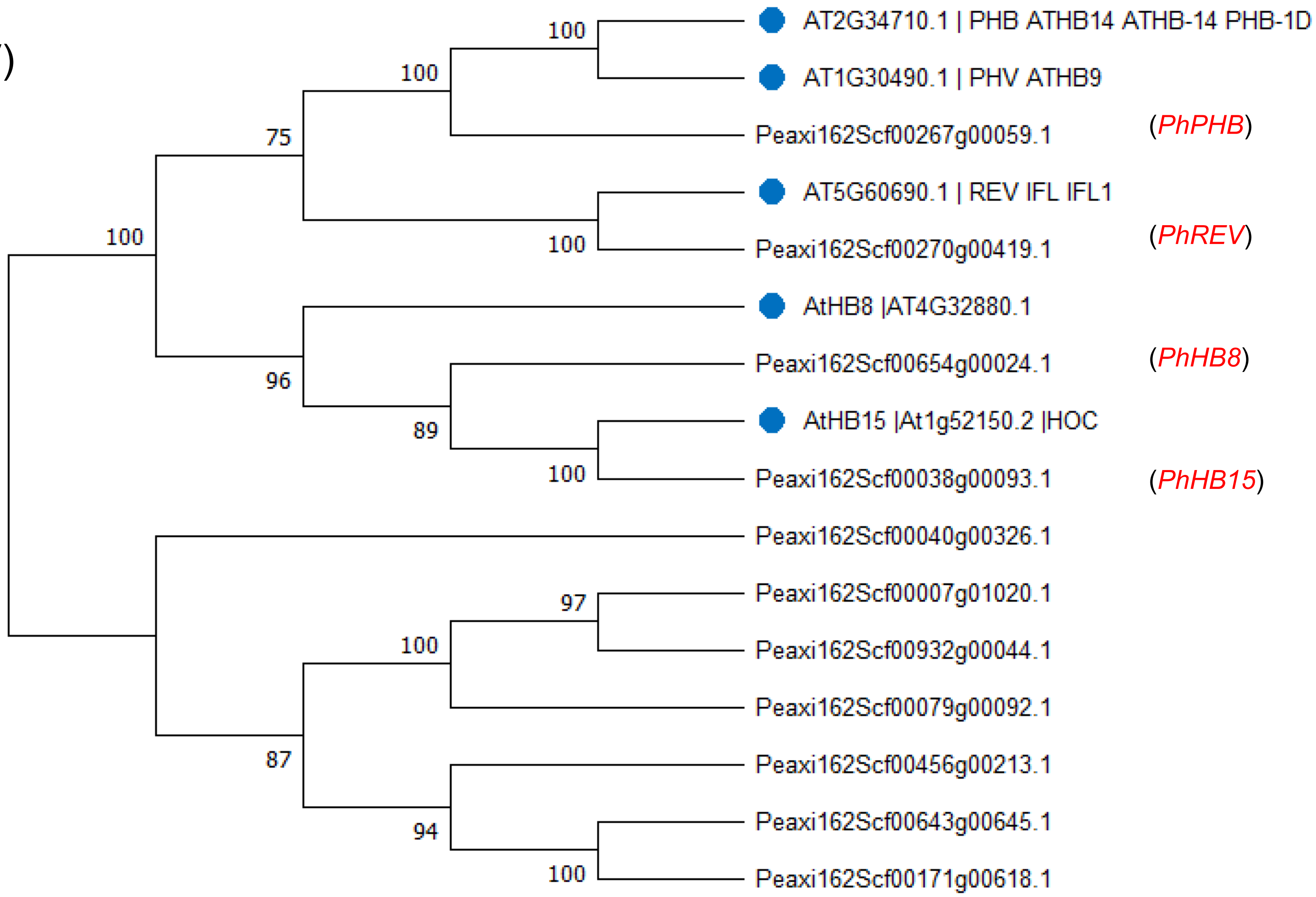

LOB family

(**CSRRs: LBD16, 17, 18, 29**)

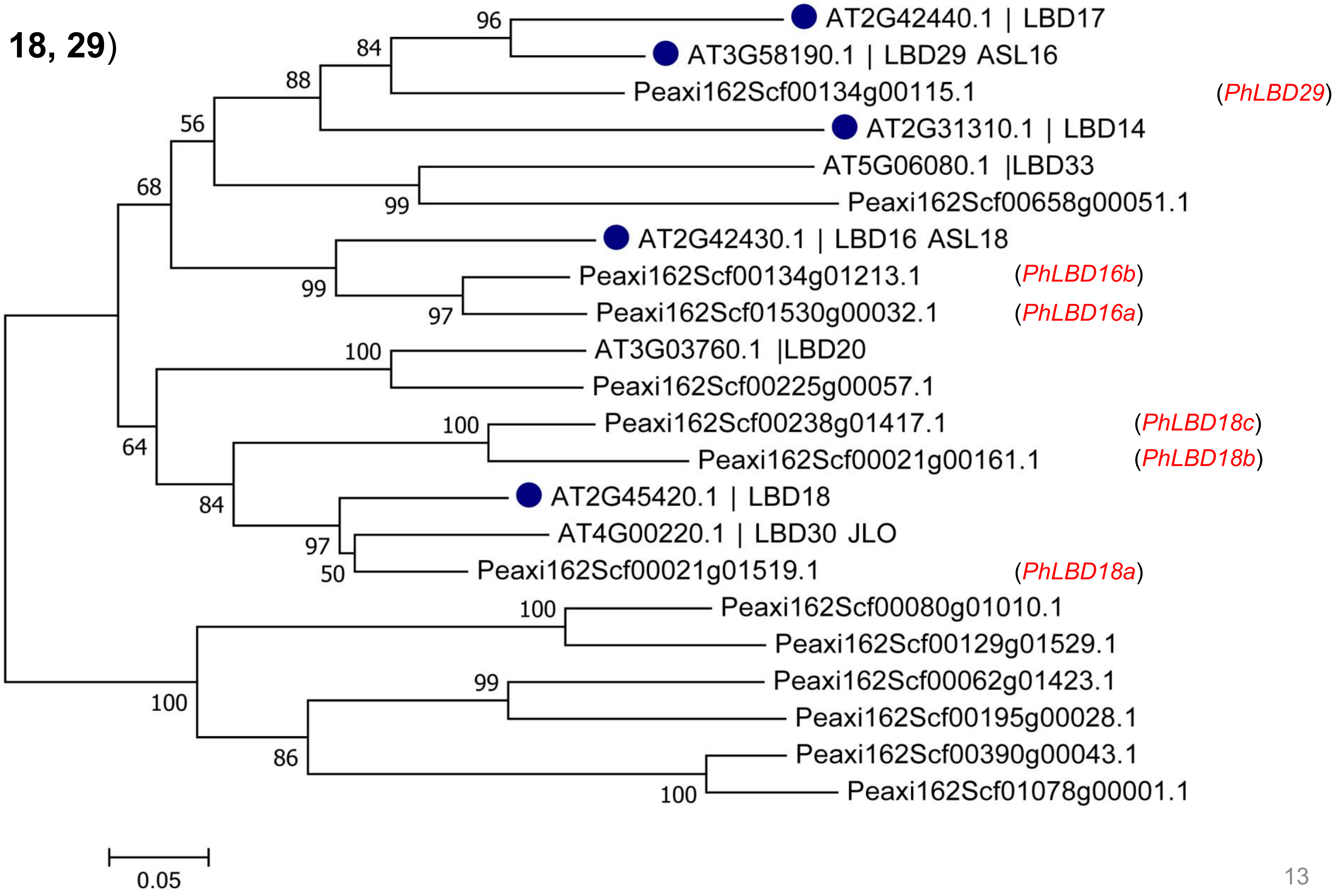

WOX family (**CSRR: WUS, WOX5/7/11/12/13/14**)

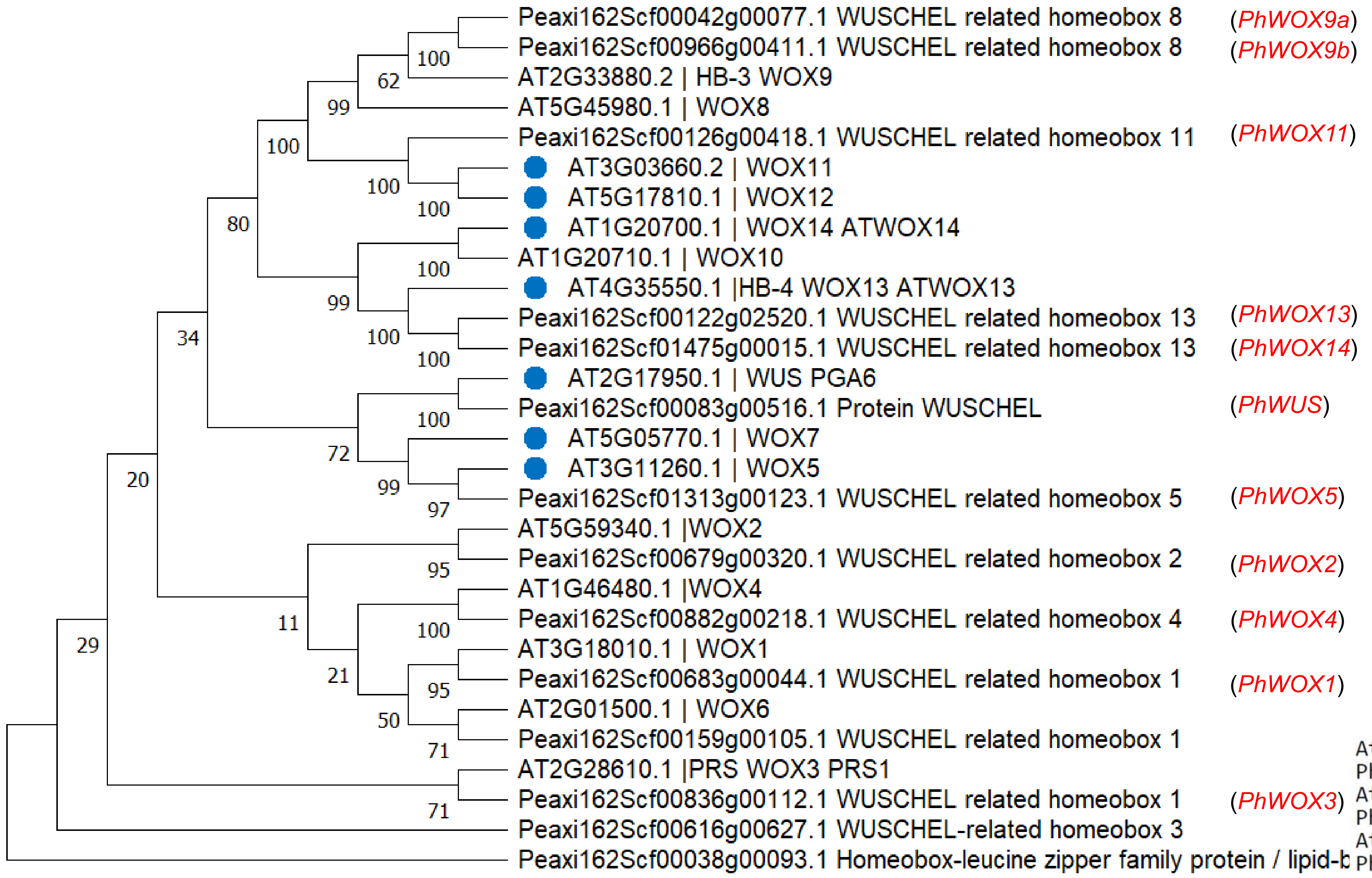

AtWUS	TLPLFPMH	
PhWUS	TLPLFPMH	(Peaxi162Scf00083g0
AtWOX5	TLQLFPVN	
PhWOX5	TLQLFPLN	(Peaxi162Scf01313g0
AtWOX13	--------	
PhWOX13	--------	(Peaxi162Scf00122g0
AtWOX14	EHILFPSP	
PhWOX14	HEPLFPSE	(Peaxi162Scf01475g0

NAC family

(**CSRRs: CUC1, 2**)

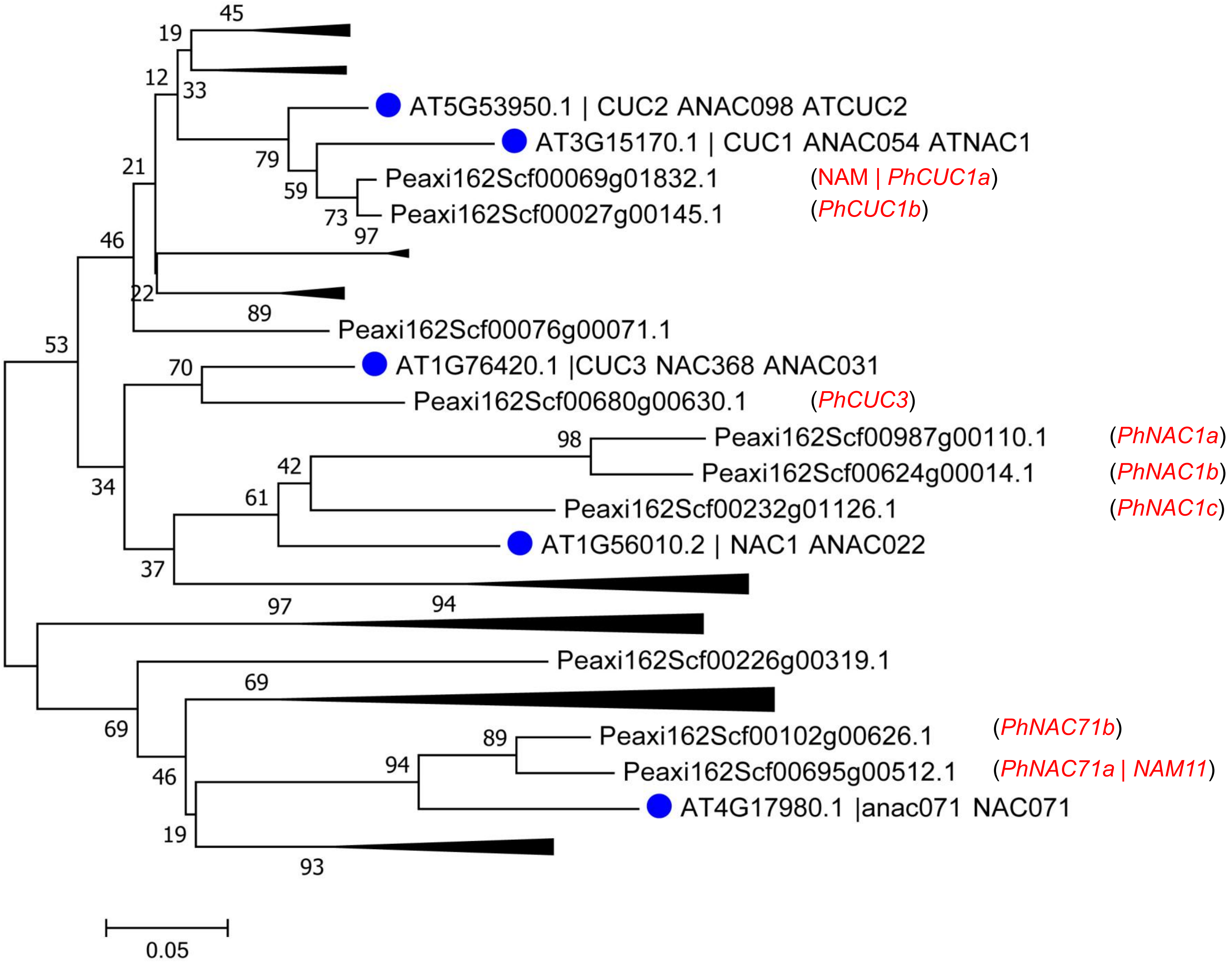

KNOX family
(**CSRR: STM**)

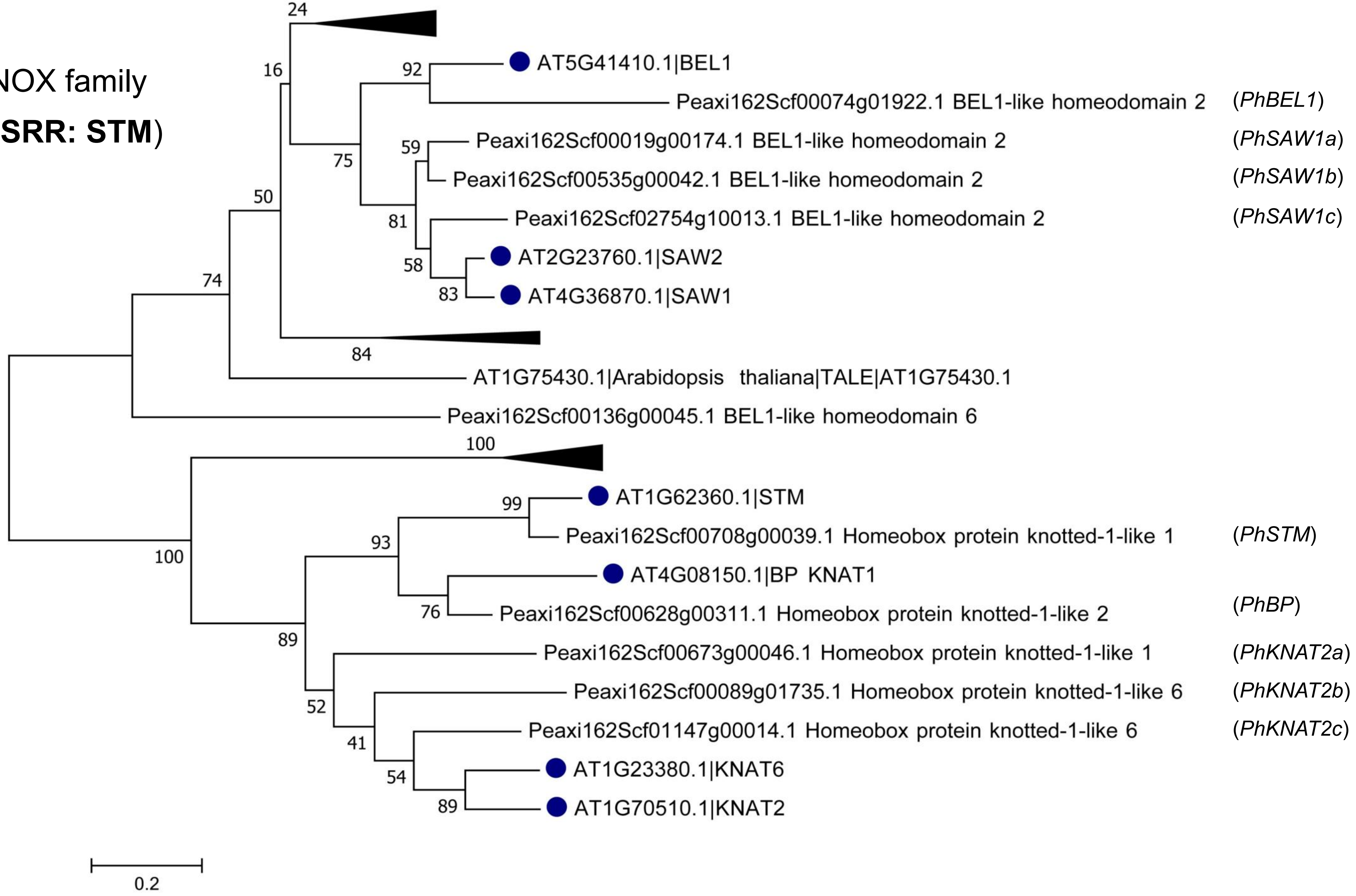

Dof family

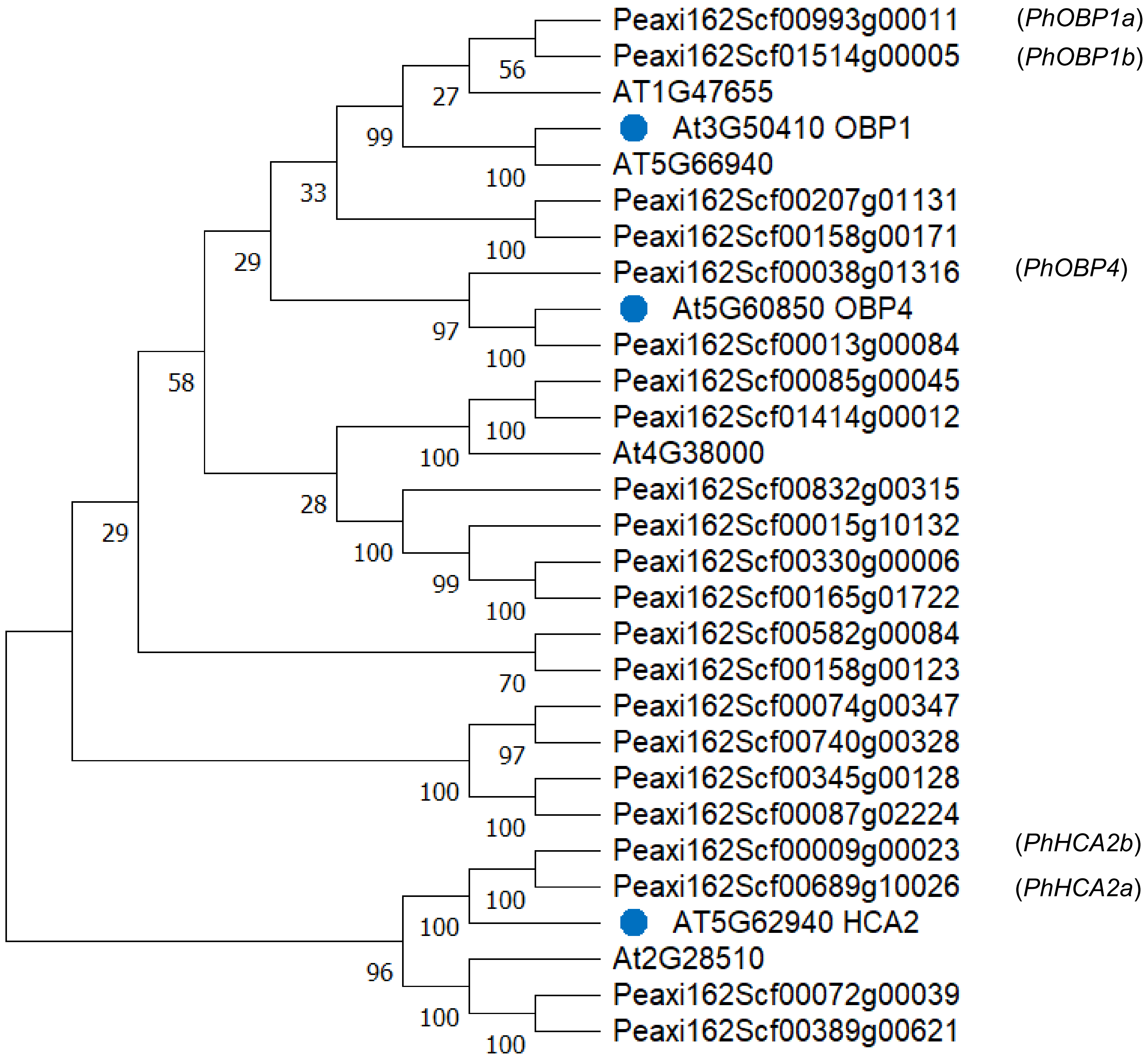

Peaxi162Scf00993g00011
(PhOBP1a)
Peaxi162Scf01514g00005
(PhOBP1b)
AT1G47655
At3G50410 OBP1
AT5G66940
Peaxi162Scf00207g01131
Peaxi162Scf00158g00171
Peaxi162Scf00038g01316
(PhOBP4)
At5G60850 OBP4
Peaxi162Scf00013g00084
Peaxi162Scf00085g00045
Peaxi162Scf01414g00012
At4G38000
Peaxi162Scf00832g00315
Peaxi162Scf00015g10132
Peaxi162Scf00330g00006
Peaxi162Scf00165g01722
Peaxi162Scf00582g00084
Peaxi162Scf00158g00123
Peaxi162Scf00074g00347
Peaxi162Scf00740g00328
Peaxi162Scf00345g00128
Peaxi162Scf00087g02224
Peaxi162Scf00009g00023
(PhHCA2b)
Peaxi162Scf00689g10026
(PhHCA2a)
AT5G62940 HCA2
At2G28510
Peaxi162Scf00072g00039
Peaxi162Scf00389g00621
56
27
99
100
33
100
29
97
100
58
100
100
28
100
99
100
29
70
97
100
100
100
100
100
96
100
100

E2Fa

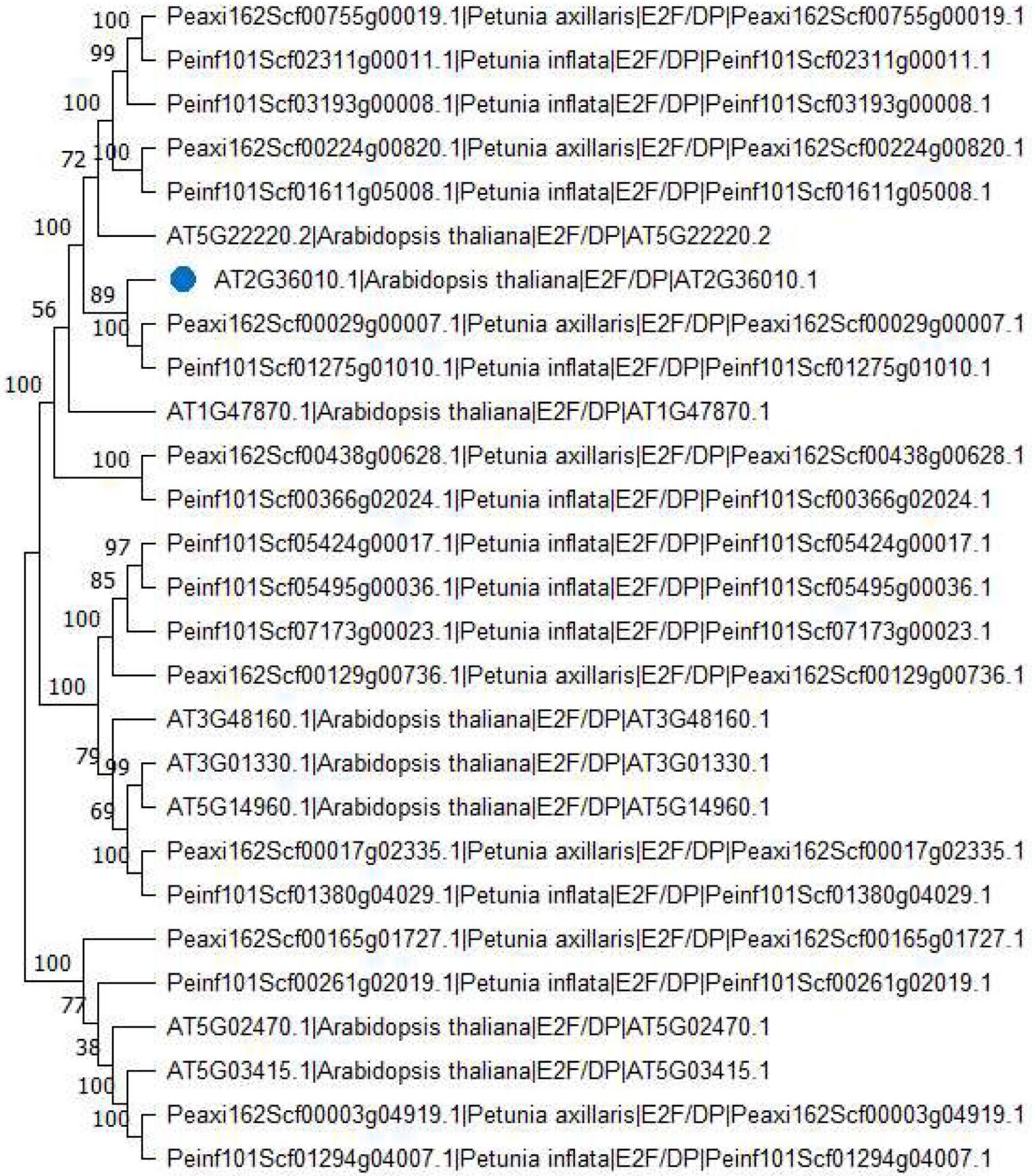

Peaxi162Scf00755g00019.1|Petunia axillaris|E2F/DP|Peaxi162Scf00755g00019.1
Peinf101Scf02311g00011.1|Petunia inflata|E2F/DP|Peinf101Scf02311g00011.1
Peinf101Scf03193g00008.1|Petunia inflata|E2F/DP|Peinf101Scf03193g00008.1
Peaxi162Scf00224g00820.1|Petunia axillaris|E2F/DP|Peaxi162Scf00224g00820.1
Peinf101Scf01611g05008.1|Petunia inflata|E2F/DP|Peinf101Scf01611g05008.1
AT5G22220.2|Arabidopsis thaliana|E2F/DP|AT5G22220.2
AT2G36010.1|Arabidopsis thaliana|E2F/DP|AT2G36010.1
Peaxi162Scf00029g00007.1|Petunia axillaris|E2F/DP|Peaxi162Scf00029g00007.1
Peinf101Scf01275g01010.1|Petunia inflata|E2F/DP|Peinf101Scf01275g01010.1
AT1G47870.1|Arabidopsis thaliana|E2F/DP|AT1G47870.1
Peaxi162Scf00438g00628.1|Petunia axillaris|E2F/DP|Peaxi162Scf00438g00628.1
Peinf101Scf00366g02024.1|Petunia inflata|E2F/DP|Peinf101Scf00366g02024.1
Peinf101Scf05424g00017.1|Petunia inflata|E2F/DP|Peinf101Scf05424g00017.1
Peinf101Scf05495g00036.1|Petunia inflata|E2F/DP|Peinf101Scf05495g00036.1
Peinf101Scf07173g00023.1|Petunia inflata|E2F/DP|Peinf101Scf07173g00023.1
Peaxi162Scf00129g00736.1|Petunia axillaris|E2F/DP|Peaxi162Scf00129g00736.1
AT3G48160.1|Arabidopsis thaliana|E2F/DP|AT3G48160.1
AT3G01330.1|Arabidopsis thaliana|E2F/DP|AT3G01330.1
AT5G14960.1|Arabidopsis thaliana|E2F/DP|AT5G14960.1
Peaxi162Scf00017g02335.1|Petunia axillaris|E2F/DP|Peaxi162Scf00017g02335.1
Peinf101Scf01380g04029.1|Petunia inflata|E2F/DP|Peinf101Scf01380g04029.1
Peaxi162Scf00165g01727.1|Petunia axillaris|E2F/DP|Peaxi162Scf00165g01727.1
Peinf101Scf00261g02019.1|Petunia inflata|E2F/DP|Peinf101Scf00261g02019.1
AT5G02470.1|Arabidopsis thaliana|E2F/DP|AT5G02470.1
AT5G03415.1|Arabidopsis thaliana|E2F/DP|AT5G03415.1
Peaxi162Scf00003g04919.1|Petunia axillaris|E2F/DP|Peaxi162Scf00003g04919.1
Peinf101Scf01294g04007.1|Petunia inflata|E2F/DP|Peinf101Scf01294g04007.1

(*PhE2Fa*)

B3 family

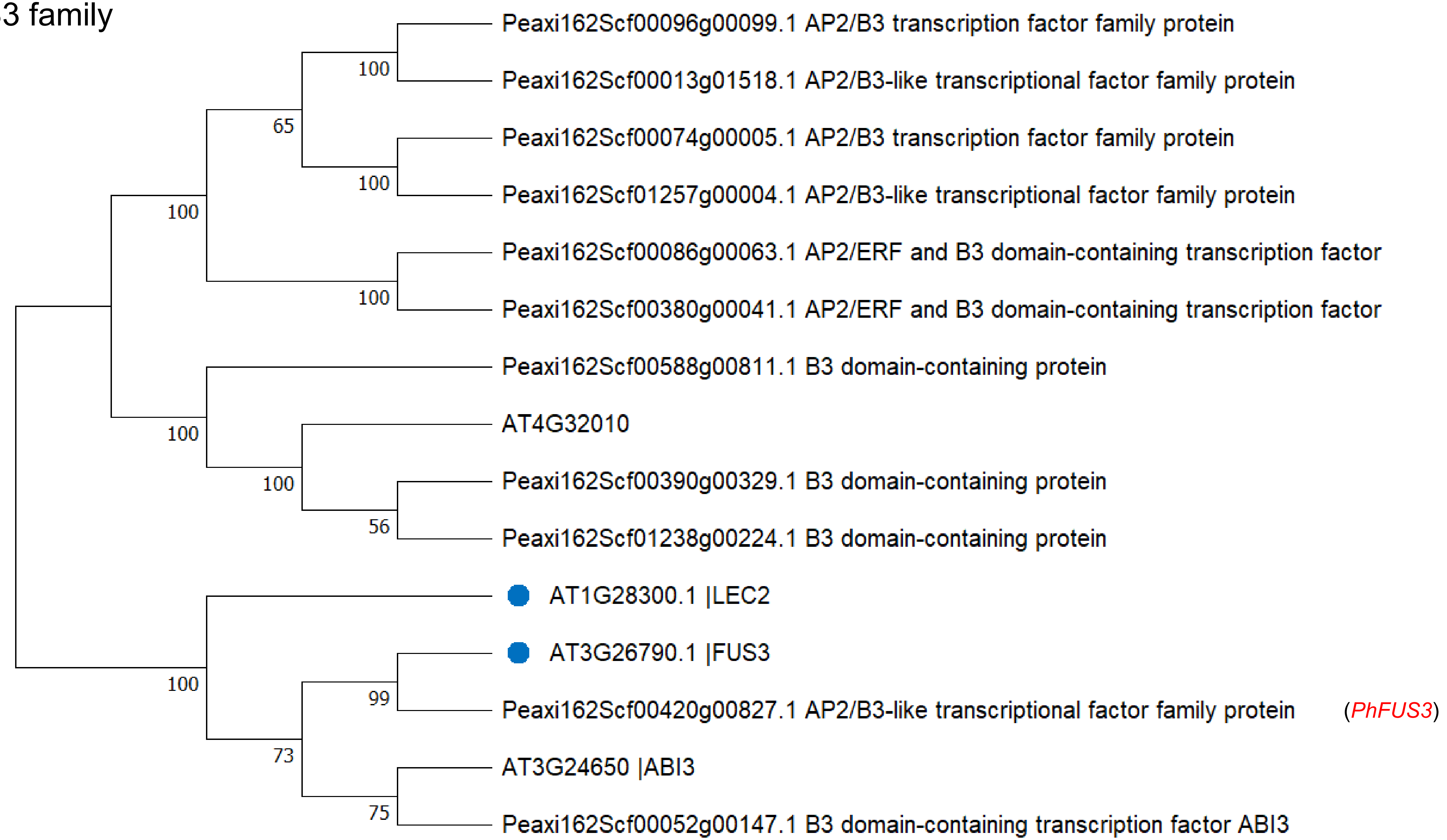
Peaxi162Scf00096g00099.1 AP2/B3 transcription factor family protein
Peaxi162Scf00013g01518.1 AP2/B3-like transcriptional factor family protein
Peaxi162Scf00074g00005.1 AP2/B3 transcription factor family protein
Peaxi162Scf01257g00004.1 AP2/B3-like transcriptional factor family protein
Peaxi162Scf00086g00063.1 AP2/ERF and B3 domain-containing transcription factor
Peaxi162Scf00380g00041.1 AP2/ERF and B3 domain-containing transcription factor
Peaxi162Scf00588g00811.1 B3 domain-containing protein
AT4G32010
Peaxi162Scf00390g00329.1 B3 domain-containing protein
Peaxi162Scf01238g00224.1 B3 domain-containing protein
AT1G28300.1 |LEC2
AT3G26790.1 |FUS3
Peaxi162Scf00420g00827.1 AP2/B3-like transcriptional factor family protein
(PhFUS3)
AT3G24650 |ABI3
Peaxi162Scf00052g00147.1 B3 domain-containing transcription factor ABI3
100
65
100
100
100
100
100
100
56
100
99
73
75

NF-YB family

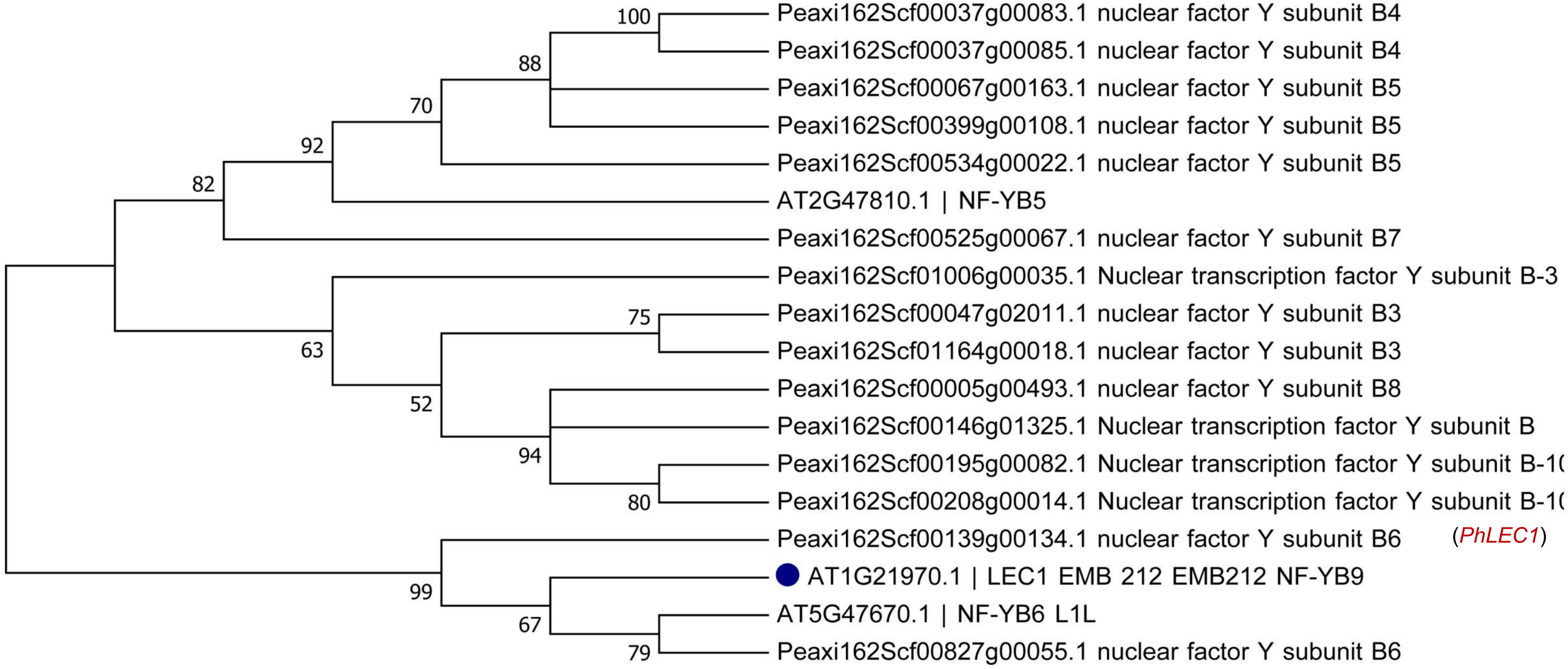

100
88
70
92
82
75
63
52
94
80
99
67
79
Peaxi162Scf00037g00083.1 nuclear factor Y subunit B4
Peaxi162Scf00037g00085.1 nuclear factor Y subunit B4
Peaxi162Scf00067g00163.1 nuclear factor Y subunit B5
Peaxi162Scf00399g00108.1 nuclear factor Y subunit B5
Peaxi162Scf00534g00022.1 nuclear factor Y subunit B5
AT2G47810.1 | NF-YB5
Peaxi162Scf00525g00067.1 nuclear factor Y subunit B7
Peaxi162Scf01006g00035.1 Nuclear transcription factor Y subunit B-3
Peaxi162Scf00047g02011.1 nuclear factor Y subunit B3
Peaxi162Scf01164g00018.1 nuclear factor Y subunit B3
Peaxi162Scf00005g00493.1 nuclear factor Y subunit B8
Peaxi162Scf00146g01325.1 Nuclear transcription factor Y subunit B
Peaxi162Scf00195g00082.1 Nuclear transcription factor Y subunit B-1(
Peaxi162Scf00208g00014.1 Nuclear transcription factor Y subunit B-1(
Peaxi162Scf00139g00134.1 nuclear factor Y subunit B6
(*PhLEC1*)
AT1G21970.1 | LEC1 EMB 212 EMB212 NF-YB9
AT5G47670.1 | NF-YB6 L1L
Peaxi162Scf00827g00055.1 nuclear factor Y subunit B6

RKD4

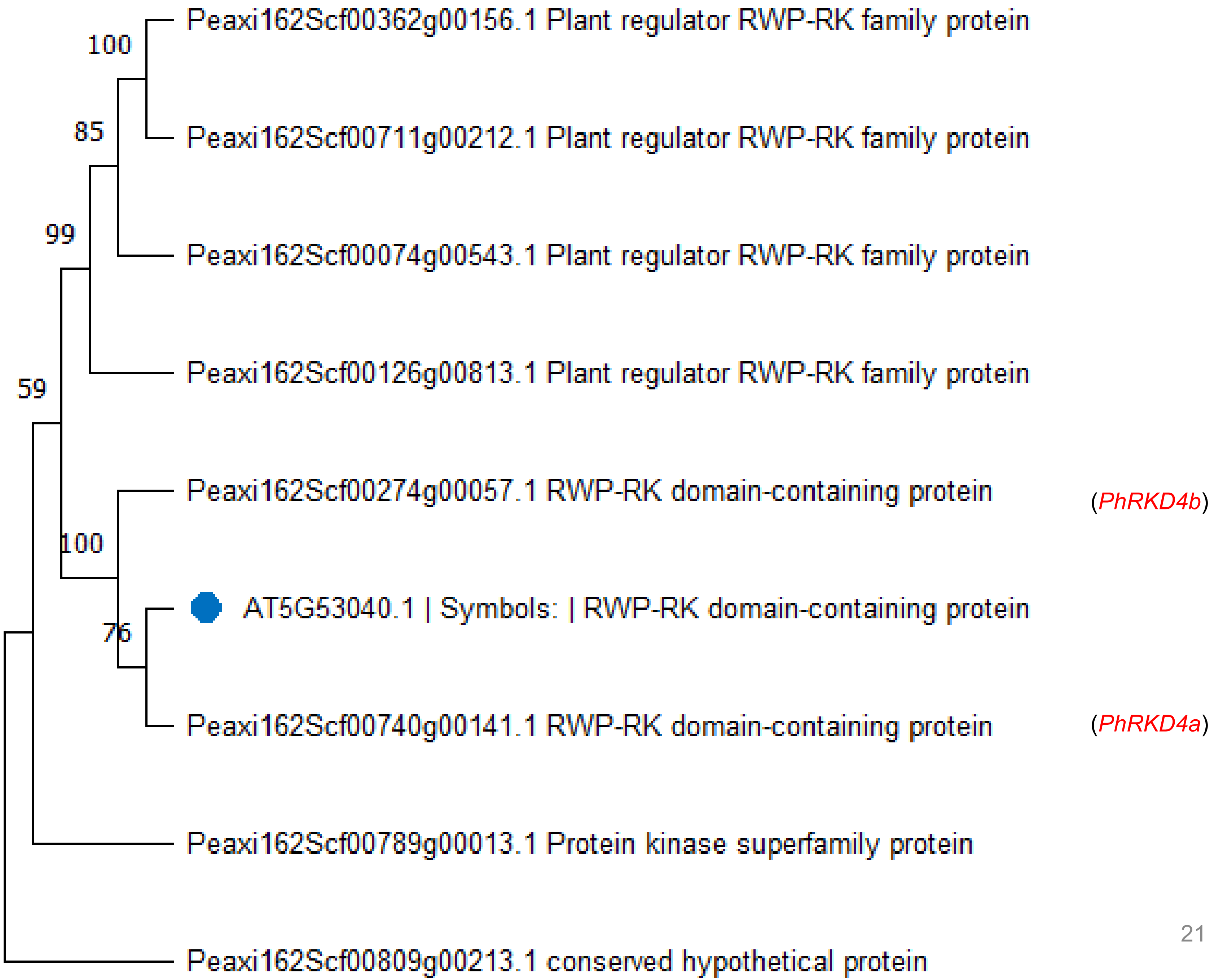
Peaxi162Scf00362g00156.1 Plant regulator RWP-RK family protein
Peaxi162Scf00711g00212.1 Plant regulator RWP-RK family protein
Peaxi162Scf00074g00543.1 Plant regulator RWP-RK family protein
Peaxi162Scf00126g00813.1 Plant regulator RWP-RK family protein
Peaxi162Scf00274g00057.1 RWP-RK domain-containing protein
(PhRKD4b)
AT5G53040.1 | Symbols: | RWP-RK domain-containing protein
Peaxi162Scf00740g00141.1 RWP-RK domain-containing protein
(PhRKD4a)
Peaxi162Scf00789g00013.1 Protein kinase superfamily protein
Peaxi162Scf00809g00213.1 conserved hypothetical protein
100
85
99
59
100
76

GRF5

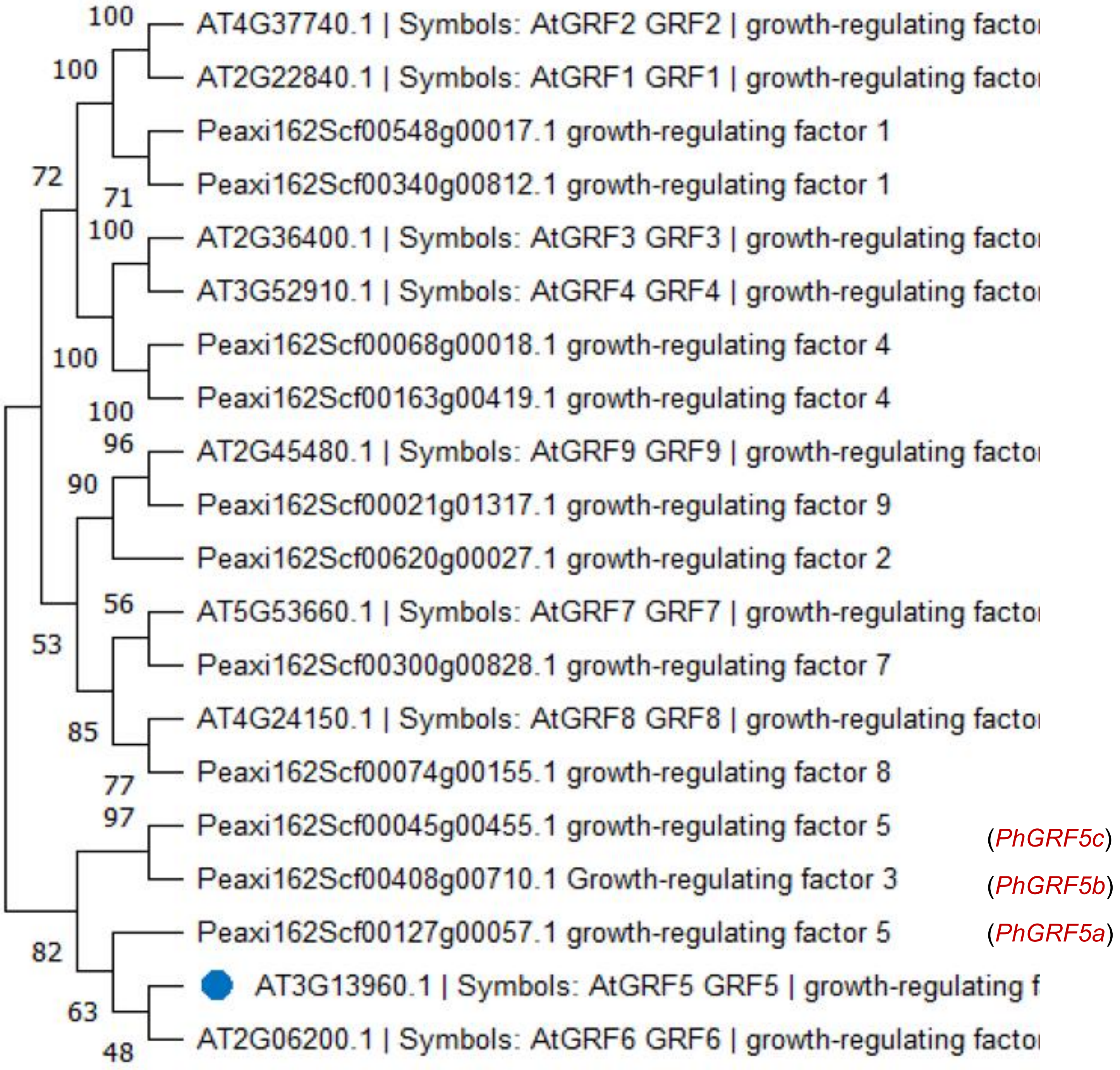

100
100
72
71
100
100
100
96
90
56
53
85
77
97
82
63
48
AT4G37740.1 | Symbols: AtGRF2 GRF2 | growth-regulating factor
AT2G22840.1 | Symbols: AtGRF1 GRF1 | growth-regulating factor
Peaxi162Scf00548g00017.1 growth-regulating factor 1
Peaxi162Scf00340g00812.1 growth-regulating factor 1
AT2G36400.1 | Symbols: AtGRF3 GRF3 | growth-regulating factor
AT3G52910.1 | Symbols: AtGRF4 GRF4 | growth-regulating factor
Peaxi162Scf00068g00018.1 growth-regulating factor 4
Peaxi162Scf00163g00419.1 growth-regulating factor 4
AT2G45480.1 | Symbols: AtGRF9 GRF9 | growth-regulating factor
Peaxi162Scf00021g01317.1 growth-regulating factor 9
Peaxi162Scf00620g00027.1 growth-regulating factor 2
AT5G53660.1 | Symbols: AtGRF7 GRF7 | growth-regulating factor
Peaxi162Scf00300g00828.1 growth-regulating factor 7
AT4G24150.1 | Symbols: AtGRF8 GRF8 | growth-regulating factor
Peaxi162Scf00074g00155.1 growth-regulating factor 8
Peaxi162Scf00045g00455.1 growth-regulating factor 5
Peaxi162Scf00408g00710.1 Growth-regulating factor 3
Peaxi162Scf00127g00057.1 growth-regulating factor 5
AT3G13960.1 | Symbols: AtGRF5 GRF5 | growth-regulating f
AT2G06200.1 | Symbols: AtGRF6 GRF6 | growth-regulating factor
(PhGRF5c)
(PhGRF5b)
(PhGRF5a)

WRKY23

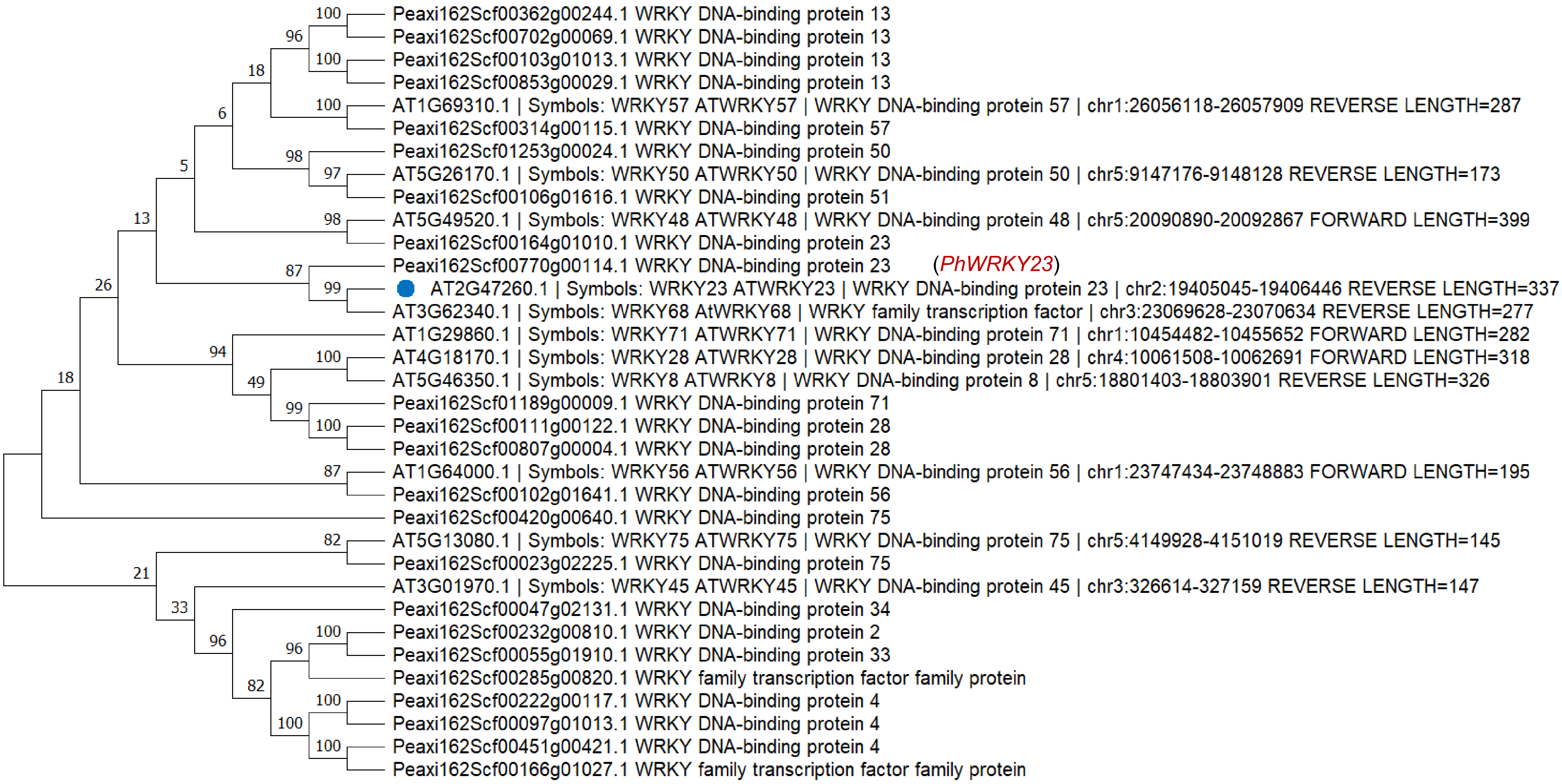
Peaxi162Scf00362g00244.1 WRKY DNA-binding protein 13
Peaxi162Scf00702g00069.1 WRKY DNA-binding protein 13
Peaxi162Scf00103g01013.1 WRKY DNA-binding protein 13
Peaxi162Scf00853g00029.1 WRKY DNA-binding protein 13
AT1G69310.1 | Symbols: WRKY57 ATWRKY57 | WRKY DNA-binding protein 57 | chr1:26056118-26057909 REVERSE LENGTH=287
Peaxi162Scf00314g00115.1 WRKY DNA-binding protein 57
Peaxi162Scf01253g00024.1 WRKY DNA-binding protein 50
AT5G26170.1 | Symbols: WRKY50 ATWRKY50 | WRKY DNA-binding protein 50 | chr5:9147176-9148128 REVERSE LENGTH=173
Peaxi162Scf00106g01616.1 WRKY DNA-binding protein 51
AT5G49520.1 | Symbols: WRKY48 ATWRKY48 | WRKY DNA-binding protein 48 | chr5:20090890-20092867 FORWARD LENGTH=399
Peaxi162Scf00164g01010.1 WRKY DNA-binding protein 23
Peaxi162Scf00770g00114.1 WRKY DNA-binding protein 23
(PhWRKY23)
AT2G47260.1 | Symbols: WRKY23 ATWRKY23 | WRKY DNA-binding protein 23 | chr2:19405045-19406446 REVERSE LENGTH=337
AT3G62340.1 | Symbols: WRKY68 AtWRKY68 | WRKY family transcription factor | chr3:23069628-23070634 REVERSE LENGTH=277
AT1G29860.1 | Symbols: WRKY71 ATWRKY71 | WRKY DNA-binding protein 71 | chr1:10454482-10455652 FORWARD LENGTH=282
AT4G18170.1 | Symbols: WRKY28 ATWRKY28 | WRKY DNA-binding protein 28 | chr4:10061508-10062691 FORWARD LENGTH=318
AT5G46350.1 | Symbols: WRKY8 ATWRKY8 | WRKY DNA-binding protein 8 | chr5:18801403-18803901 REVERSE LENGTH=326
Peaxi162Scf01189g00009.1 WRKY DNA-binding protein 71
Peaxi162Scf00111g00122.1 WRKY DNA-binding protein 28
Peaxi162Scf00807g00004.1 WRKY DNA-binding protein 28
AT1G64000.1 | Symbols: WRKY56 ATWRKY56 | WRKY DNA-binding protein 56 | chr1:23747434-23748883 FORWARD LENGTH=195
Peaxi162Scf00102g01641.1 WRKY DNA-binding protein 56
Peaxi162Scf00420g00640.1 WRKY DNA-binding protein 75
AT5G13080.1 | Symbols: WRKY75 ATWRKY75 | WRKY DNA-binding protein 75 | chr5:4149928-4151019 REVERSE LENGTH=145
Peaxi162Scf00023g02225.1 WRKY DNA-binding protein 75
AT3G01970.1 | Symbols: WRKY45 ATWRKY45 | WRKY DNA-binding protein 45 | chr3:326614-327159 REVERSE LENGTH=147
Peaxi162Scf00047g02131.1 WRKY DNA-binding protein 34
Peaxi162Scf00232g00810.1 WRKY DNA-binding protein 2
Peaxi162Scf00055g01910.1 WRKY DNA-binding protein 33
Peaxi162Scf00285g00820.1 WRKY family transcription factor family protein
Peaxi162Scf00222g00117.1 WRKY DNA-binding protein 4
Peaxi162Scf00097g01013.1 WRKY DNA-binding protein 4
Peaxi162Scf00451g00421.1 WRKY DNA-binding protein 4
Peaxi162Scf00166g01027.1 WRKY family transcription factor family protein

bHLH41

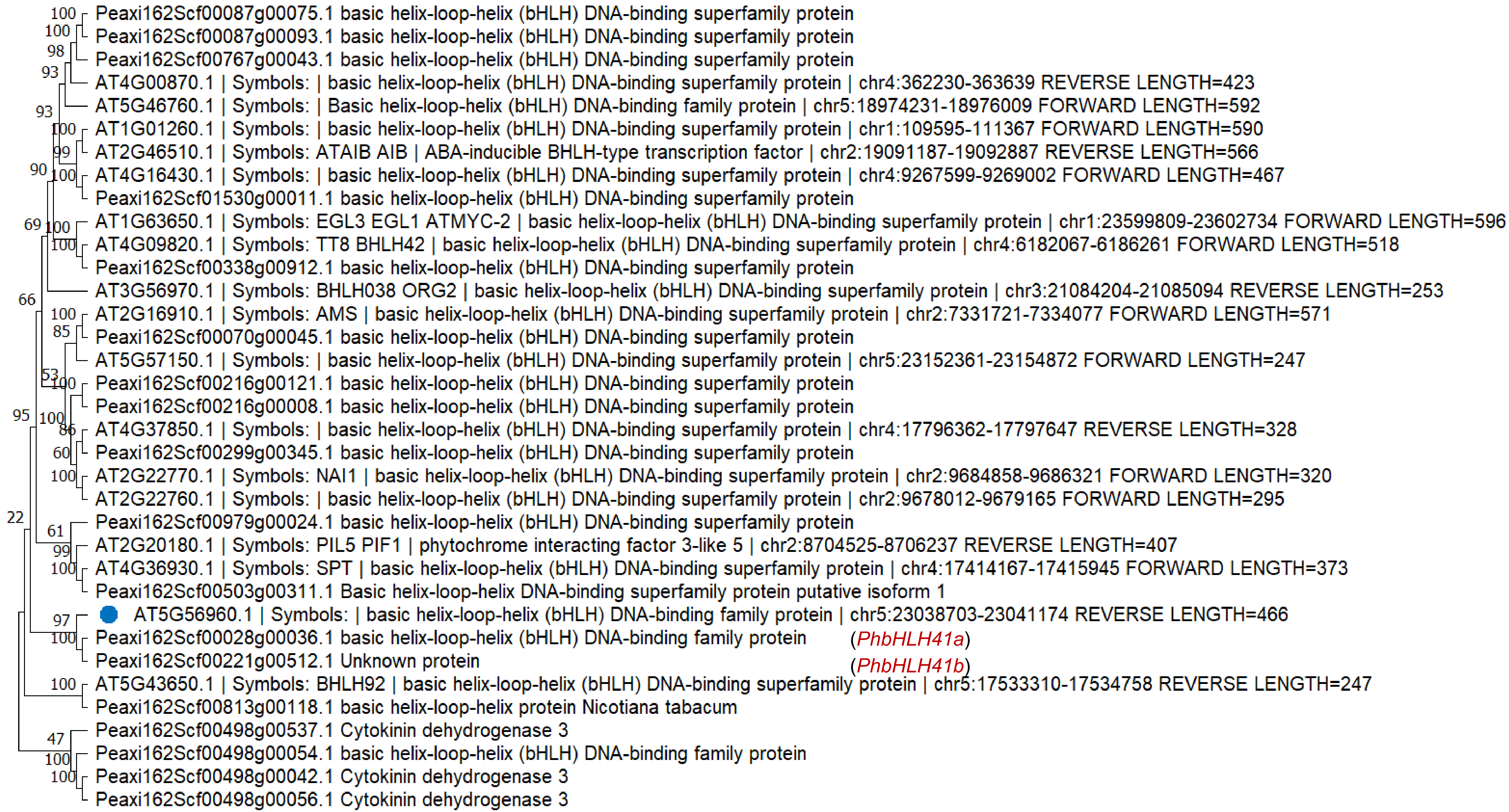

Peaxi162Scf00087g00075.1 basic helix-loop-helix (bHLH) DNA-binding superfamily protein
Peaxi162Scf00087g00093.1 basic helix-loop-helix (bHLH) DNA-binding superfamily protein
Peaxi162Scf00767g00043.1 basic helix-loop-helix (bHLH) DNA-binding superfamily protein
AT4G00870.1 | Symbols: | basic helix-loop-helix (bHLH) DNA-binding superfamily protein | chr4:362230-363639 REVERSE LENGTH=423
AT5G46760.1 | Symbols: | Basic helix-loop-helix (bHLH) DNA-binding family protein | chr5:18974231-18976009 FORWARD LENGTH=592
AT1G01260.1 | Symbols: | basic helix-loop-helix (bHLH) DNA-binding superfamily protein | chr1:109595-111367 FORWARD LENGTH=590
AT2G46510.1 | Symbols: ATAIB AIB | ABA-inducible BHLH-type transcription factor | chr2:19091187-19092887 REVERSE LENGTH=566
AT4G16430.1 | Symbols: | basic helix-loop-helix (bHLH) DNA-binding superfamily protein | chr4:9267599-9269002 FORWARD LENGTH=467
Peaxi162Scf01530g00011.1 basic helix-loop-helix (bHLH) DNA-binding superfamily protein
AT1G63650.1 | Symbols: EGL3 EGL1 ATMYC-2 | basic helix-loop-helix (bHLH) DNA-binding superfamily protein | chr1:23599809-23602734 FORWARD LENGTH=596
AT4G09820.1 | Symbols: TT8 BHLH42 | basic helix-loop-helix (bHLH) DNA-binding superfamily protein | chr4:6182067-6186261 FORWARD LENGTH=518
Peaxi162Scf00338g00912.1 basic helix-loop-helix (bHLH) DNA-binding superfamily protein
AT3G56970.1 | Symbols: BHLH038 ORG2 | basic helix-loop-helix (bHLH) DNA-binding superfamily protein | chr3:21084204-21085094 REVERSE LENGTH=253
AT2G16910.1 | Symbols: AMS | basic helix-loop-helix (bHLH) DNA-binding superfamily protein | chr2:7331721-7334077 FORWARD LENGTH=571
Peaxi162Scf00070g00045.1 basic helix-loop-helix (bHLH) DNA-binding superfamily protein
AT5G57150.1 | Symbols: | basic helix-loop-helix (bHLH) DNA-binding superfamily protein | chr5:23152361-23154872 FORWARD LENGTH=247
Peaxi162Scf00216g00121.1 basic helix-loop-helix (bHLH) DNA-binding superfamily protein
Peaxi162Scf00216g00008.1 basic helix-loop-helix (bHLH) DNA-binding superfamily protein
AT4G37850.1 | Symbols: | basic helix-loop-helix (bHLH) DNA-binding superfamily protein | chr4:17796362-17797647 REVERSE LENGTH=328
Peaxi162Scf00299g00345.1 basic helix-loop-helix (bHLH) DNA-binding superfamily protein
AT2G22770.1 | Symbols: NAI1 | basic helix-loop-helix (bHLH) DNA-binding superfamily protein | chr2:9684858-9686321 FORWARD LENGTH=320
AT2G22760.1 | Symbols: | basic helix-loop-helix (bHLH) DNA-binding superfamily protein | chr2:9678012-9679165 FORWARD LENGTH=295
Peaxi162Scf00979g00024.1 basic helix-loop-helix (bHLH) DNA-binding superfamily protein
AT2G20180.1 | Symbols: PIL5 PIF1 | phytochrome interacting factor 3-like 5 | chr2:8704525-8706237 REVERSE LENGTH=407
AT4G36930.1 | Symbols: SPT | basic helix-loop-helix (bHLH) DNA-binding superfamily protein | chr4:17414167-17415945 FORWARD LENGTH=373
Peaxi162Scf00503g00311.1 Basic helix-loop-helix DNA-binding superfamily protein putative isoform 1
AT5G56960.1 | Symbols: | basic helix-loop-helix (bHLH) DNA-binding family protein | chr5:23038703-23041174 REVERSE LENGTH=466
Peaxi162Scf00028g00036.1 basic helix-loop-helix (bHLH) DNA-binding family protein
(PhbHLH41a)
Peaxi162Scf00221g00512.1 Unknown protein
(PhbHLH41b)
AT5G43650.1 | Symbols: BHLH92 | basic helix-loop-helix (bHLH) DNA-binding superfamily protein | chr5:17533310-17534758 REVERSE LENGTH=247
Peaxi162Scf00813g00118.1 basic helix-loop-helix protein Nicotiana tabacum
Peaxi162Scf00498g00537.1 Cytokinin dehydrogenase 3
Peaxi162Scf00498g00054.1 basic helix-loop-helix (bHLH) DNA-binding family protein
Peaxi162Scf00498g00042.1 Cytokinin dehydrogenase 3
Peaxi162Scf00498g00056.1 Cytokinin dehydrogenase 3

bZip59

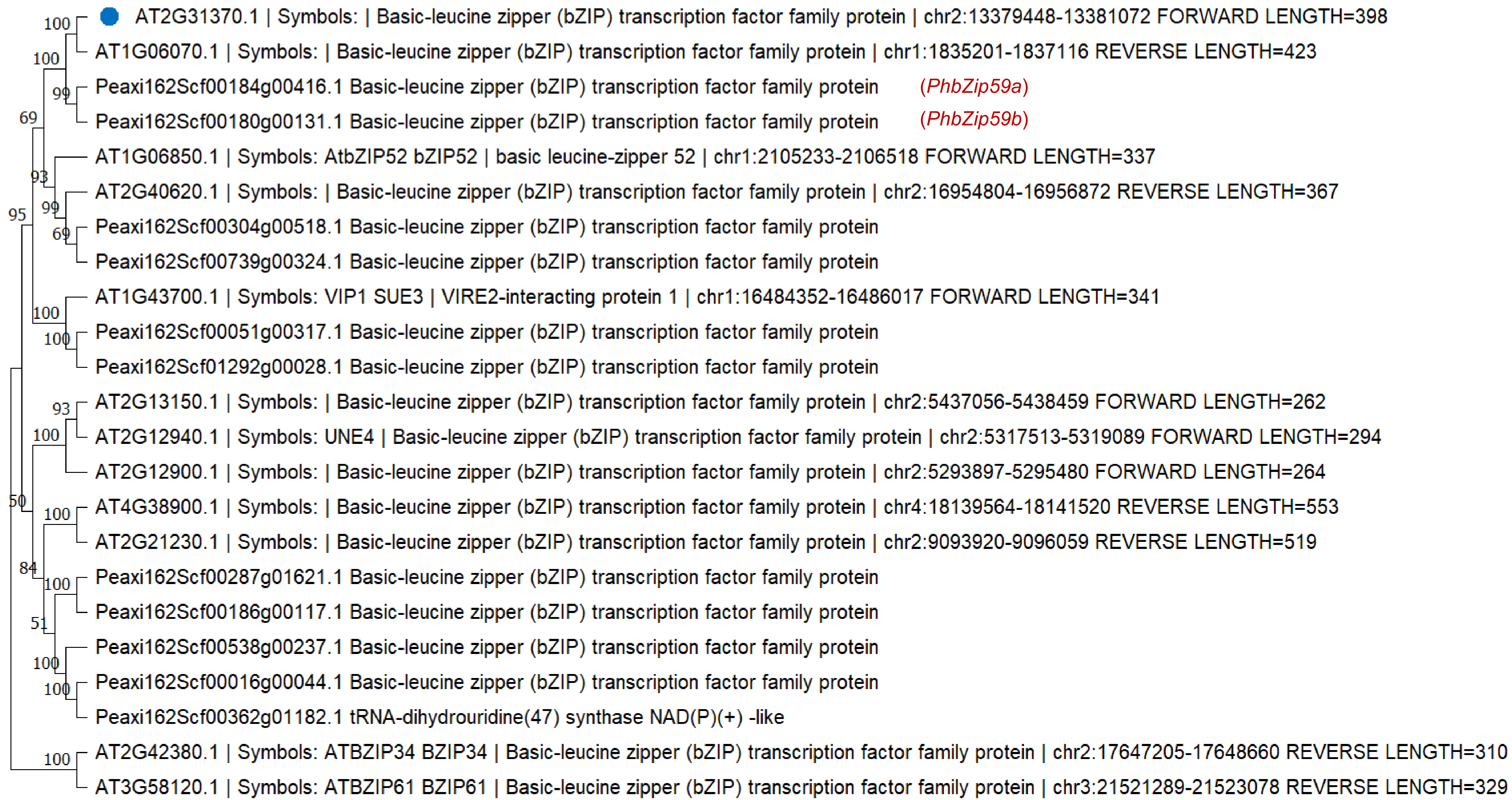
AT2G31370.1 | Symbols: | Basic-leucine zipper (bZIP) transcription factor family protein | chr2:13379448-13381072 FORWARD LENGTH=398
AT1G06070.1 | Symbols: | Basic-leucine zipper (bZIP) transcription factor family protein | chr1:1835201-1837116 REVERSE LENGTH=423
Peaxi162Scf00184g00416.1 Basic-leucine zipper (bZIP) transcription factor family protein
(PhbZip59a)
Peaxi162Scf00180g00131.1 Basic-leucine zipper (bZIP) transcription factor family protein
(PhbZip59b)
AT1G06850.1 | Symbols: AtbZIP52 bZIP52 | basic leucine-zipper 52 | chr1:2105233-2106518 FORWARD LENGTH=337
AT2G40620.1 | Symbols: | Basic-leucine zipper (bZIP) transcription factor family protein | chr2:16954804-16956872 REVERSE LENGTH=367
Peaxi162Scf00304g00518.1 Basic-leucine zipper (bZIP) transcription factor family protein
Peaxi162Scf00739g00324.1 Basic-leucine zipper (bZIP) transcription factor family protein
AT1G43700.1 | Symbols: VIP1 SUE3 | VIRE2-interacting protein 1 | chr1:16484352-16486017 FORWARD LENGTH=341
Peaxi162Scf00051g00317.1 Basic-leucine zipper (bZIP) transcription factor family protein
Peaxi162Scf01292g00028.1 Basic-leucine zipper (bZIP) transcription factor family protein
AT2G13150.1 | Symbols: | Basic-leucine zipper (bZIP) transcription factor family protein | chr2:5437056-5438459 FORWARD LENGTH=262
AT2G12940.1 | Symbols: UNE4 | Basic-leucine zipper (bZIP) transcription factor family protein | chr2:5317513-5319089 FORWARD LENGTH=294
AT2G12900.1 | Symbols: | Basic-leucine zipper (bZIP) transcription factor family protein | chr2:5293897-5295480 FORWARD LENGTH=264
AT4G38900.1 | Symbols: | Basic-leucine zipper (bZIP) transcription factor family protein | chr4:18139564-18141520 REVERSE LENGTH=553
AT2G21230.1 | Symbols: | Basic-leucine zipper (bZIP) transcription factor family protein | chr2:9093920-9096059 REVERSE LENGTH=519
Peaxi162Scf00287g01621.1 Basic-leucine zipper (bZIP) transcription factor family protein
Peaxi162Scf00186g00117.1 Basic-leucine zipper (bZIP) transcription factor family protein
Peaxi162Scf00538g00237.1 Basic-leucine zipper (bZIP) transcription factor family protein
Peaxi162Scf00016g00044.1 Basic-leucine zipper (bZIP) transcription factor family protein
Peaxi162Scf00362g01182.1 tRNA-dihydrouridine(47) synthase NAD(P)(+) -like
AT2G42380.1 | Symbols: ATBZIP34 BZIP34 | Basic-leucine zipper (bZIP) transcription factor family protein | chr2:17647205-17648660 REVERSE LENGTH=310
AT3G58120.1 | Symbols: ATBZIP61 BZIP61 | Basic-leucine zipper (bZIP) transcription factor family protein | chr3:21521289-21523078 REVERSE LENGTH=329

CYCD3

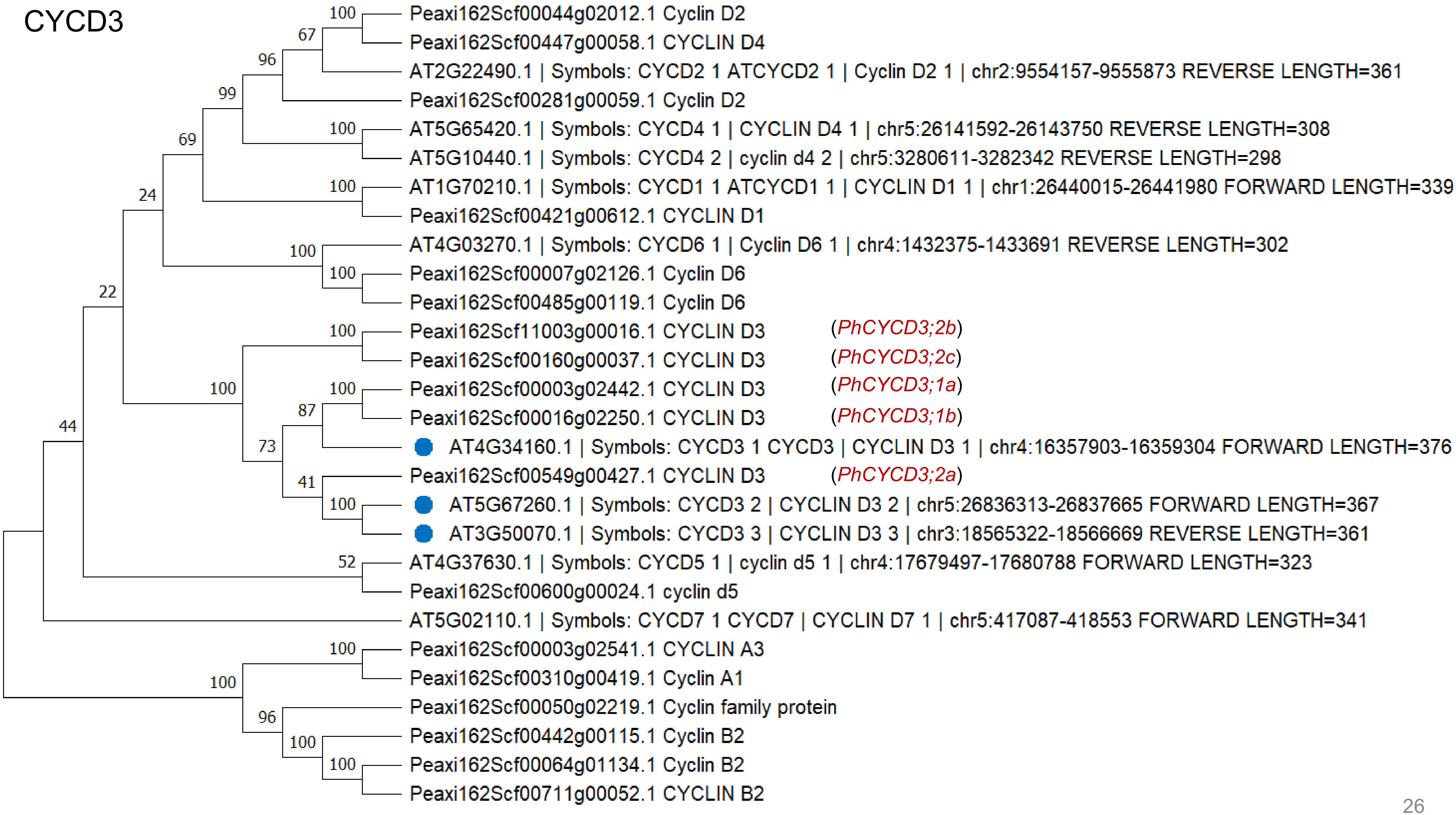

100
67
96
99
69
24
22
44
Peaxi162Scf00044g02012.1 Cyclin D2
Peaxi162Scf00447g00058.1 CYCLIN D4
AT2G22490.1 | Symbols: CYCD2 1 ATCYCD2 1 | Cyclin D2 1 | chr2:9554157-9555873 REVERSE LENGTH=361
Peaxi162Scf00281g00059.1 Cyclin D2
100
AT5G65420.1 | Symbols: CYCD4 1 | CYCLIN D4 1 | chr5:26141592-26143750 REVERSE LENGTH=308
AT5G10440.1 | Symbols: CYCD4 2 | cyclin d4 2 | chr5:3280611-3282342 REVERSE LENGTH=298
100
AT1G70210.1 | Symbols: CYCD1 1 ATCYCD1 1 | CYCLIN D1 1 | chr1:26440015-26441980 FORWARD LENGTH=339
Peaxi162Scf00421g00612.1 CYCLIN D1
100
AT4G03270.1 | Symbols: CYCD6 1 | Cyclin D6 1 | chr4:1432375-1433691 REVERSE LENGTH=302
100
Peaxi162Scf00007g02126.1 Cyclin D6
Peaxi162Scf00485g00119.1 Cyclin D6
100
Peaxi162Scf11003g00016.1 CYCLIN D3 (PhCYCD3;2b)
Peaxi162Scf00160g00037.1 CYCLIN D3 (PhCYCD3;2c)
100
100
87
73
Peaxi162Scf00003g02442.1 CYCLIN D3 (PhCYCD3;1a)
Peaxi162Scf00016g02250.1 CYCLIN D3 (PhCYCD3;1b)
AT4G34160.1 | Symbols: CYCD3 1 CYCD3 | CYCLIN D3 1 | chr4:16357903-16359304 FORWARD LENGTH=376
41
Peaxi162Scf00549g00427.1 CYCLIN D3 (PhCYCD3;2a)
100
AT5G67260.1 | Symbols: CYCD3 2 | CYCLIN D3 2 | chr5:26836313-26837665 FORWARD LENGTH=367
AT3G50070.1 | Symbols: CYCD3 3 | CYCLIN D3 3 | chr3:18565322-18566669 REVERSE LENGTH=361
52
AT4G37630.1 | Symbols: CYCD5 1 | cyclin d5 1 | chr4:17679497-17680788 FORWARD LENGTH=323
Peaxi162Scf00600g00024.1 cyclin d5
AT5G02110.1 | Symbols: CYCD7 1 CYCD7 | CYCLIN D7 1 | chr5:417087-418553 FORWARD LENGTH=341
100
100
Peaxi162Scf00003g02541.1 CYCLIN A3
Peaxi162Scf00310g00419.1 Cyclin A1
96
Peaxi162Scf00050g02219.1 Cyclin family protein
100
Peaxi162Scf00442g00115.1 Cyclin B2
100
Peaxi162Scf00064g01134.1 Cyclin B2
Peaxi162Scf00711g00052.1 CYCLIN B2

KMDs

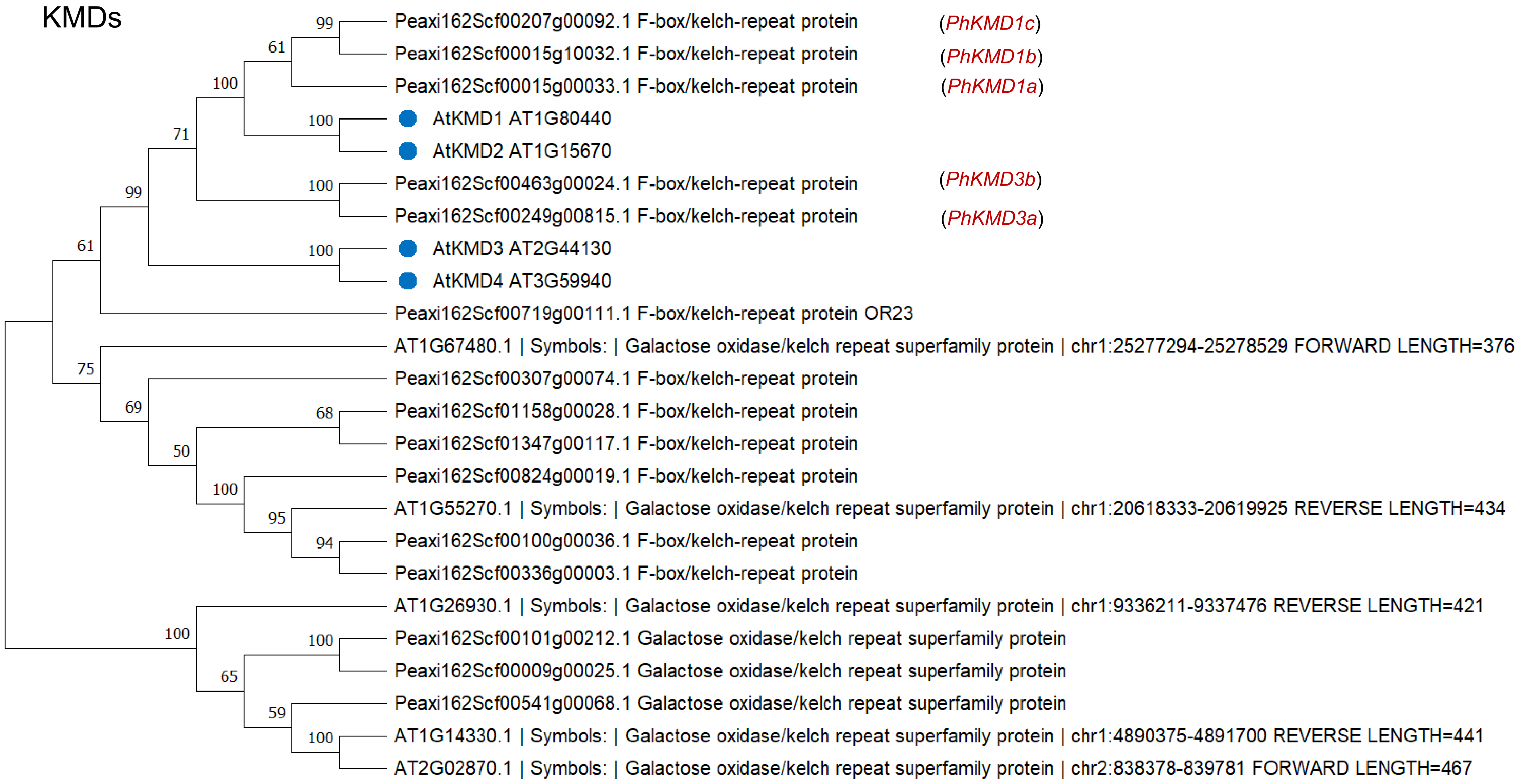

Peaxi162Scf00207g00092.1 F-box/kelch-repeat protein (PhKMD1c)
Peaxi162Scf00015g10032.1 F-box/kelch-repeat protein (PhKMD1b)
Peaxi162Scf00015g00033.1 F-box/kelch-repeat protein (PhKMD1a)
AtKMD1 AT1G80440
AtKMD2 AT1G15670
Peaxi162Scf00463g00024.1 F-box/kelch-repeat protein (PhKMD3b)
Peaxi162Scf00249g00815.1 F-box/kelch-repeat protein (PhKMD3a)
AtKMD3 AT2G44130
AtKMD4 AT3G59940
Peaxi162Scf00719g00111.1 F-box/kelch-repeat protein OR23
AT1G67480.1 | Symbols: | Galactose oxidase/kelch repeat superfamily protein | chr1:25277294-25278529 FORWARD LENGTH=376
Peaxi162Scf00307g00074.1 F-box/kelch-repeat protein
Peaxi162Scf01158g00028.1 F-box/kelch-repeat protein
Peaxi162Scf01347g00117.1 F-box/kelch-repeat protein
Peaxi162Scf00824g00019.1 F-box/kelch-repeat protein
AT1G55270.1 | Symbols: | Galactose oxidase/kelch repeat superfamily protein | chr1:20618333-20619925 REVERSE LENGTH=434
Peaxi162Scf00100g00036.1 F-box/kelch-repeat protein
Peaxi162Scf00336g00003.1 F-box/kelch-repeat protein
AT1G26930.1 | Symbols: | Galactose oxidase/kelch repeat superfamily protein | chr1:9336211-9337476 REVERSE LENGTH=421
Peaxi162Scf00101g00212.1 Galactose oxidase/kelch repeat superfamily protein
Peaxi162Scf00009g00025.1 Galactose oxidase/kelch repeat superfamily protein
Peaxi162Scf00541g00068.1 Galactose oxidase/kelch repeat superfamily protein
AT1G14330.1 | Symbols: | Galactose oxidase/kelch repeat superfamily protein | chr1:4890375-4891700 REVERSE LENGTH=441
AT2G02870.1 | Symbols: | Galactose oxidase/kelch repeat superfamily protein | chr2:838378-839781 FORWARD LENGTH=467
99
61
100
100
71
99
100
61
100
75
69
68
50
100
95
94
100
100
65
59
100

CA2

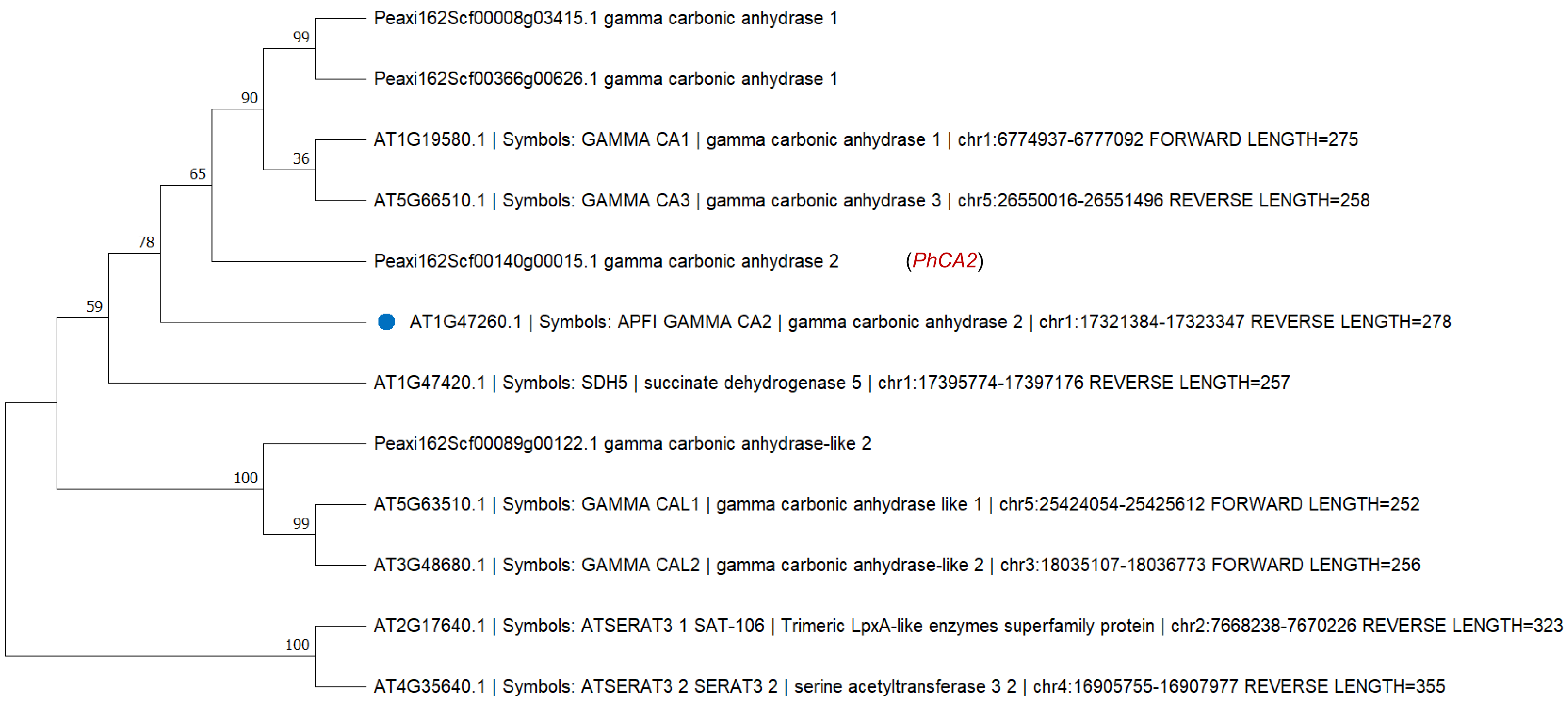
Peaxi162Scf00008g03415.1 gamma carbonic anhydrase 1
Peaxi162Scf00366g00626.1 gamma carbonic anhydrase 1
AT1G19580.1 | Symbols: GAMMA CA1 | gamma carbonic anhydrase 1 | chr1:6774937-6777092 FORWARD LENGTH=275
AT5G66510.1 | Symbols: GAMMA CA3 | gamma carbonic anhydrase 3 | chr5:26550016-26551496 REVERSE LENGTH=258
Peaxi162Scf00140g00015.1 gamma carbonic anhydrase 2
(PhCA2)
AT1G47260.1 | Symbols: APFI GAMMA CA2 | gamma carbonic anhydrase 2 | chr1:17321384-17323347 REVERSE LENGTH=278
AT1G47420.1 | Symbols: SDH5 | succinate dehydrogenase 5 | chr1:17395774-17397176 REVERSE LENGTH=257
Peaxi162Scf00089g00122.1 gamma carbonic anhydrase-like 2
AT5G63510.1 | Symbols: GAMMA CAL1 | gamma carbonic anhydrase like 1 | chr5:25424054-25425612 FORWARD LENGTH=252
AT3G48680.1 | Symbols: GAMMA CAL2 | gamma carbonic anhydrase-like 2 | chr3:18035107-18036773 FORWARD LENGTH=256
AT2G17640.1 | Symbols: ATSERAT3 1 SAT-106 | Trimeric LpxA-like enzymes superfamily protein | chr2:7668238-7670226 REVERSE LENGTH=323
AT4G35640.1 | Symbols: ATSERAT3 2 SERAT3 2 | serine acetyltransferase 3 2 | chr4:16905755-16907977 REVERSE LENGTH=355
99
90
36
65
78
59
100
99
100

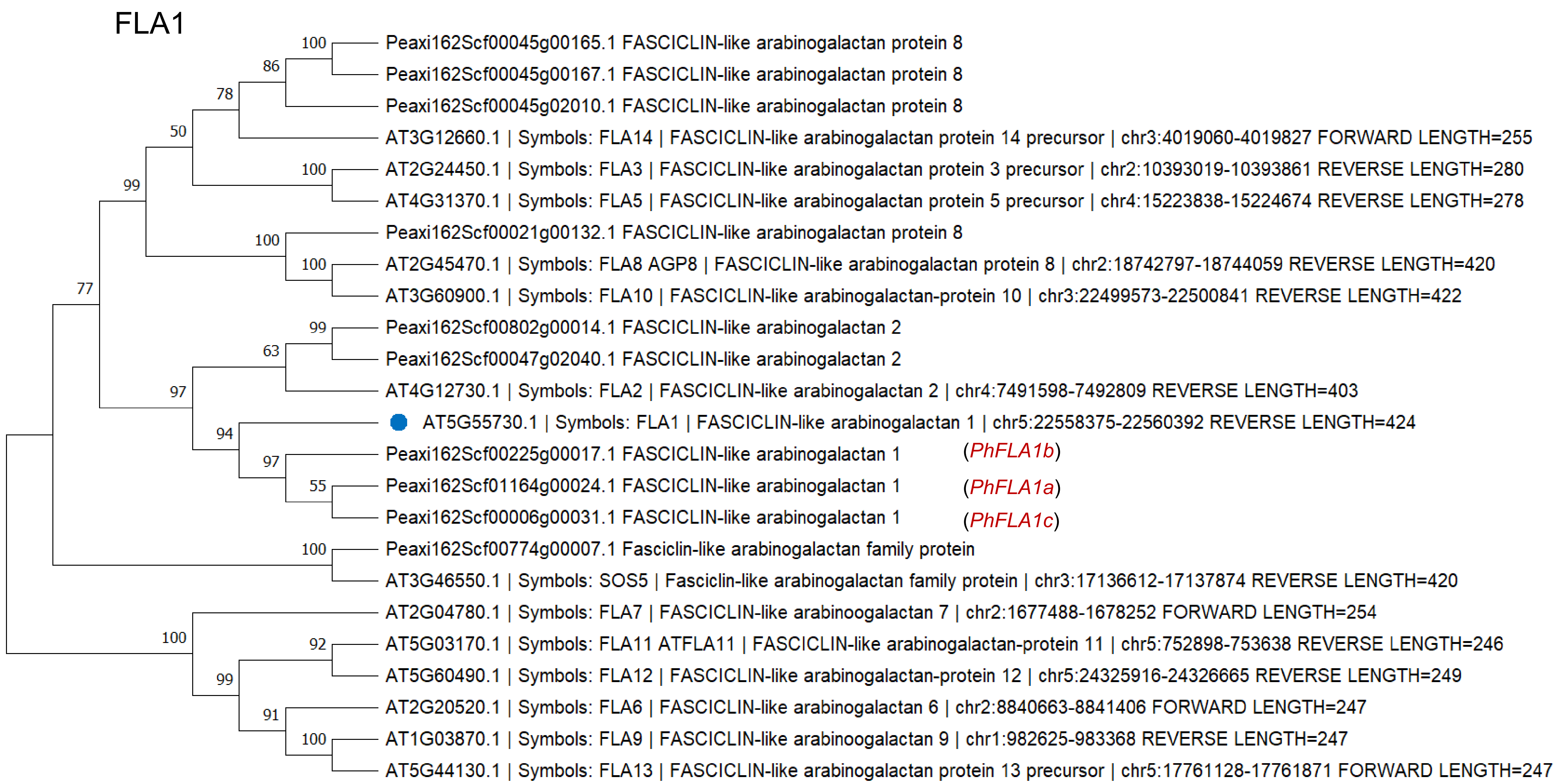
FLA1
Peaxi162Scf00045g00165.1 FASCICLIN-like arabinogalactan protein 8
Peaxi162Scf00045g00167.1 FASCICLIN-like arabinogalactan protein 8
Peaxi162Scf00045g02010.1 FASCICLIN-like arabinogalactan protein 8
AT3G12660.1 | Symbols: FLA14 | FASCICLIN-like arabinogalactan protein 14 precursor | chr3:4019060-4019827 FORWARD LENGTH=255
AT2G24450.1 | Symbols: FLA3 | FASCICLIN-like arabinogalactan protein 3 precursor | chr2:10393019-10393861 REVERSE LENGTH=280
AT4G31370.1 | Symbols: FLA5 | FASCICLIN-like arabinogalactan protein 5 precursor | chr4:15223838-15224674 REVERSE LENGTH=278
Peaxi162Scf00021g00132.1 FASCICLIN-like arabinogalactan protein 8
AT2G45470.1 | Symbols: FLA8 AGP8 | FASCICLIN-like arabinogalactan protein 8 | chr2:18742797-18744059 REVERSE LENGTH=420
AT3G60900.1 | Symbols: FLA10 | FASCICLIN-like arabinogalactan-protein 10 | chr3:22499573-22500841 REVERSE LENGTH=422
Peaxi162Scf00802g00014.1 FASCICLIN-like arabinogalactan 2
Peaxi162Scf00047g02040.1 FASCICLIN-like arabinogalactan 2
AT4G12730.1 | Symbols: FLA2 | FASCICLIN-like arabinogalactan 2 | chr4:7491598-7492809 REVERSE LENGTH=403
AT5G55730.1 | Symbols: FLA1 | FASCICLIN-like arabinogalactan 1 | chr5:22558375-22560392 REVERSE LENGTH=424
Peaxi162Scf00225g00017.1 FASCICLIN-like arabinogalactan 1 (PhFLA1b)
Peaxi162Scf01164g00024.1 FASCICLIN-like arabinogalactan 1 (PhFLA1a)
Peaxi162Scf00006g00031.1 FASCICLIN-like arabinogalactan 1 (PhFLA1c)
Peaxi162Scf00774g00007.1 Fasciclin-like arabinogalactan family protein
AT3G46550.1 | Symbols: SOS5 | Fasciclin-like arabinogalactan family protein | chr3:17136612-17137874 REVERSE LENGTH=420
AT2G04780.1 | Symbols: FLA7 | FASCICLIN-like arabinoogalactan 7 | chr2:1677488-1678252 FORWARD LENGTH=254
AT5G03170.1 | Symbols: FLA11 ATFLA11 | FASCICLIN-like arabinogalactan-protein 11 | chr5:752898-753638 REVERSE LENGTH=246
AT5G60490.1 | Symbols: FLA12 | FASCICLIN-like arabinogalactan-protein 12 | chr5:24325916-24326665 REVERSE LENGTH=249
AT2G20520.1 | Symbols: FLA6 | FASCICLIN-like arabinogalactan 6 | chr2:8840663-8841406 FORWARD LENGTH=247
AT1G03870.1 | Symbols: FLA9 | FASCICLIN-like arabinoogalactan 9 | chr1:982625-983368 REVERSE LENGTH=247
AT5G44130.1 | Symbols: FLA13 | FASCICLIN-like arabinogalactan protein 13 precursor | chr5:17761128-17761871 FORWARD LENGTH=247

SIZ1

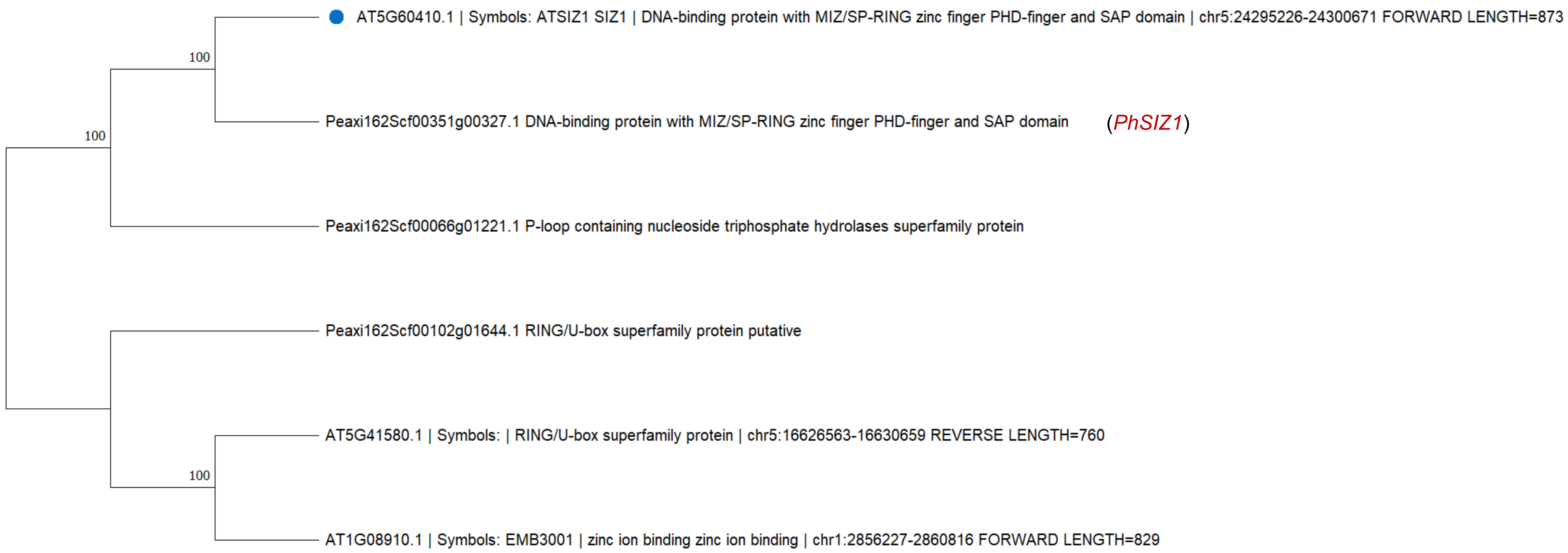
AT5G60410.1 | Symbols: ATSIZ1 SIZ1 | DNA-binding protein with MIZ/SP-RING zinc finger PHD-finger and SAP domain | chr5:24295226-24300671 FORWARD LENGTH=873
100
Peaxi162Scf00351g00327.1 DNA-binding protein with MIZ/SP-RING zinc finger PHD-finger and SAP domain
(PhSIZ1)
100
Peaxi162Scf00066g01221.1 P-loop containing nucleoside triphosphate hydrolases superfamily protein
Peaxi162Scf00102g01644.1 RING/U-box superfamily protein putative
AT5G41580.1 | Symbols: | RING/U-box superfamily protein | chr5:16626563-16630659 REVERSE LENGTH=760
100
AT1G08910.1 | Symbols: EMB3001 | zinc ion binding zinc ion binding | chr1:2856227-2860816 FORWARD LENGTH=829

CEPs

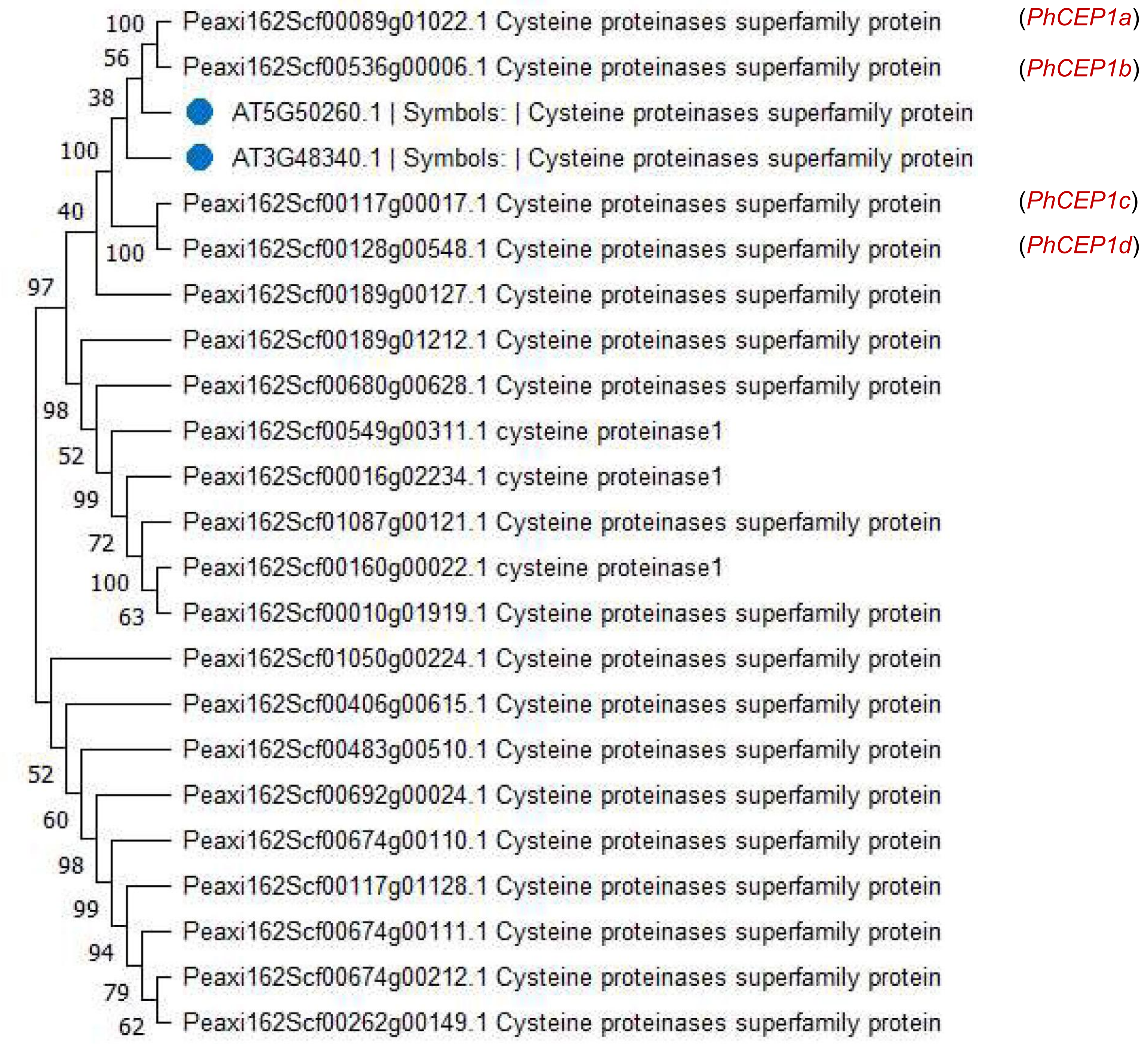
Peaxi162Scf00089g01022.1 Cysteine proteinases superfamily protein
(PhCEP1a)
Peaxi162Scf00536g00006.1 Cysteine proteinases superfamily protein
(PhCEP1b)
AT5G50260.1 | Symbols: | Cysteine proteinases superfamily protein
AT3G48340.1 | Symbols: | Cysteine proteinases superfamily protein
Peaxi162Scf00117g00017.1 Cysteine proteinases superfamily protein
(PhCEP1c)
Peaxi162Scf00128g00548.1 Cysteine proteinases superfamily protein
(PhCEP1d)
Peaxi162Scf00189g00127.1 Cysteine proteinases superfamily protein
Peaxi162Scf00189g01212.1 Cysteine proteinases superfamily protein
Peaxi162Scf00680g00628.1 Cysteine proteinases superfamily protein
Peaxi162Scf00549g00311.1 cysteine proteinase1
Peaxi162Scf00016g02234.1 cysteine proteinase1
Peaxi162Scf01087g00121.1 Cysteine proteinases superfamily protein
Peaxi162Scf00160g00022.1 cysteine proteinase1
Peaxi162Scf00010g01919.1 Cysteine proteinases superfamily protein
Peaxi162Scf01050g00224.1 Cysteine proteinases superfamily protein
Peaxi162Scf00406g00615.1 Cysteine proteinases superfamily protein
Peaxi162Scf00483g00510.1 Cysteine proteinases superfamily protein
Peaxi162Scf00692g00024.1 Cysteine proteinases superfamily protein
Peaxi162Scf00674g00110.1 Cysteine proteinases superfamily protein
Peaxi162Scf00117g01128.1 Cysteine proteinases superfamily protein
Peaxi162Scf00674g00111.1 Cysteine proteinases superfamily protein
Peaxi162Scf00674g00212.1 Cysteine proteinases superfamily protein
Peaxi162Scf00262g00149.1 Cysteine proteinases superfamily protein
100
56
38
100
40
100
97
98
52
99
72
100
63
52
60
98
99
94
79
62

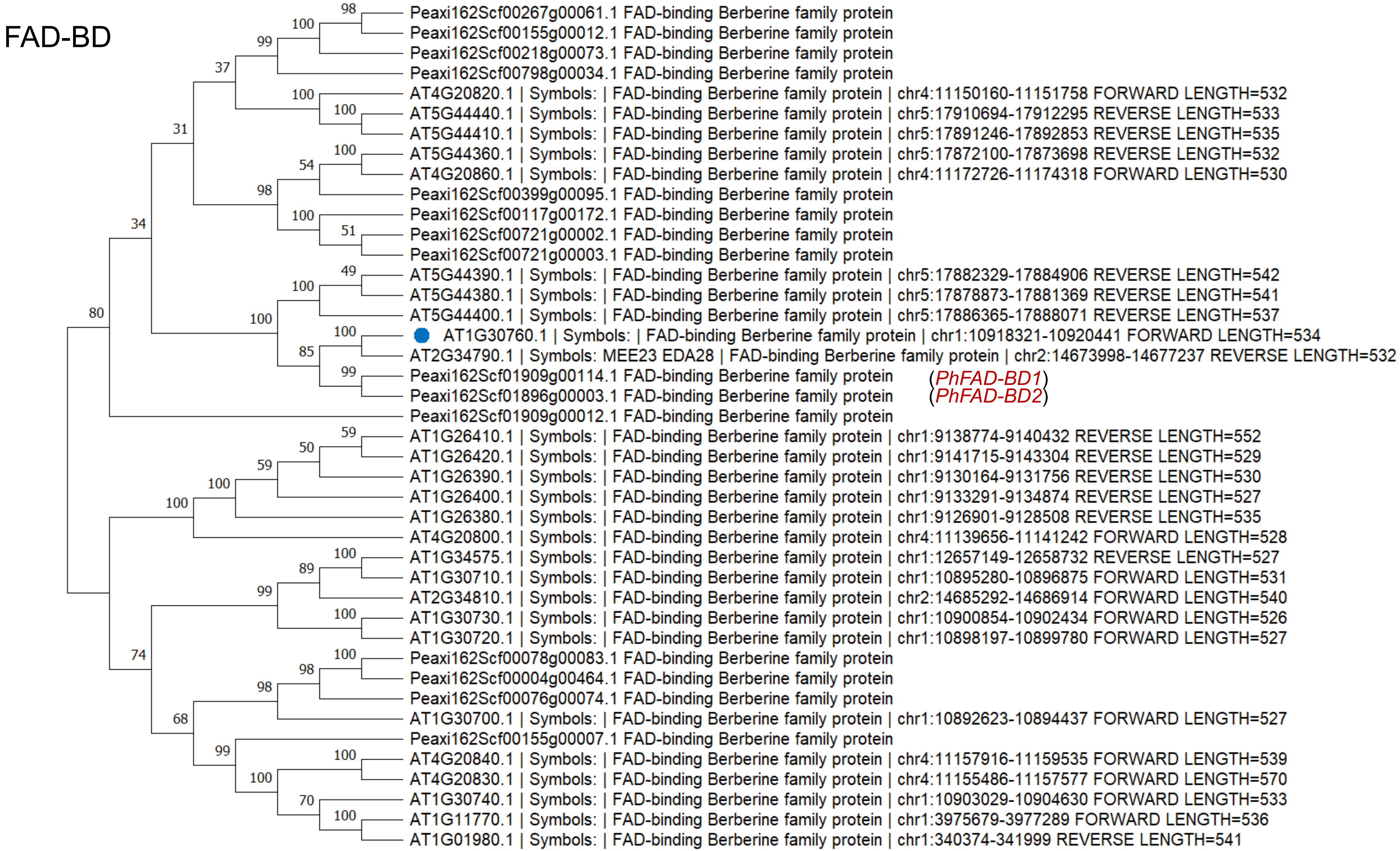

FAD-BD
Peaxi162Scf00267g00061.1 FAD-binding Berberine family protein
Peaxi162Scf00155g00012.1 FAD-binding Berberine family protein
Peaxi162Scf00218g00073.1 FAD-binding Berberine family protein
Peaxi162Scf00798g00034.1 FAD-binding Berberine family protein
AT4G20820.1 | Symbols: | FAD-binding Berberine family protein | chr4:11150160-11151758 FORWARD LENGTH=532
AT5G44440.1 | Symbols: | FAD-binding Berberine family protein | chr5:17910694-17912295 REVERSE LENGTH=533
AT5G44410.1 | Symbols: | FAD-binding Berberine family protein | chr5:17891246-17892853 REVERSE LENGTH=535
AT5G44360.1 | Symbols: | FAD-binding Berberine family protein | chr5:17872100-17873698 REVERSE LENGTH=532
AT4G20860.1 | Symbols: | FAD-binding Berberine family protein | chr4:11172726-11174318 FORWARD LENGTH=530
Peaxi162Scf00399g00095.1 FAD-binding Berberine family protein
Peaxi162Scf00117g00172.1 FAD-binding Berberine family protein
Peaxi162Scf00721g00002.1 FAD-binding Berberine family protein
Peaxi162Scf00721g00003.1 FAD-binding Berberine family protein
AT5G44390.1 | Symbols: | FAD-binding Berberine family protein | chr5:17882329-17884906 REVERSE LENGTH=542
AT5G44380.1 | Symbols: | FAD-binding Berberine family protein | chr5:17878873-17881369 REVERSE LENGTH=541
AT5G44400.1 | Symbols: | FAD-binding Berberine family protein | chr5:17886365-17888071 REVERSE LENGTH=537
AT1G30760.1 | Symbols: | FAD-binding Berberine family protein | chr1:10918321-10920441 FORWARD LENGTH=534
AT2G34790.1 | Symbols: MEE23 EDA28 | FAD-binding Berberine family protein | chr2:14673998-14677237 REVERSE LENGTH=532
Peaxi162Scf01909g00114.1 FAD-binding Berberine family protein (PhFAD-BD1)
Peaxi162Scf01896g00003.1 FAD-binding Berberine family protein (PhFAD-BD2)
Peaxi162Scf01909g00012.1 FAD-binding Berberine family protein
AT1G26410.1 | Symbols: | FAD-binding Berberine family protein | chr1:9138774-9140432 REVERSE LENGTH=552
AT1G26420.1 | Symbols: | FAD-binding Berberine family protein | chr1:9141715-9143304 REVERSE LENGTH=529
AT1G26390.1 | Symbols: | FAD-binding Berberine family protein | chr1:9130164-9131756 REVERSE LENGTH=530
AT1G26400.1 | Symbols: | FAD-binding Berberine family protein | chr1:9133291-9134874 REVERSE LENGTH=527
AT1G26380.1 | Symbols: | FAD-binding Berberine family protein | chr1:9126901-9128508 REVERSE LENGTH=535
AT4G20800.1 | Symbols: | FAD-binding Berberine family protein | chr4:11139656-11141242 FORWARD LENGTH=528
AT1G34575.1 | Symbols: | FAD-binding Berberine family protein | chr1:12657149-12658732 REVERSE LENGTH=527
AT1G30710.1 | Symbols: | FAD-binding Berberine family protein | chr1:10895280-10896875 FORWARD LENGTH=531
AT2G34810.1 | Symbols: | FAD-binding Berberine family protein | chr2:14685292-14686914 FORWARD LENGTH=540
AT1G30730.1 | Symbols: | FAD-binding Berberine family protein | chr1:10900854-10902434 FORWARD LENGTH=526
AT1G30720.1 | Symbols: | FAD-binding Berberine family protein | chr1:10898197-10899780 FORWARD LENGTH=527
Peaxi162Scf00078g00083.1 FAD-binding Berberine family protein
Peaxi162Scf00004g00464.1 FAD-binding Berberine family protein
Peaxi162Scf00076g00074.1 FAD-binding Berberine family protein
AT1G30700.1 | Symbols: | FAD-binding Berberine family protein | chr1:10892623-10894437 FORWARD LENGTH=527
Peaxi162Scf00155g00007.1 FAD-binding Berberine family protein
AT4G20840.1 | Symbols: | FAD-binding Berberine family protein | chr4:11157916-11159535 FORWARD LENGTH=539
AT4G20830.1 | Symbols: | FAD-binding Berberine family protein | chr4:11155486-11157577 FORWARD LENGTH=570
AT1G30740.1 | Symbols: | FAD-binding Berberine family protein | chr1:10903029-10904630 FORWARD LENGTH=533
AT1G11770.1 | Symbols: | FAD-binding Berberine family protein | chr1:3975679-3977289 FORWARD LENGTH=536
AT1G01980.1 | Symbols: | FAD-binding Berberine family protein | chr1:340374-341999 REVERSE LENGTH=541

KCS1

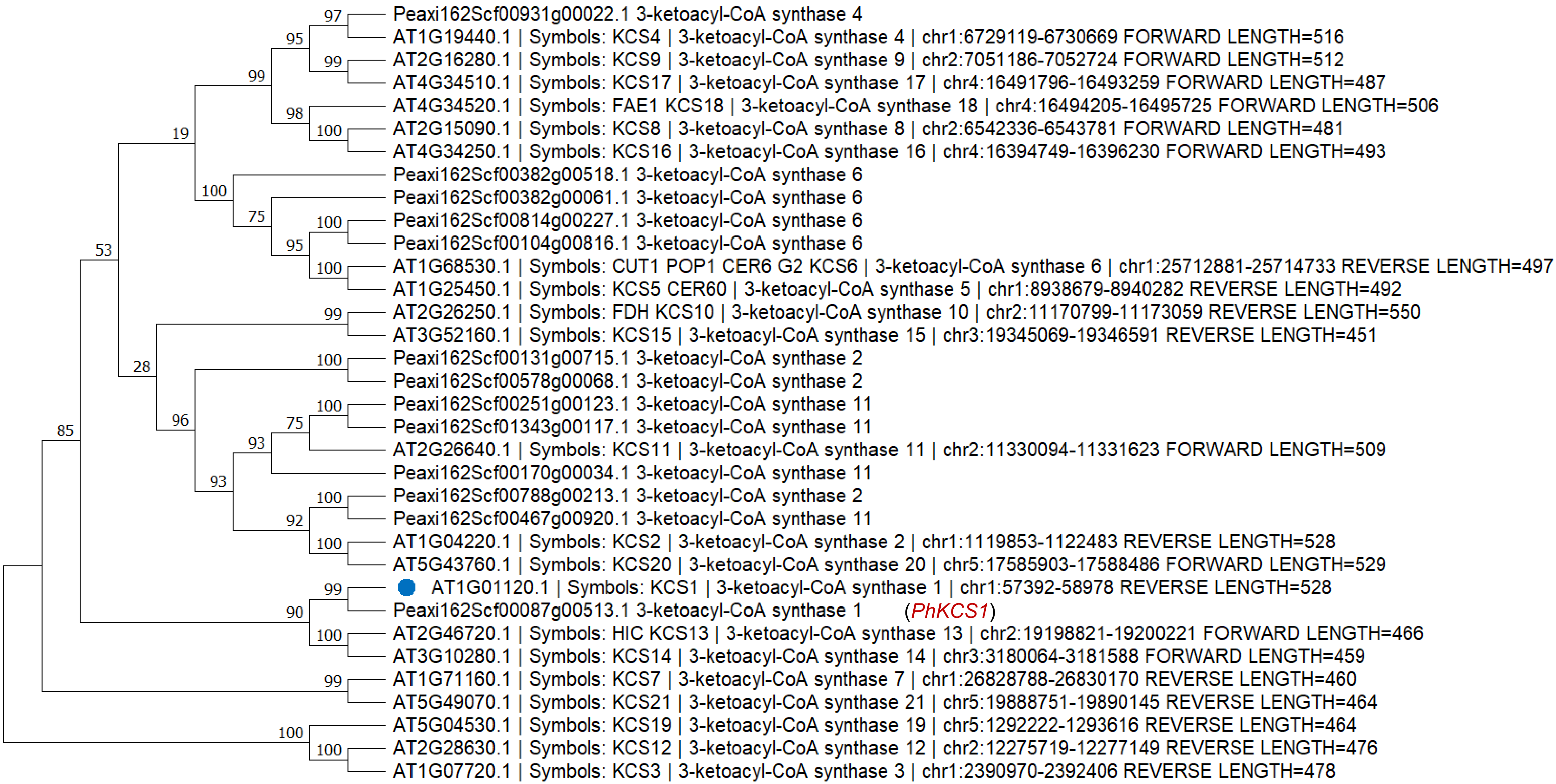
Peaxi162Scf00931g00022.1 3-ketoacyl-CoA synthase 4
AT1G19440.1 | Symbols: KCS4 | 3-ketoacyl-CoA synthase 4 | chr1:6729119-6730669 FORWARD LENGTH=516
AT2G16280.1 | Symbols: KCS9 | 3-ketoacyl-CoA synthase 9 | chr2:7051186-7052724 FORWARD LENGTH=512
AT4G34510.1 | Symbols: KCS17 | 3-ketoacyl-CoA synthase 17 | chr4:16491796-16493259 FORWARD LENGTH=487
AT4G34520.1 | Symbols: FAE1 KCS18 | 3-ketoacyl-CoA synthase 18 | chr4:16494205-16495725 FORWARD LENGTH=506
AT2G15090.1 | Symbols: KCS8 | 3-ketoacyl-CoA synthase 8 | chr2:6542336-6543781 FORWARD LENGTH=481
AT4G34250.1 | Symbols: KCS16 | 3-ketoacyl-CoA synthase 16 | chr4:16394749-16396230 FORWARD LENGTH=493
Peaxi162Scf00382g00518.1 3-ketoacyl-CoA synthase 6
Peaxi162Scf00382g00061.1 3-ketoacyl-CoA synthase 6
Peaxi162Scf00814g00227.1 3-ketoacyl-CoA synthase 6
Peaxi162Scf00104g00816.1 3-ketoacyl-CoA synthase 6
AT1G68530.1 | Symbols: CUT1 POP1 CER6 G2 KCS6 | 3-ketoacyl-CoA synthase 6 | chr1:25712881-25714733 REVERSE LENGTH=497
AT1G25450.1 | Symbols: KCS5 CER60 | 3-ketoacyl-CoA synthase 5 | chr1:8938679-8940282 REVERSE LENGTH=492
AT2G26250.1 | Symbols: FDH KCS10 | 3-ketoacyl-CoA synthase 10 | chr2:11170799-11173059 REVERSE LENGTH=550
AT3G52160.1 | Symbols: KCS15 | 3-ketoacyl-CoA synthase 15 | chr3:19345069-19346591 REVERSE LENGTH=451
Peaxi162Scf00131g00715.1 3-ketoacyl-CoA synthase 2
Peaxi162Scf00578g00068.1 3-ketoacyl-CoA synthase 2
Peaxi162Scf00251g00123.1 3-ketoacyl-CoA synthase 11
Peaxi162Scf01343g00117.1 3-ketoacyl-CoA synthase 11
AT2G26640.1 | Symbols: KCS11 | 3-ketoacyl-CoA synthase 11 | chr2:11330094-11331623 FORWARD LENGTH=509
Peaxi162Scf00170g00034.1 3-ketoacyl-CoA synthase 11
Peaxi162Scf00788g00213.1 3-ketoacyl-CoA synthase 2
Peaxi162Scf00467g00920.1 3-ketoacyl-CoA synthase 11
AT1G04220.1 | Symbols: KCS2 | 3-ketoacyl-CoA synthase 2 | chr1:1119853-1122483 REVERSE LENGTH=528
AT5G43760.1 | Symbols: KCS20 | 3-ketoacyl-CoA synthase 20 | chr5:17585903-17588486 FORWARD LENGTH=529
AT1G01120.1 | Symbols: KCS1 | 3-ketoacyl-CoA synthase 1 | chr1:57392-58978 REVERSE LENGTH=528
Peaxi162Scf00087g00513.1 3-ketoacyl-CoA synthase 1 (PhKCS1)
AT2G46720.1 | Symbols: HIC KCS13 | 3-ketoacyl-CoA synthase 13 | chr2:19198821-19200221 FORWARD LENGTH=466
AT3G10280.1 | Symbols: KCS14 | 3-ketoacyl-CoA synthase 14 | chr3:3180064-3181588 FORWARD LENGTH=459
AT1G71160.1 | Symbols: KCS7 | 3-ketoacyl-CoA synthase 7 | chr1:26828788-26830170 REVERSE LENGTH=460
AT5G49070.1 | Symbols: KCS21 | 3-ketoacyl-CoA synthase 21 | chr5:19888751-19890145 REVERSE LENGTH=464
AT5G04530.1 | Symbols: KCS19 | 3-ketoacyl-CoA synthase 19 | chr5:1292222-1293616 REVERSE LENGTH=464
AT2G28630.1 | Symbols: KCS12 | 3-ketoacyl-CoA synthase 12 | chr2:12275719-12277149 REVERSE LENGTH=476
AT1G07720.1 | Symbols: KCS3 | 3-ketoacyl-CoA synthase 3 | chr1:2390970-2392406 REVERSE LENGTH=478

MSLs

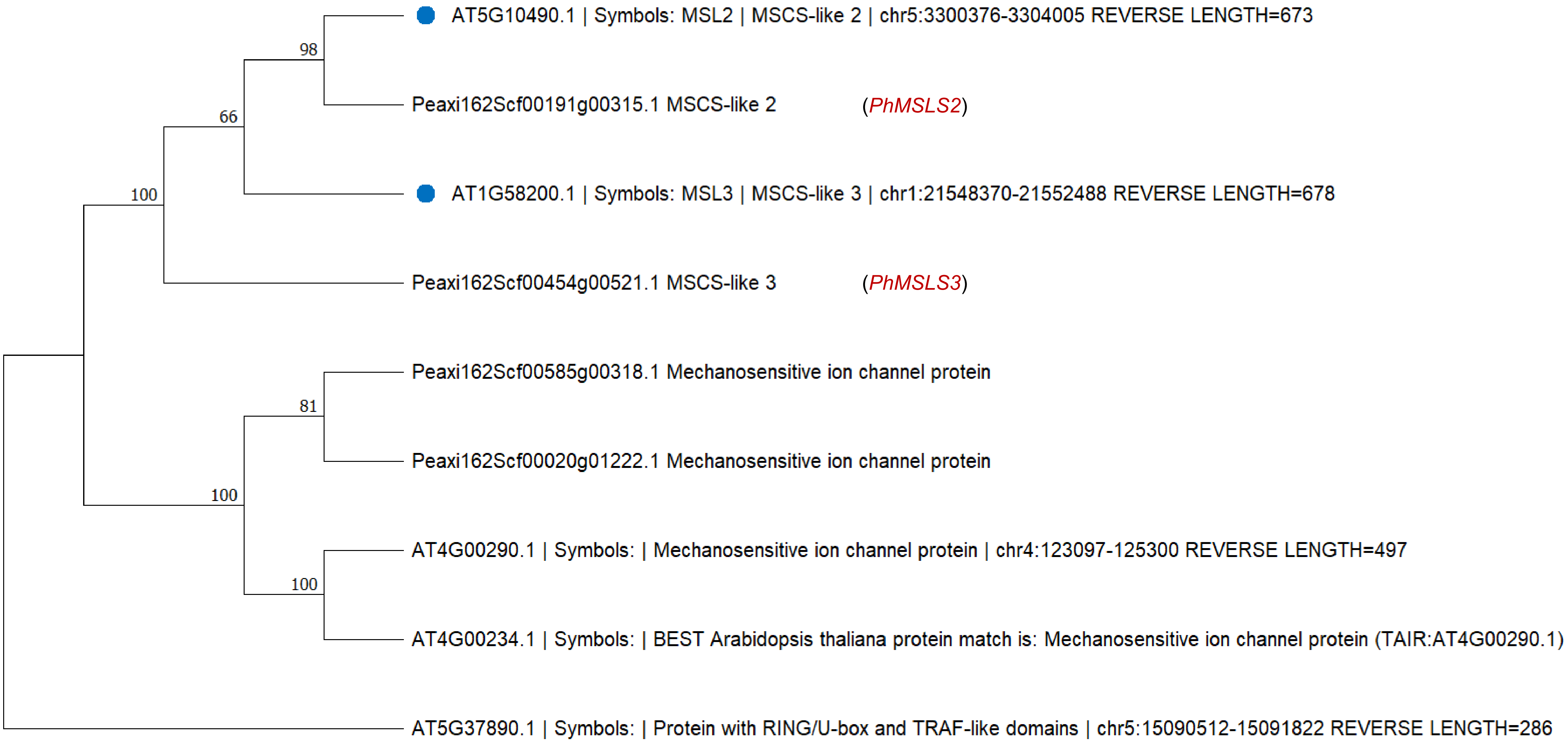
AT5G10490.1 | Symbols: MSL2 | MSCS-like 2 | chr5:3300376-3304005 REVERSE LENGTH=673
Peaxi162Scf00191g00315.1 MSCS-like 2
(PhMSLS2)
AT1G58200.1 | Symbols: MSL3 | MSCS-like 3 | chr1:21548370-21552488 REVERSE LENGTH=678
Peaxi162Scf00454g00521.1 MSCS-like 3
(PhMSLS3)
Peaxi162Scf00585g00318.1 Mechanosensitive ion channel protein
Peaxi162Scf00020g01222.1 Mechanosensitive ion channel protein
AT4G00290.1 | Symbols: | Mechanosensitive ion channel protein | chr4:123097-125300 REVERSE LENGTH=497
AT4G00234.1 | Symbols: | BEST Arabidopsis thaliana protein match is: Mechanosensitive ion channel protein (TAIR:AT4G00290.1)
AT5G37890.1 | Symbols: | Protein with RING/U-box and TRAF-like domains | chr5:15090512-15091822 REVERSE LENGTH=286
98
66
100
81
100
100

PME2

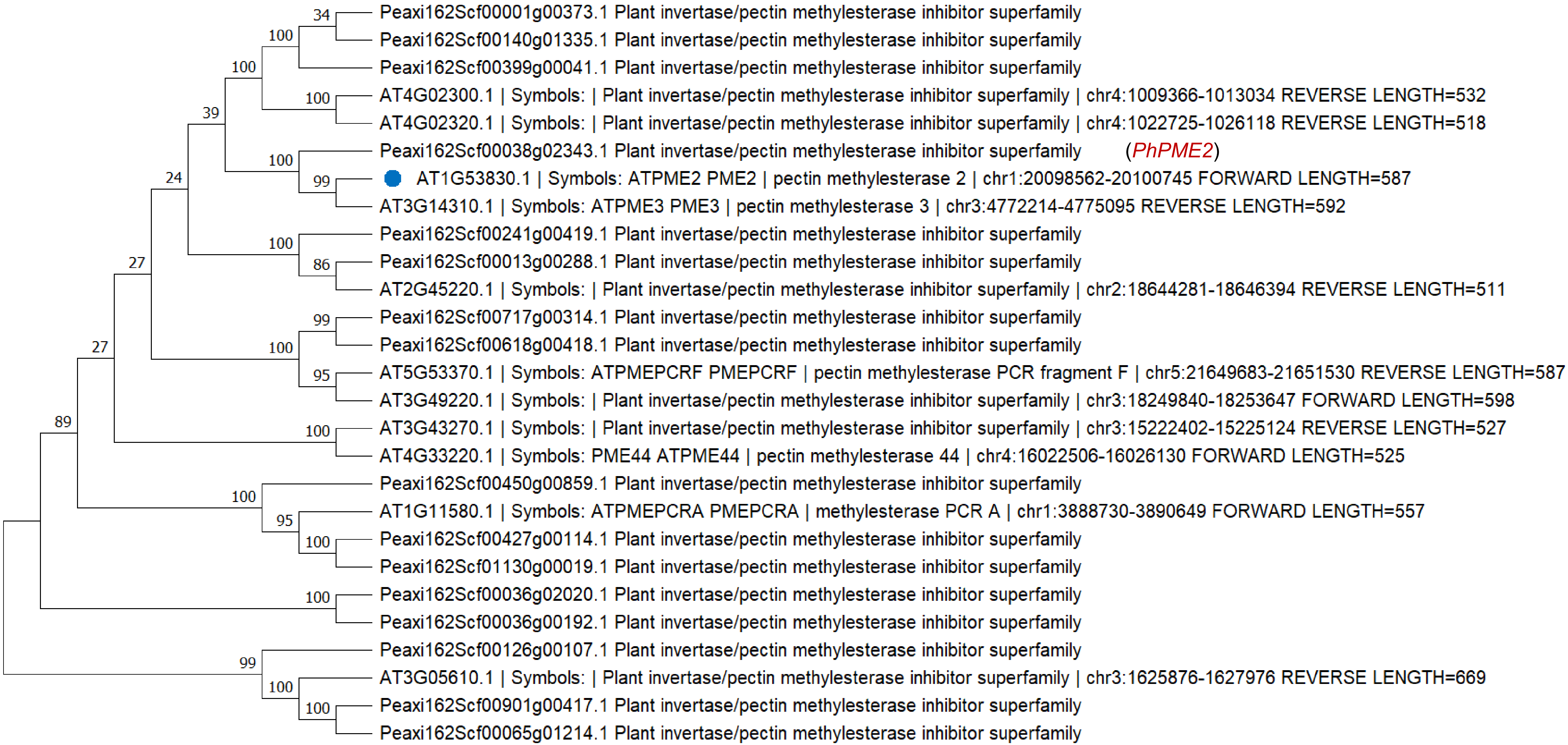

Peaxi162Scf00001g00373.1 Plant invertase/pectin methylesterase inhibitor superfamily
Peaxi162Scf00140g01335.1 Plant invertase/pectin methylesterase inhibitor superfamily
Peaxi162Scf00399g00041.1 Plant invertase/pectin methylesterase inhibitor superfamily
AT4G02300.1 | Symbols: | Plant invertase/pectin methylesterase inhibitor superfamily | chr4:1009366-1013034 REVERSE LENGTH=532
AT4G02320.1 | Symbols: | Plant invertase/pectin methylesterase inhibitor superfamily | chr4:1022725-1026118 REVERSE LENGTH=518
Peaxi162Scf00038g02343.1 Plant invertase/pectin methylesterase inhibitor superfamily
(*PhPME2*)
AT1G53830.1 | Symbols: ATPME2 PME2 | pectin methylesterase 2 | chr1:20098562-20100745 FORWARD LENGTH=587
AT3G14310.1 | Symbols: ATPME3 PME3 | pectin methylesterase 3 | chr3:4772214-4775095 REVERSE LENGTH=592
Peaxi162Scf00241g00419.1 Plant invertase/pectin methylesterase inhibitor superfamily
Peaxi162Scf00013g00288.1 Plant invertase/pectin methylesterase inhibitor superfamily
AT2G45220.1 | Symbols: | Plant invertase/pectin methylesterase inhibitor superfamily | chr2:18644281-18646394 REVERSE LENGTH=511
Peaxi162Scf00717g00314.1 Plant invertase/pectin methylesterase inhibitor superfamily
Peaxi162Scf00618g00418.1 Plant invertase/pectin methylesterase inhibitor superfamily
AT5G53370.1 | Symbols: ATPMEPCRF PMEPCRF | pectin methylesterase PCR fragment F | chr5:21649683-21651530 REVERSE LENGTH=587
AT3G49220.1 | Symbols: | Plant invertase/pectin methylesterase inhibitor superfamily | chr3:18249840-18253647 FORWARD LENGTH=598
AT3G43270.1 | Symbols: | Plant invertase/pectin methylesterase inhibitor superfamily | chr3:15222402-15225124 REVERSE LENGTH=527
AT4G33220.1 | Symbols: PME44 ATPME44 | pectin methylesterase 44 | chr4:16022506-16026130 FORWARD LENGTH=525
Peaxi162Scf00450g00859.1 Plant invertase/pectin methylesterase inhibitor superfamily
AT1G11580.1 | Symbols: ATPMEPCRA PMEPCRA | methylesterase PCR A | chr1:3888730-3890649 FORWARD LENGTH=557
Peaxi162Scf00427g00114.1 Plant invertase/pectin methylesterase inhibitor superfamily
Peaxi162Scf01130g00019.1 Plant invertase/pectin methylesterase inhibitor superfamily
Peaxi162Scf00036g02020.1 Plant invertase/pectin methylesterase inhibitor superfamily
Peaxi162Scf00036g00192.1 Plant invertase/pectin methylesterase inhibitor superfamily
Peaxi162Scf00126g00107.1 Plant invertase/pectin methylesterase inhibitor superfamily
AT3G05610.1 | Symbols: | Plant invertase/pectin methylesterase inhibitor superfamily | chr3:1625876-1627976 REVERSE LENGTH=669
Peaxi162Scf00901g00417.1 Plant invertase/pectin methylesterase inhibitor superfamily
Peaxi162Scf00065g01214.1 Plant invertase/pectin methylesterase inhibitor superfamily
34
100
100
100
39
100
99
24
100
86
27
99
100
27
95
89
100
100
95
100
100
99
100
100

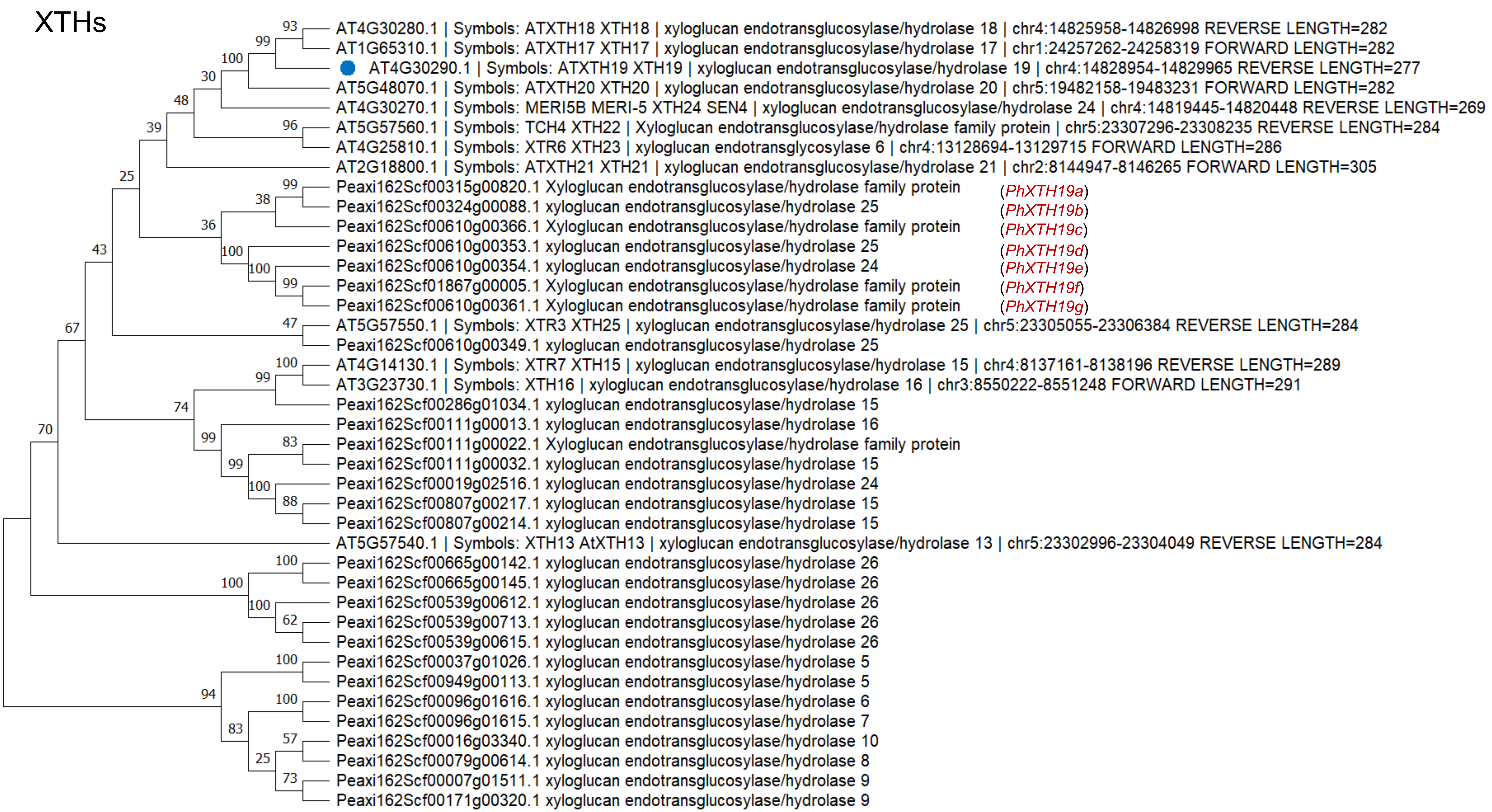
XTHs
AT4G30280.1 | Symbols: ATXTH18 XTH18 | xyloglucan endotransglucosylase/hydrolase 18 | chr4:14825958-14826998 REVERSE LENGTH=282
AT1G65310.1 | Symbols: ATXTH17 XTH17 | xyloglucan endotransglucosylase/hydrolase 17 | chr1:24257262-24258319 FORWARD LENGTH=282
AT4G30290.1 | Symbols: ATXTH19 XTH19 | xyloglucan endotransglucosylase/hydrolase 19 | chr4:14828954-14829965 REVERSE LENGTH=277
AT5G48070.1 | Symbols: ATXTH20 XTH20 | xyloglucan endotransglucosylase/hydrolase 20 | chr5:19482158-19483231 FORWARD LENGTH=282
AT4G30270.1 | Symbols: MERI5B MERI-5 XTH24 SEN4 | xyloglucan endotransglucosylase/hydrolase 24 | chr4:14819445-14820448 REVERSE LENGTH=269
AT5G57560.1 | Symbols: TCH4 XTH22 | Xyloglucan endotransglucosylase/hydrolase family protein | chr5:23307296-23308235 REVERSE LENGTH=284
AT4G25810.1 | Symbols: XTR6 XTH23 | xyloglucan endotransglycosylase 6 | chr4:13128694-13129715 FORWARD LENGTH=286
AT2G18800.1 | Symbols: ATXTH21 XTH21 | xyloglucan endotransglucosylase/hydrolase 21 | chr2:8144947-8146265 FORWARD LENGTH=305
Peaxi162Scf00315g00820.1 Xyloglucan endotransglucosylase/hydrolase family protein (PhXTH19a)
Peaxi162Scf00324g00088.1 xyloglucan endotransglucosylase/hydrolase 25 (PhXTH19b)
Peaxi162Scf00610g00366.1 Xyloglucan endotransglucosylase/hydrolase family protein (PhXTH19c)
Peaxi162Scf00610g00353.1 xyloglucan endotransglucosylase/hydrolase 25 (PhXTH19d)
Peaxi162Scf00610g00354.1 xyloglucan endotransglucosylase/hydrolase 24 (PhXTH19e)
Peaxi162Scf01867g00005.1 Xyloglucan endotransglucosylase/hydrolase family protein (PhXTH19f)
Peaxi162Scf00610g00361.1 Xyloglucan endotransglucosylase/hydrolase family protein (PhXTH19g)
AT5G57550.1 | Symbols: XTR3 XTH25 | xyloglucan endotransglucosylase/hydrolase 25 | chr5:23305055-23306384 REVERSE LENGTH=284
Peaxi162Scf00610g00349.1 xyloglucan endotransglucosylase/hydrolase 25
AT4G14130.1 | Symbols: XTR7 XTH15 | xyloglucan endotransglucosylase/hydrolase 15 | chr4:8137161-8138196 REVERSE LENGTH=289
AT3G23730.1 | Symbols: XTH16 | xyloglucan endotransglucosylase/hydrolase 16 | chr3:8550222-8551248 FORWARD LENGTH=291
Peaxi162Scf00286g01034.1 xyloglucan endotransglucosylase/hydrolase 15
Peaxi162Scf00111g00013.1 xyloglucan endotransglucosylase/hydrolase 16
Peaxi162Scf00111g00022.1 Xyloglucan endotransglucosylase/hydrolase family protein
Peaxi162Scf00111g00032.1 xyloglucan endotransglucosylase/hydrolase 15
Peaxi162Scf00019g02516.1 xyloglucan endotransglucosylase/hydrolase 24
Peaxi162Scf00807g00217.1 xyloglucan endotransglucosylase/hydrolase 15
Peaxi162Scf00807g00214.1 xyloglucan endotransglucosylase/hydrolase 15
AT5G57540.1 | Symbols: XTH13 AtXTH13 | xyloglucan endotransglucosylase/hydrolase 13 | chr5:23302996-23304049 REVERSE LENGTH=284
Peaxi162Scf00665g00142.1 xyloglucan endotransglucosylase/hydrolase 26
Peaxi162Scf00665g00145.1 xyloglucan endotransglucosylase/hydrolase 26
Peaxi162Scf00539g00612.1 xyloglucan endotransglucosylase/hydrolase 26
Peaxi162Scf00539g00713.1 xyloglucan endotransglucosylase/hydrolase 26
Peaxi162Scf00539g00615.1 xyloglucan endotransglucosylase/hydrolase 26
Peaxi162Scf00037g01026.1 xyloglucan endotransglucosylase/hydrolase 5
Peaxi162Scf00949g00113.1 xyloglucan endotransglucosylase/hydrolase 5
Peaxi162Scf00096g01616.1 xyloglucan endotransglucosylase/hydrolase 6
Peaxi162Scf00096g01615.1 xyloglucan endotransglucosylase/hydrolase 7
Peaxi162Scf00016g03340.1 xyloglucan endotransglucosylase/hydrolase 10
Peaxi162Scf00079g00614.1 xyloglucan endotransglucosylase/hydrolase 8
Peaxi162Scf00007g01511.1 xyloglucan endotransglucosylase/hydrolase 9
Peaxi162Scf00171g00320.1 xyloglucan endotransglucosylase/hydrolase 9

CLF and SWN

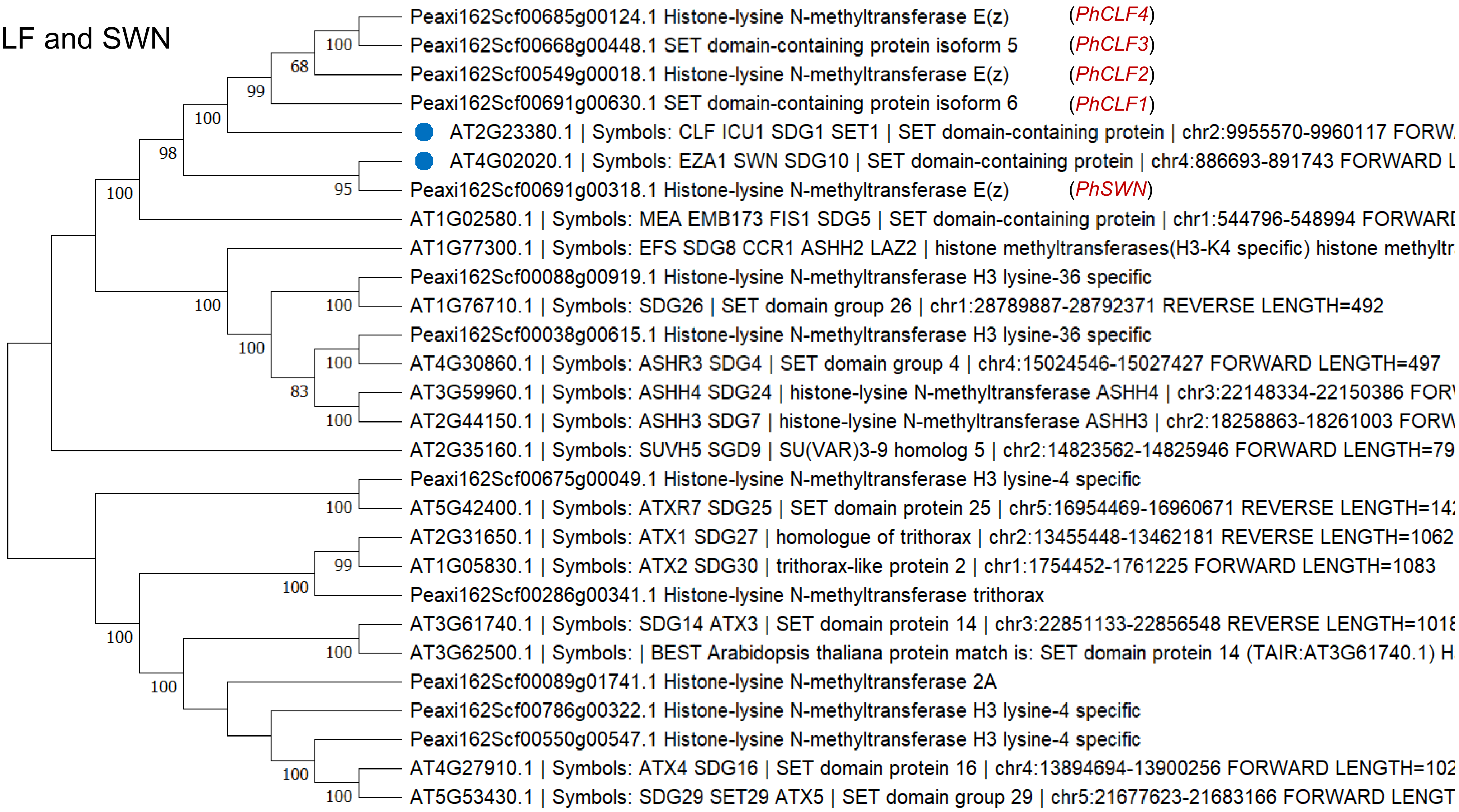
Peaxi162Scf00685g00124.1 Histone-lysine N-methyltransferase E(z) (PhCLF4)
Peaxi162Scf00668g00448.1 SET domain-containing protein isoform 5 (PhCLF3)
Peaxi162Scf00549g00018.1 Histone-lysine N-methyltransferase E(z) (PhCLF2)
Peaxi162Scf00691g00630.1 SET domain-containing protein isoform 6 (PhCLF1)
AT2G23380.1 | Symbols: CLF ICU1 SDG1 SET1 | SET domain-containing protein | chr2:9955570-9960117 FORW
AT4G02020.1 | Symbols: EZA1 SWN SDG10 | SET domain-containing protein | chr4:886693-891743 FORWARD L
Peaxi162Scf00691g00318.1 Histone-lysine N-methyltransferase E(z) (PhSWN)
AT1G02580.1 | Symbols: MEA EMB173 FIS1 SDG5 | SET domain-containing protein | chr1:544796-548994 FORWARI
AT1G77300.1 | Symbols: EFS SDG8 CCR1 ASHH2 LAZ2 | histone methyltransferases(H3-K4 specific) histone methyltr
Peaxi162Scf00088g00919.1 Histone-lysine N-methyltransferase H3 lysine-36 specific
AT1G76710.1 | Symbols: SDG26 | SET domain group 26 | chr1:28789887-28792371 REVERSE LENGTH=492
Peaxi162Scf00038g00615.1 Histone-lysine N-methyltransferase H3 lysine-36 specific
AT4G30860.1 | Symbols: ASHR3 SDG4 | SET domain group 4 | chr4:15024546-15027427 FORWARD LENGTH=497
AT3G59960.1 | Symbols: ASHH4 SDG24 | histone-lysine N-methyltransferase ASHH4 | chr3:22148334-22150386 FOR
AT2G44150.1 | Symbols: ASHH3 SDG7 | histone-lysine N-methyltransferase ASHH3 | chr2:18258863-18261003 FORW
AT2G35160.1 | Symbols: SUVH5 SGD9 | SU(VAR)3-9 homolog 5 | chr2:14823562-14825946 FORWARD LENGTH=79
Peaxi162Scf00675g00049.1 Histone-lysine N-methyltransferase H3 lysine-4 specific
AT5G42400.1 | Symbols: ATXR7 SDG25 | SET domain protein 25 | chr5:16954469-16960671 REVERSE LENGTH=14
AT2G31650.1 | Symbols: ATX1 SDG27 | homologue of trithorax | chr2:13455448-13462181 REVERSE LENGTH=1062
AT1G05830.1 | Symbols: ATX2 SDG30 | trithorax-like protein 2 | chr1:1754452-1761225 FORWARD LENGTH=1083
Peaxi162Scf00286g00341.1 Histone-lysine N-methyltransferase trithorax
AT3G61740.1 | Symbols: SDG14 ATX3 | SET domain protein 14 | chr3:22851133-22856548 REVERSE LENGTH=101
AT3G62500.1 | Symbols: | BEST Arabidopsis thaliana protein match is: SET domain protein 14 (TAIR:AT3G61740.1) H
Peaxi162Scf00089g01741.1 Histone-lysine N-methyltransferase 2A
Peaxi162Scf00786g00322.1 Histone-lysine N-methyltransferase H3 lysine-4 specific
Peaxi162Scf00550g00547.1 Histone-lysine N-methyltransferase H3 lysine-4 specific
AT4G27910.1 | Symbols: ATX4 SDG16 | SET domain protein 16 | chr4:13894694-13900256 FORWARD LENGTH=102
AT5G53430.1 | Symbols: SDG29 SET29 ATX5 | SET domain group 29 | chr5:21677623-21683166 FORWARD LENGT
100
68
99
100
98
95
100
100
100
100
100
83
100
100
99
100
100
100
100
100
100

FIE

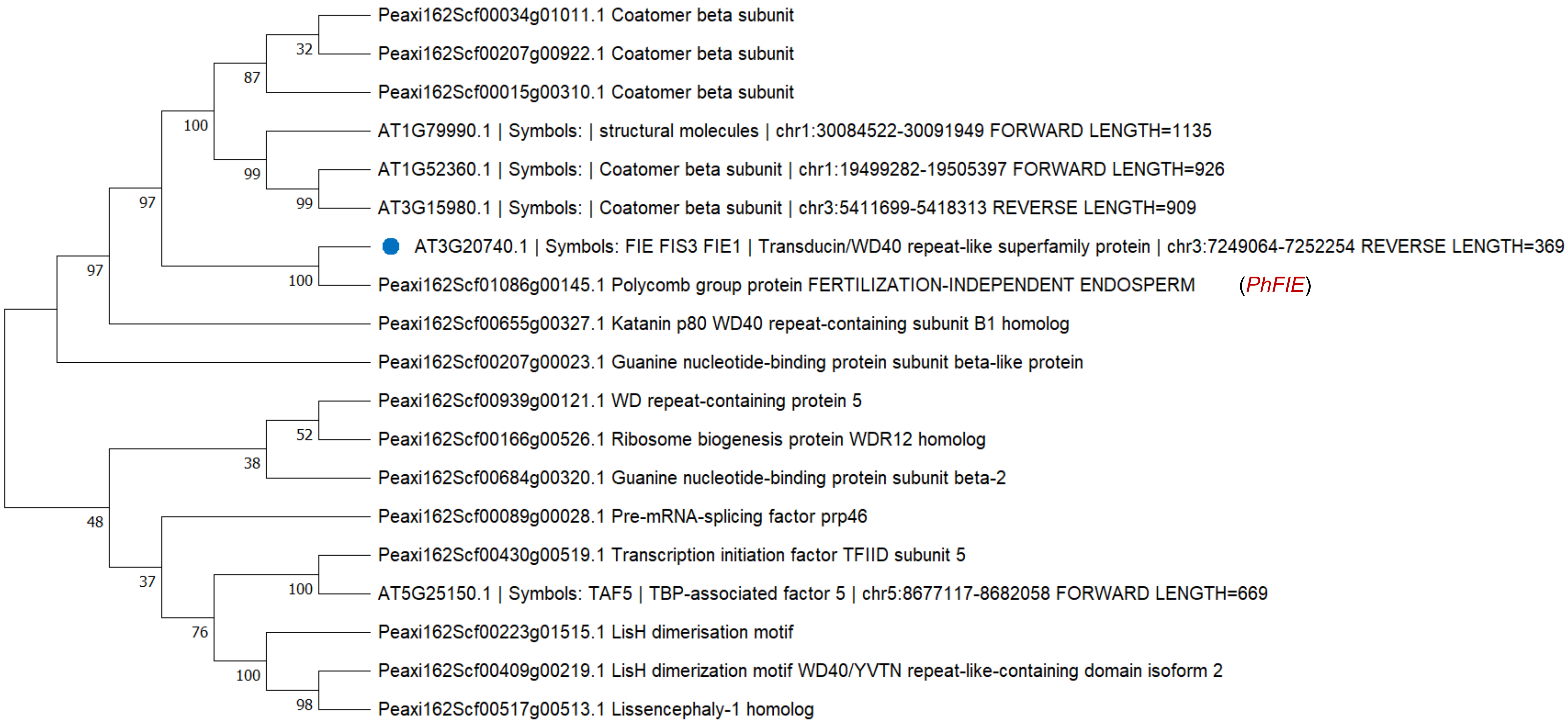

Peaxi162Scf00034g01011.1 Coatomer beta subunit
Peaxi162Scf00207g00922.1 Coatomer beta subunit
Peaxi162Scf00015g00310.1 Coatomer beta subunit
AT1G79990.1 | Symbols: | structural molecules | chr1:30084522-30091949 FORWARD LENGTH=1135
AT1G52360.1 | Symbols: | Coatomer beta subunit | chr1:19499282-19505397 FORWARD LENGTH=926
AT3G15980.1 | Symbols: | Coatomer beta subunit | chr3:5411699-5418313 REVERSE LENGTH=909
AT3G20740.1 | Symbols: FIE FIS3 FIE1 | Transducin/WD40 repeat-like superfamily protein | chr3:7249064-7252254 REVERSE LENGTH=369
Peaxi162Scf01086g00145.1 Polycomb group protein FERTILIZATION-INDEPENDENT ENDOSPERM
(PhFIE)
Peaxi162Scf00655g00327.1 Katanin p80 WD40 repeat-containing subunit B1 homolog
Peaxi162Scf00207g00023.1 Guanine nucleotide-binding protein subunit beta-like protein
Peaxi162Scf00939g00121.1 WD repeat-containing protein 5
Peaxi162Scf00166g00526.1 Ribosome biogenesis protein WDR12 homolog
Peaxi162Scf00684g00320.1 Guanine nucleotide-binding protein subunit beta-2
Peaxi162Scf00089g00028.1 Pre-mRNA-splicing factor prp46
Peaxi162Scf00430g00519.1 Transcription initiation factor TFIID subunit 5
AT5G25150.1 | Symbols: TAF5 | TBP-associated factor 5 | chr5:8677117-8682058 FORWARD LENGTH=669
Peaxi162Scf00223g01515.1 LisH dimerisation motif
Peaxi162Scf00409g00219.1 LisH dimerization motif WD40/YVTN repeat-like-containing domain isoform 2
Peaxi162Scf00517g00513.1 Lissencephaly-1 homolog
32
87
100
99
99
97
97
100
52
38
48
37
100
76
100
98

ARABIDOPSIS TRITHORAX-RELATED 2 (*ATXR2*)

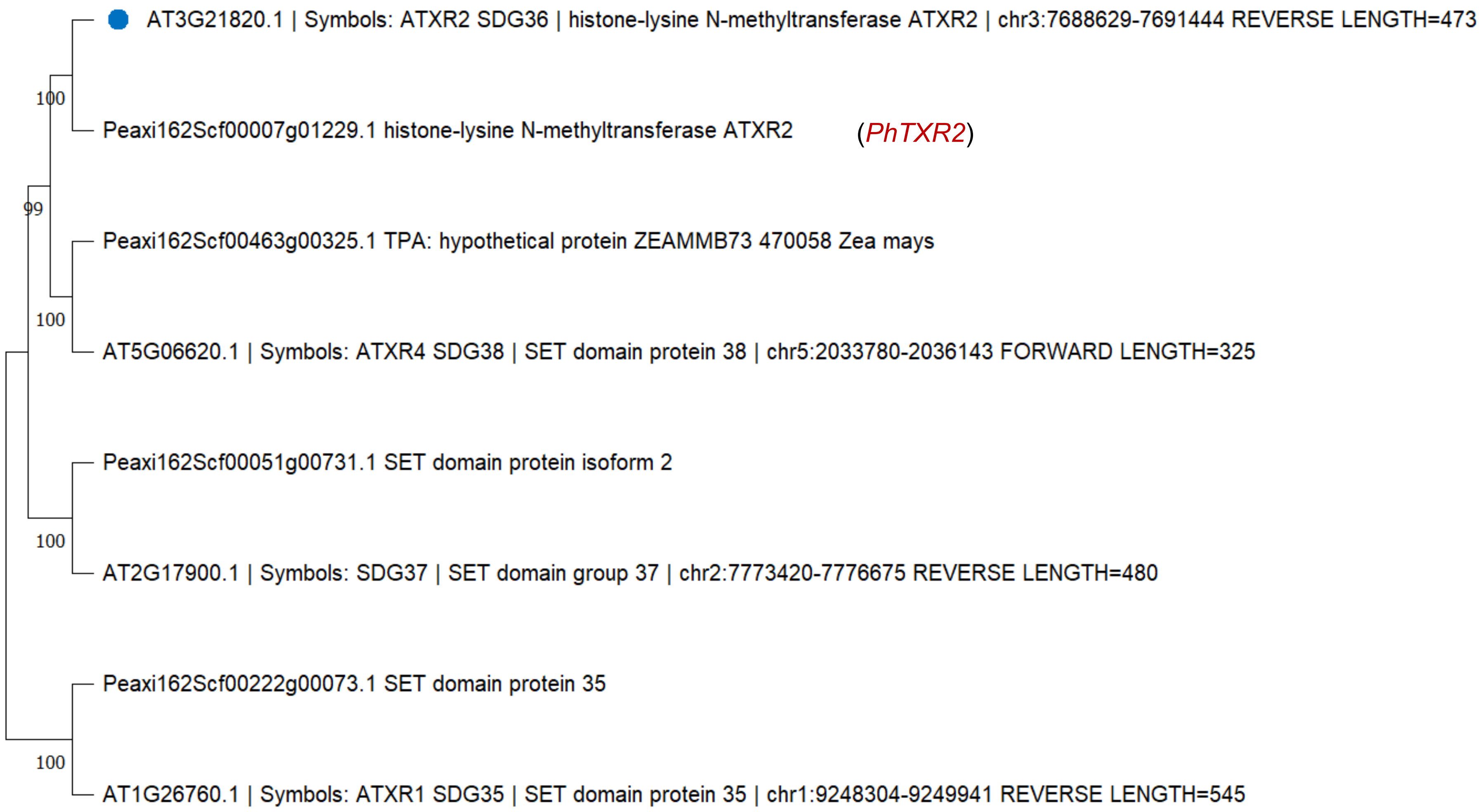

JMJ30

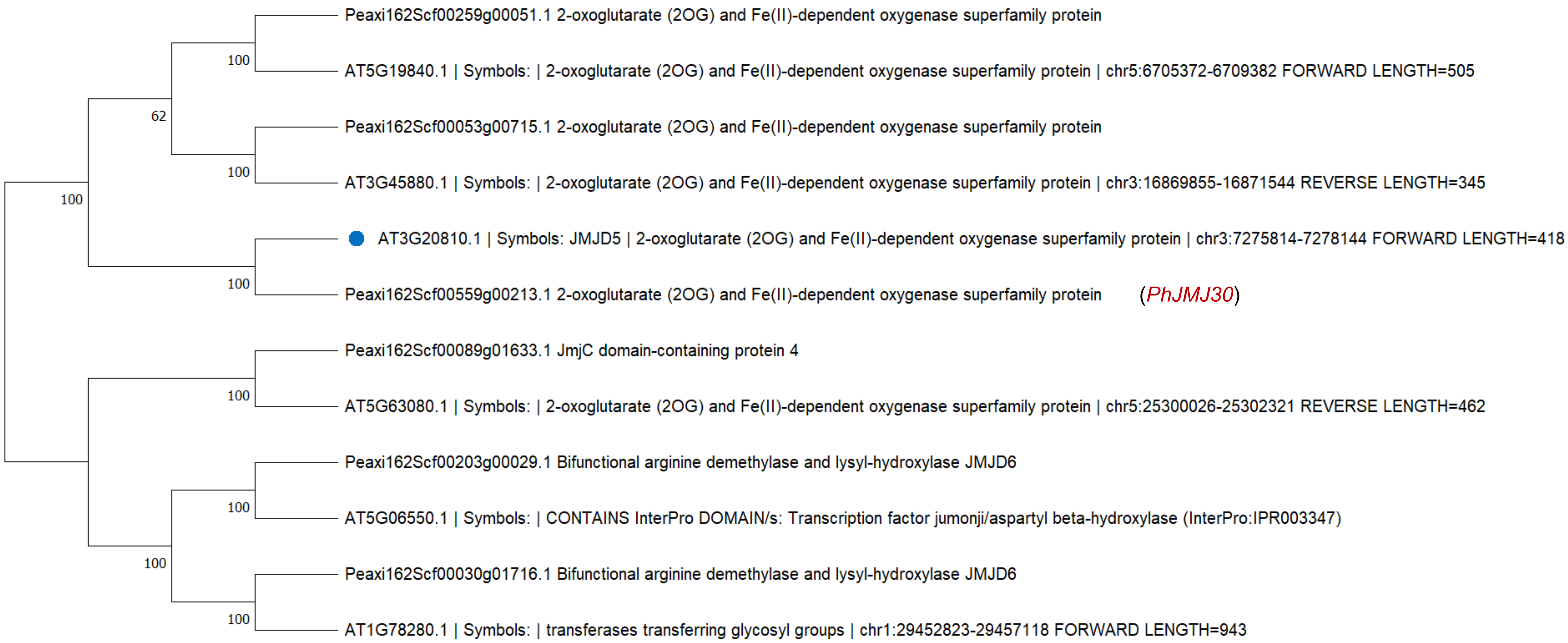
Peaxi162Scf00259g00051.1 2-oxoglutarate (2OG) and Fe(II)-dependent oxygenase superfamily protein
100
AT5G19840.1 | Symbols: | 2-oxoglutarate (2OG) and Fe(II)-dependent oxygenase superfamily protein | chr5:6705372-6709382 FORWARD LENGTH=505
62
Peaxi162Scf00053g00715.1 2-oxoglutarate (2OG) and Fe(II)-dependent oxygenase superfamily protein
100
AT3G45880.1 | Symbols: | 2-oxoglutarate (2OG) and Fe(II)-dependent oxygenase superfamily protein | chr3:16869855-16871544 REVERSE LENGTH=345
100
AT3G20810.1 | Symbols: JMJD5 | 2-oxoglutarate (2OG) and Fe(II)-dependent oxygenase superfamily protein | chr3:7275814-7278144 FORWARD LENGTH=418
100
Peaxi162Scf00559g00213.1 2-oxoglutarate (2OG) and Fe(II)-dependent oxygenase superfamily protein (*PhJMJ30*)
Peaxi162Scf00089g01633.1 JmjC domain-containing protein 4
100
AT5G63080.1 | Symbols: | 2-oxoglutarate (2OG) and Fe(II)-dependent oxygenase superfamily protein | chr5:25300026-25302321 REVERSE LENGTH=462
Peaxi162Scf00203g00029.1 Bifunctional arginine demethylase and lysyl-hydroxylase JMJD6
100
AT5G06550.1 | Symbols: | CONTAINS InterPro DOMAIN/s: Transcription factor jumonji/aspartyl beta-hydroxylase (InterPro:IPR003347)
100
Peaxi162Scf00030g01716.1 Bifunctional arginine demethylase and lysyl-hydroxylase JMJD6
100
AT1G78280.1 | Symbols: | transferases transferring glycosyl groups | chr1:29452823-29457118 FORWARD LENGTH=943

HAG1/GCN5

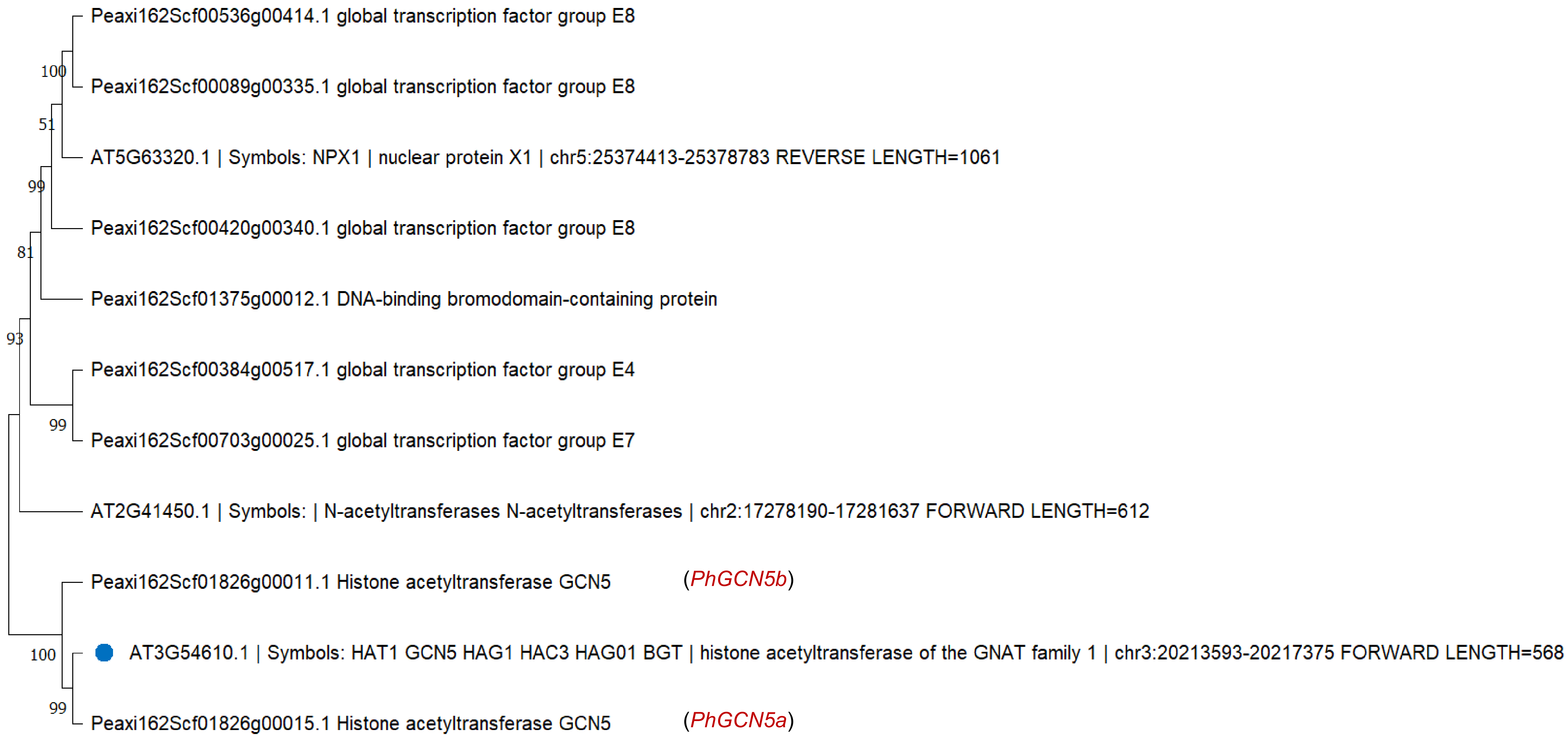

Peaxi162Scf00536g00414.1 global transcription factor group E8
100
Peaxi162Scf00089g00335.1 global transcription factor group E8
51
AT5G63320.1 | Symbols: NPX1 | nuclear protein X1 | chr5:25374413-25378783 REVERSE LENGTH=1061
99
Peaxi162Scf00420g00340.1 global transcription factor group E8
81
Peaxi162Scf01375g00012.1 DNA-binding bromodomain-containing protein
93
Peaxi162Scf00384g00517.1 global transcription factor group E4
99
Peaxi162Scf00703g00025.1 global transcription factor group E7
AT2G41450.1 | Symbols: | N-acetyltransferases N-acetyltransferases | chr2:17278190-17281637 FORWARD LENGTH=612
Peaxi162Scf01826g00011.1 Histone acetyltransferase GCN5
(PhGCN5b)
100
AT3G54610.1 | Symbols: HAT1 GCN5 HAG1 HAC3 HAG01 BGT | histone acetyltransferase of the GNAT family 1 | chr3:20213593-20217375 FORWARD LENGTH=568
99
Peaxi162Scf01826g00015.1 Histone acetyltransferase GCN5
(PhGCN5a)

MET1

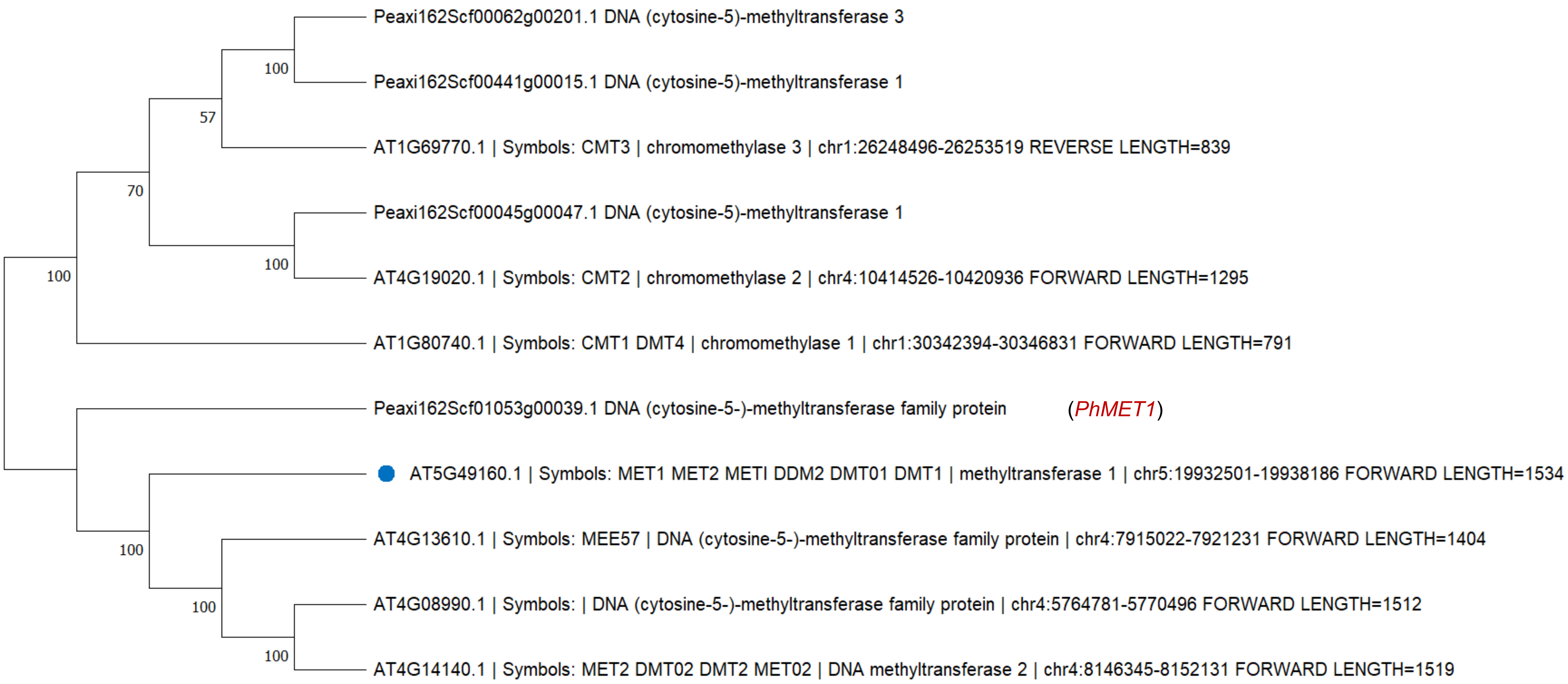

Peaxi162Scf00062g00201.1 DNA (cytosine-5)-methyltransferase 3
100
Peaxi162Scf00441g00015.1 DNA (cytosine-5)-methyltransferase 1
57
AT1G69770.1 | Symbols: CMT3 | chromomethylase 3 | chr1:26248496-26253519 REVERSE LENGTH=839
70
Peaxi162Scf00045g00047.1 DNA (cytosine-5)-methyltransferase 1
100
100
AT4G19020.1 | Symbols: CMT2 | chromomethylase 2 | chr4:10414526-10420936 FORWARD LENGTH=1295
AT1G80740.1 | Symbols: CMT1 DMT4 | chromomethylase 1 | chr1:30342394-30346831 FORWARD LENGTH=791
Peaxi162Scf01053g00039.1 DNA (cytosine-5-)-methyltransferase family protein
(PhMET1)
AT5G49160.1 | Symbols: MET1 MET2 METI DDM2 DMT01 DMT1 | methyltransferase 1 | chr5:19932501-19938186 FORWARD LENGTH=1534
AT4G13610.1 | Symbols: MEE57 | DNA (cytosine-5-)-methyltransferase family protein | chr4:7915022-7921231 FORWARD LENGTH=1404
100
100
AT4G08990.1 | Symbols: | DNA (cytosine-5-)-methyltransferase family protein | chr4:5764781-5770496 FORWARD LENGTH=1512
100
AT4G14140.1 | Symbols: MET2 DMT02 DMT2 MET02 | DNA methyltransferase 2 | chr4:8146345-8152131 FORWARD LENGTH=1519

Phylogenetic trees of cytokinin metabolism and signaling genes

Isopentenyl Transferase (*IPT*) Genes

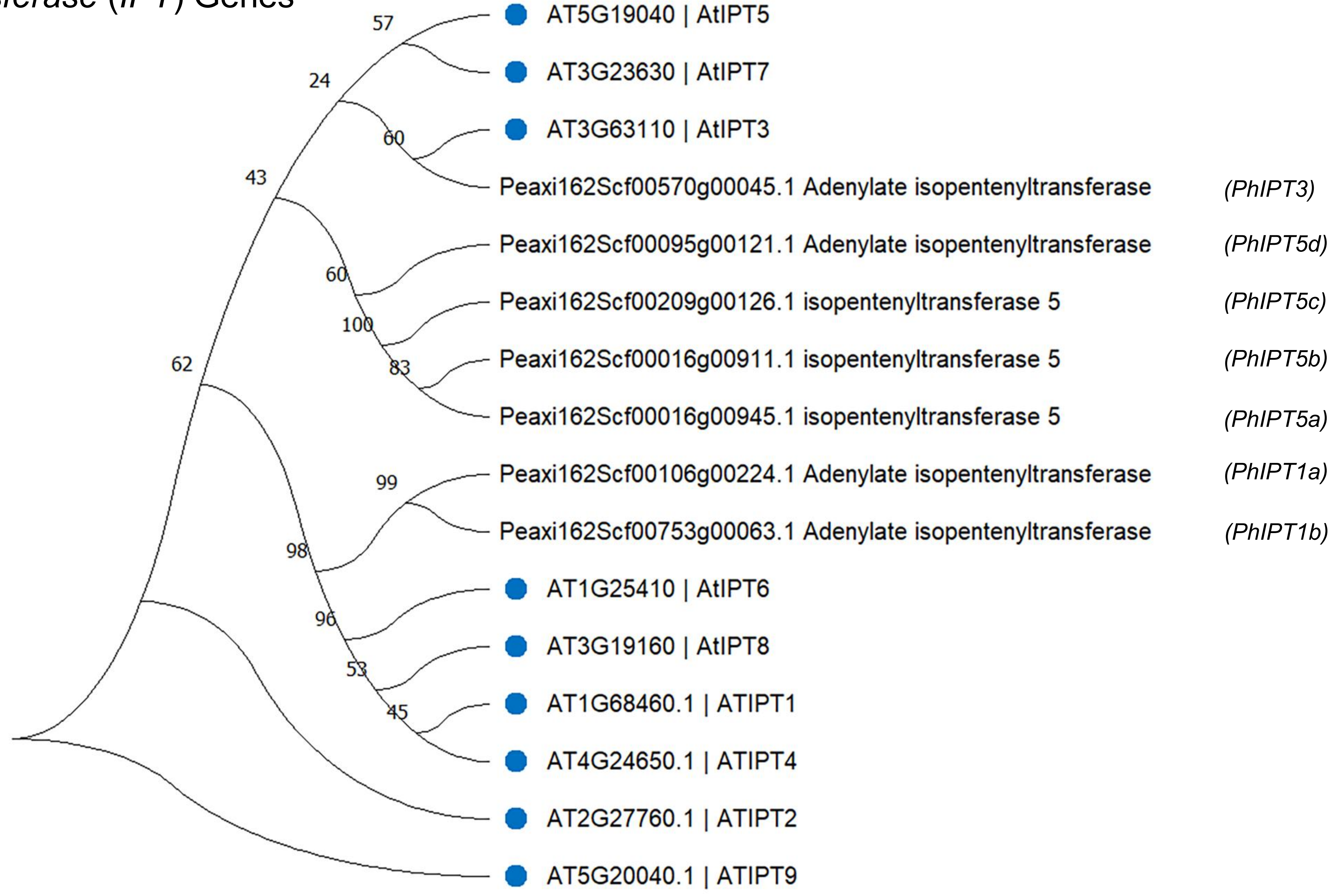

LONELY GUY (LOG) Genes

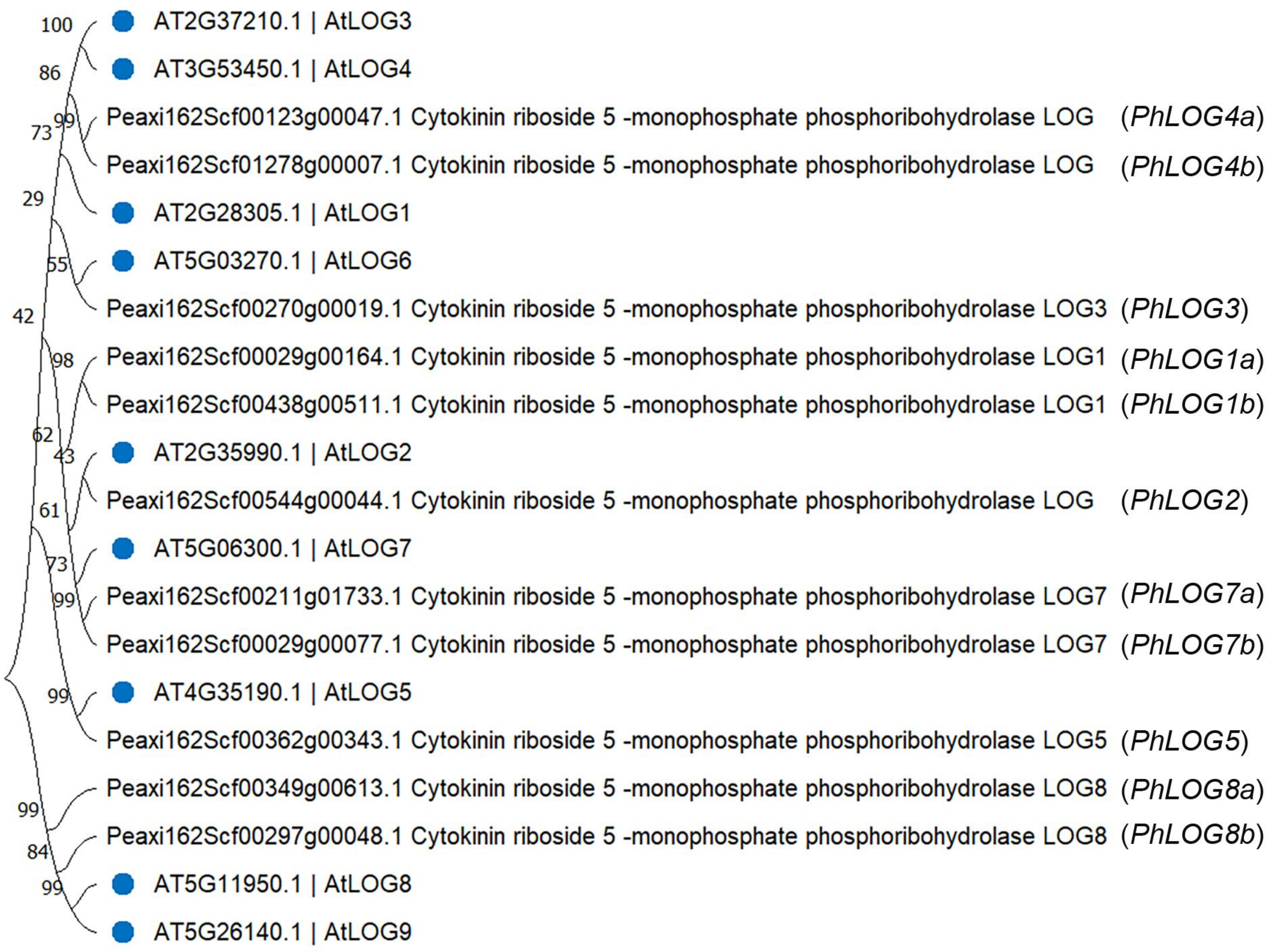

Cytokinin Oxidase (*CKX*) Genes

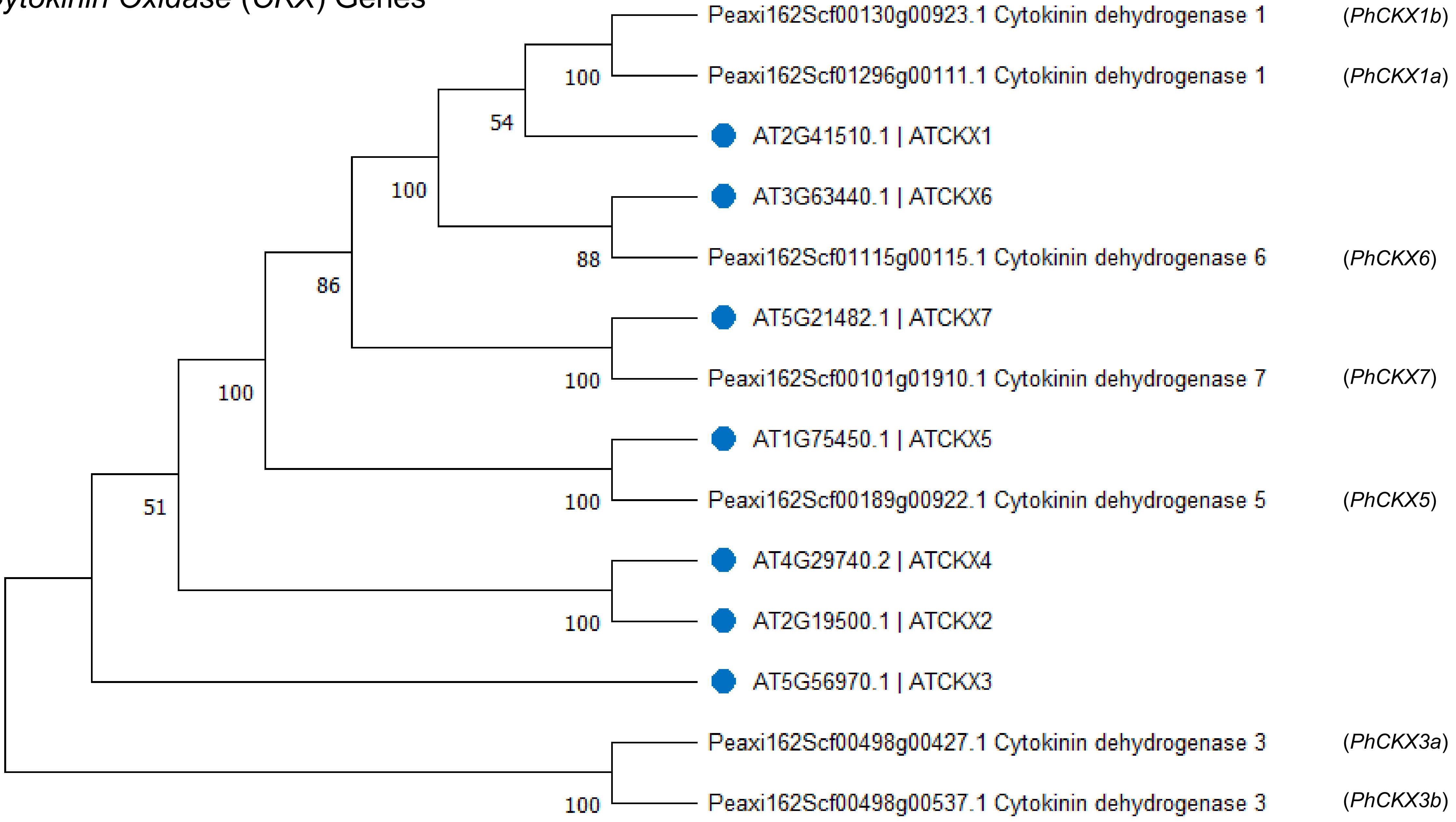

Histidine Kinase (*HK*) Genes

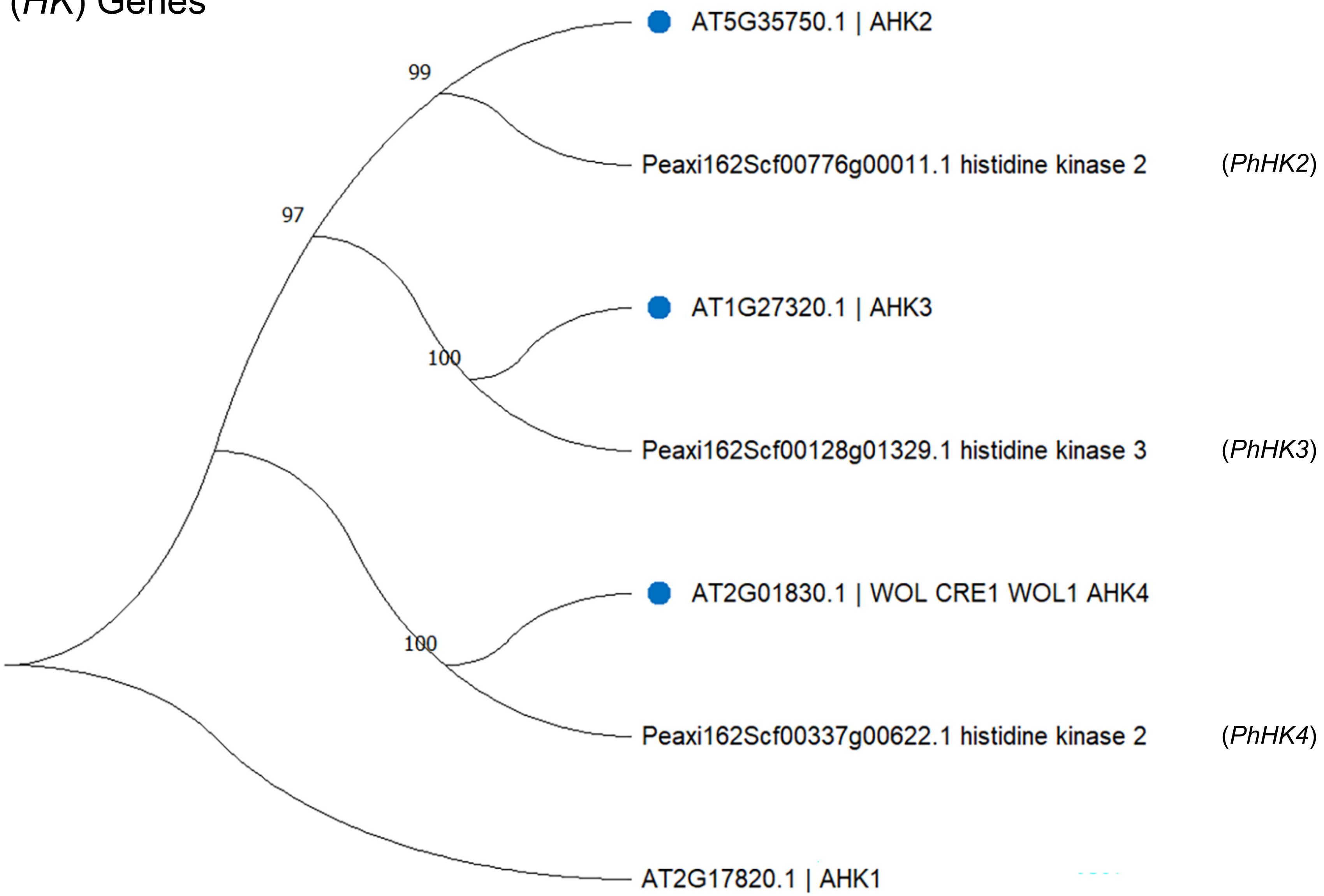

Histidine Phosphotransfer Protein (HP) genes

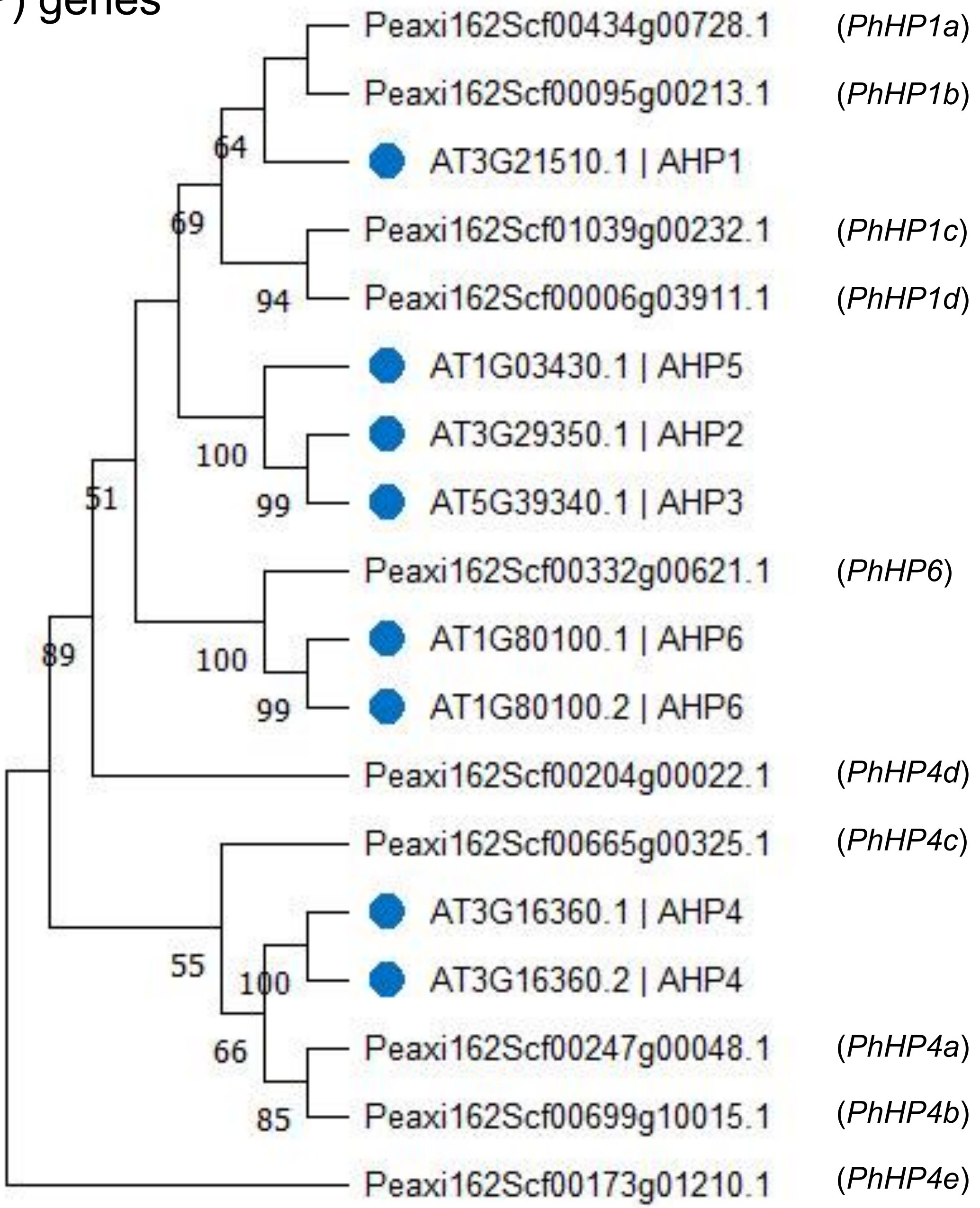

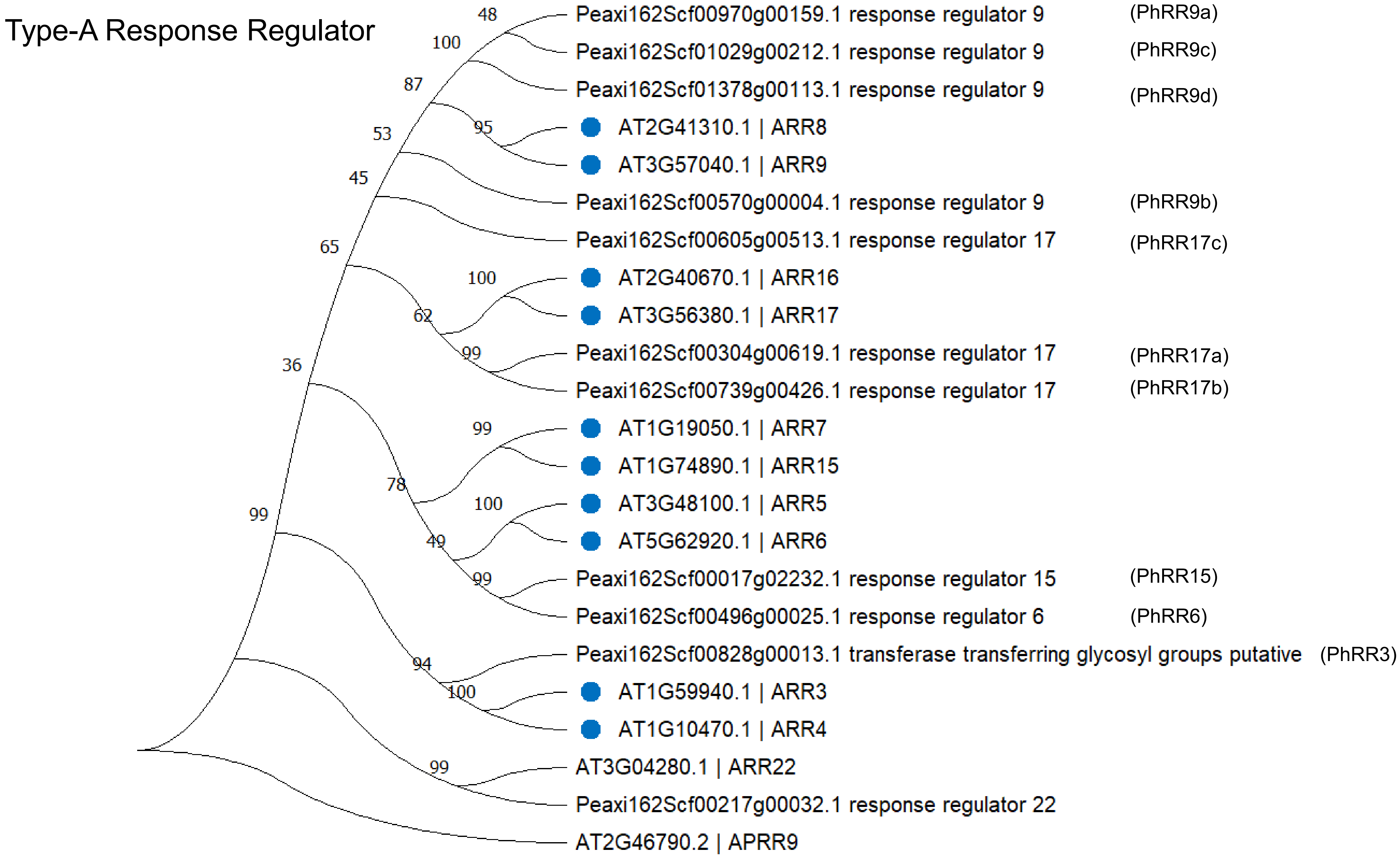
Type-A Response Regulator
Peaxi162Scf00970g00159.1 response regulator 9 (PhRR9a)
Peaxi162Scf01029g00212.1 response regulator 9 (PhRR9c)
Peaxi162Scf01378g00113.1 response regulator 9 (PhRR9d)
AT2G41310.1 | ARR8
AT3G57040.1 | ARR9
Peaxi162Scf00570g00004.1 response regulator 9 (PhRR9b)
Peaxi162Scf00605g00513.1 response regulator 17 (PhRR17c)
AT2G40670.1 | ARR16
AT3G56380.1 | ARR17
Peaxi162Scf00304g00619.1 response regulator 17 (PhRR17a)
Peaxi162Scf00739g00426.1 response regulator 17 (PhRR17b)
AT1G19050.1 | ARR7
AT1G74890.1 | ARR15
AT3G48100.1 | ARR5
AT5G62920.1 | ARR6
Peaxi162Scf00017g02232.1 response regulator 15 (PhRR15)
Peaxi162Scf00496g00025.1 response regulator 6 (PhRR6)
Peaxi162Scf00828g00013.1 transferase transferring glycosyl groups putative (PhRR3)
AT1G59940.1 | ARR3
AT1G10470.1 | ARR4
AT3G04280.1 | ARR22
Peaxi162Scf00217g00032.1 response regulator 22
AT2G46790.2 | APRR9
48
100
87
95
53
45
65
100
62
99
36
99
78
100
49
99
99
94
100
99

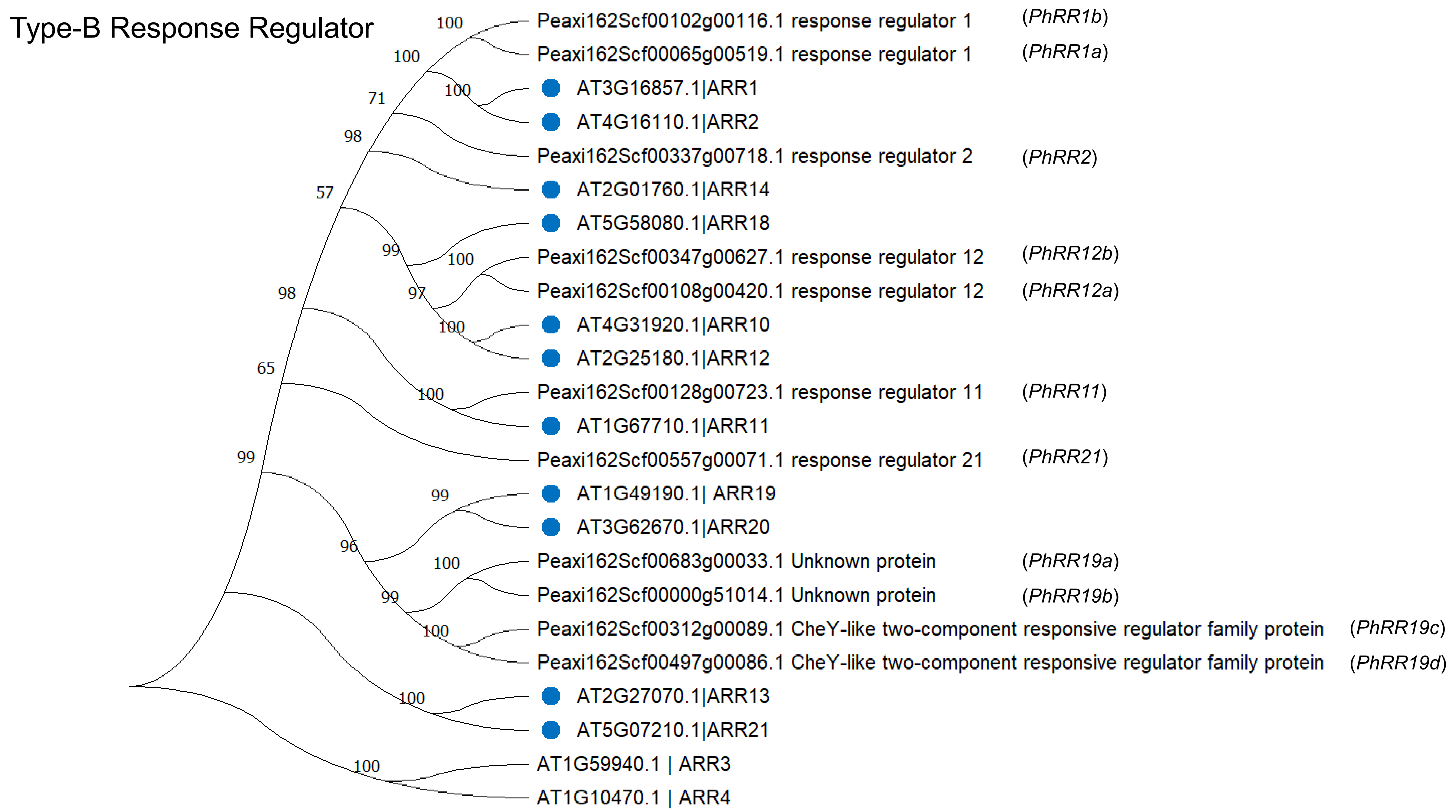

Type-B Response Regulator
Peaxi162Scf00102g00116.1 response regulator 1 (PhRR1b)
Peaxi162Scf00065g00519.1 response regulator 1 (PhRR1a)
AT3G16857.1|ARR1
AT4G16110.1|ARR2
Peaxi162Scf00337g00718.1 response regulator 2 (PhRR2)
AT2G01760.1|ARR14
AT5G58080.1|ARR18
Peaxi162Scf00347g00627.1 response regulator 12 (PhRR12b)
Peaxi162Scf00108g00420.1 response regulator 12 (PhRR12a)
AT4G31920.1|ARR10
AT2G25180.1|ARR12
Peaxi162Scf00128g00723.1 response regulator 11 (PhRR11)
AT1G67710.1|ARR11
Peaxi162Scf00557g00071.1 response regulator 21 (PhRR21)
AT1G49190.1| ARR19
AT3G62670.1|ARR20
Peaxi162Scf00683g00033.1 Unknown protein (PhRR19a)
Peaxi162Scf00000g51014.1 Unknown protein (PhRR19b)
Peaxi162Scf00312g00089.1 CheY-like two-component responsive regulator family protein (PhRR19c)
Peaxi162Scf00497g00086.1 CheY-like two-component responsive regulator family protein (PhRR19d)
AT2G27070.1|ARR13
AT5G07210.1|ARR21
AT1G59940.1 | ARR3
AT1G10470.1 | ARR4
100
100
100
71
98
57
99
100
97
100
98
100
65
99
99
96
100
99
100
100
100

Phylogenetic trees of auxin metabolism and signaling genes

Tryptophan Aminotransferase Genes

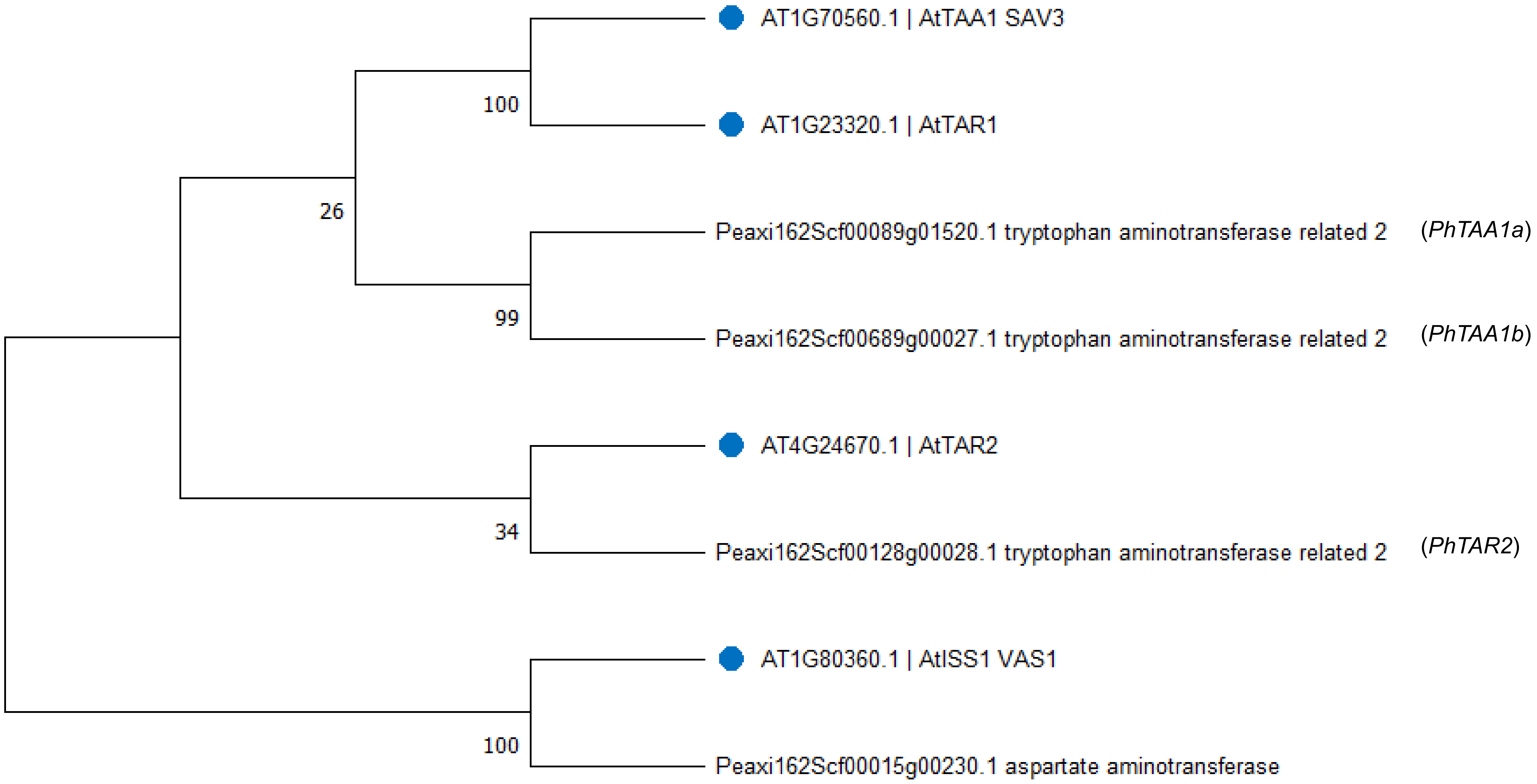

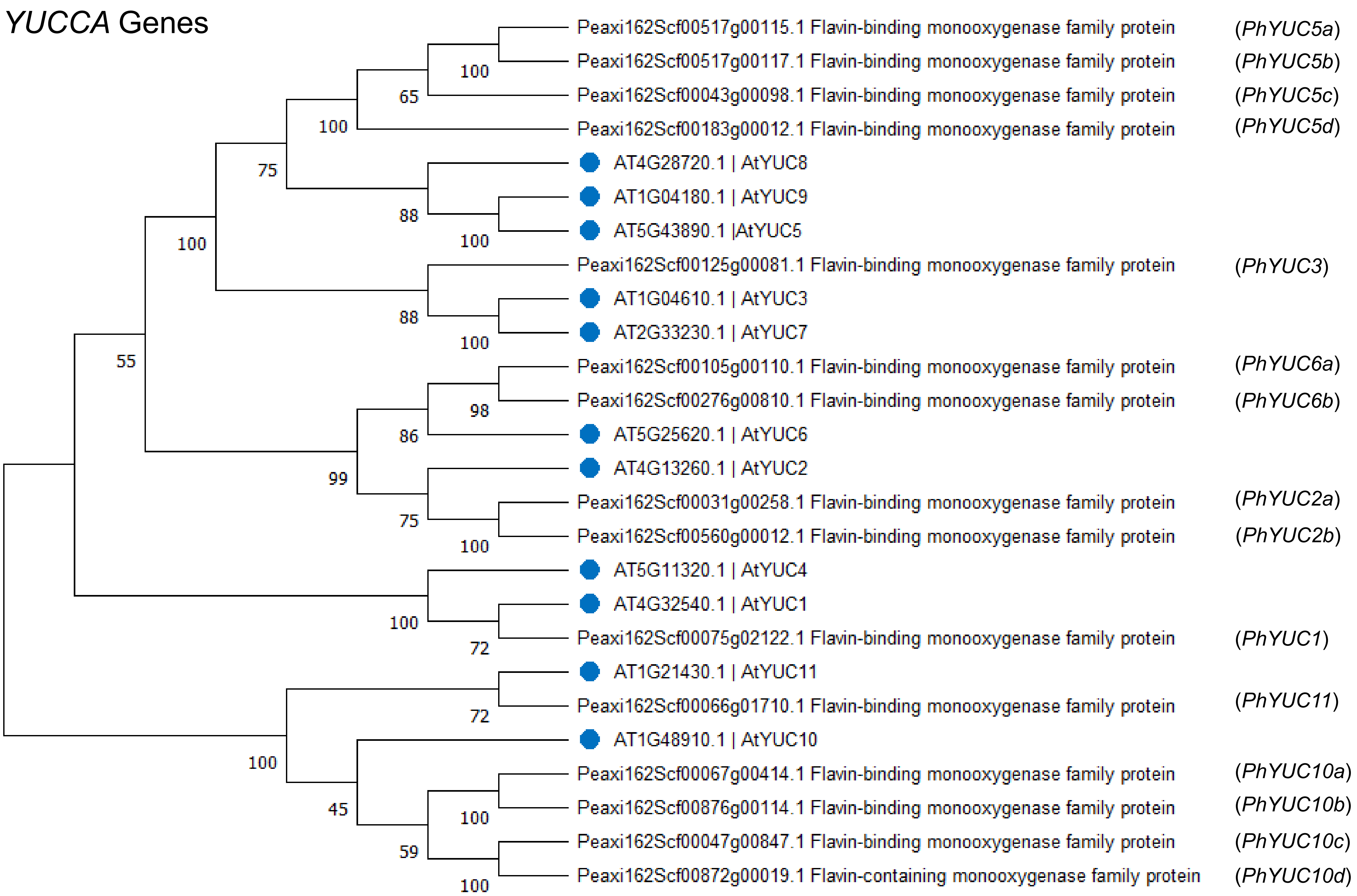
YUCCA Genes
Peaxi162Scf00517g00115.1 Flavin-binding monooxygenase family protein (PhYUC5a)
Peaxi162Scf00517g00117.1 Flavin-binding monooxygenase family protein (PhYUC5b)
Peaxi162Scf00043g00098.1 Flavin-binding monooxygenase family protein (PhYUC5c)
Peaxi162Scf00183g00012.1 Flavin-binding monooxygenase family protein (PhYUC5d)
AT4G28720.1 | AtYUC8
AT1G04180.1 | AtYUC9
AT5G43890.1 |AtYUC5
Peaxi162Scf00125g00081.1 Flavin-binding monooxygenase family protein (PhYUC3)
AT1G04610.1 | AtYUC3
AT2G33230.1 | AtYUC7
Peaxi162Scf00105g00110.1 Flavin-binding monooxygenase family protein (PhYUC6a)
Peaxi162Scf00276g00810.1 Flavin-binding monooxygenase family protein (PhYUC6b)
AT5G25620.1 | AtYUC6
AT4G13260.1 | AtYUC2
Peaxi162Scf00031g00258.1 Flavin-binding monooxygenase family protein (PhYUC2a)
Peaxi162Scf00560g00012.1 Flavin-binding monooxygenase family protein (PhYUC2b)
AT5G11320.1 | AtYUC4
AT4G32540.1 | AtYUC1
Peaxi162Scf00075g02122.1 Flavin-binding monooxygenase family protein (PhYUC1)
AT1G21430.1 | AtYUC11
Peaxi162Scf00066g01710.1 Flavin-binding monooxygenase family protein (PhYUC11)
AT1G48910.1 | AtYUC10
Peaxi162Scf00067g00414.1 Flavin-binding monooxygenase family protein (PhYUC10a)
Peaxi162Scf00876g00114.1 Flavin-binding monooxygenase family protein (PhYUC10b)
Peaxi162Scf00047g00847.1 Flavin-binding monooxygenase family protein (PhYUC10c)
Peaxi162Scf00872g00019.1 Flavin-containing monooxygenase family protein (PhYUC10d)
100
65
100
75
88
100
100
88
100
55
98
86
99
75
100
100
72
72
100
45
100
59
100

DAO (*2-oxoglutarate-dependent dioxygenase*) Genes

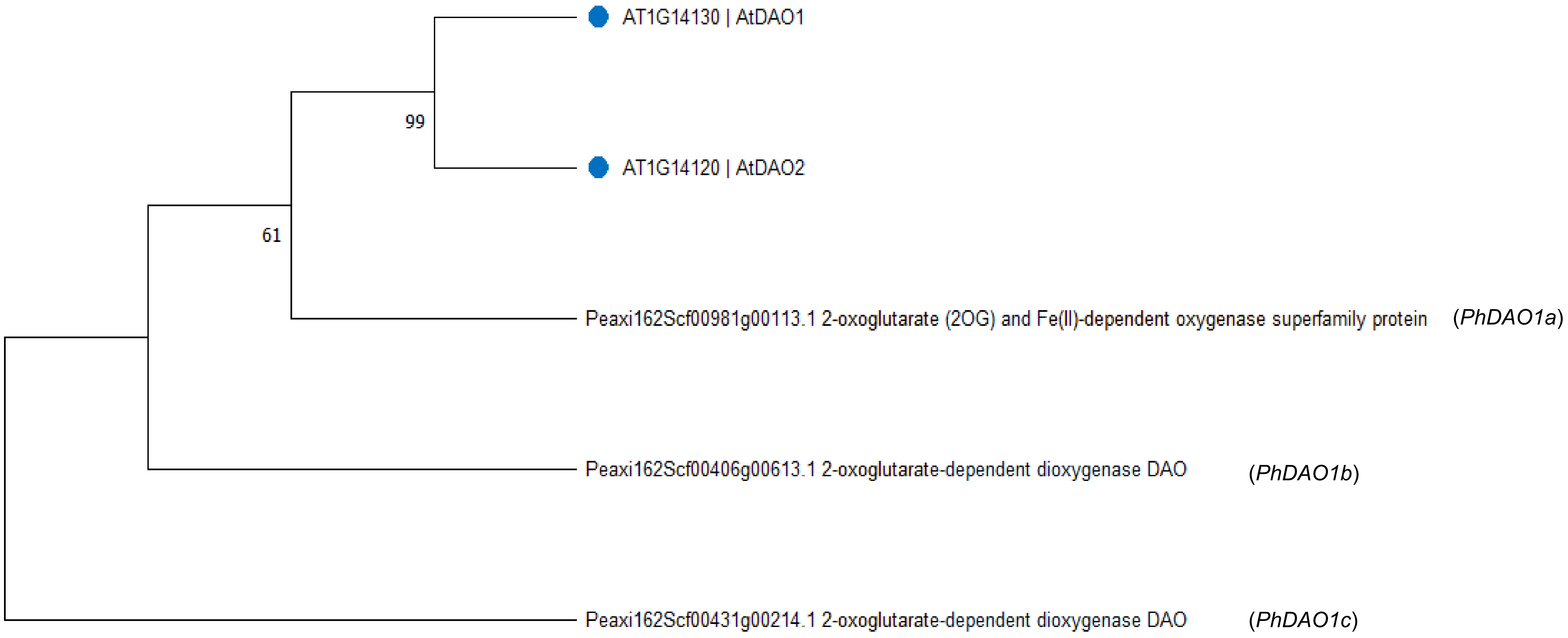

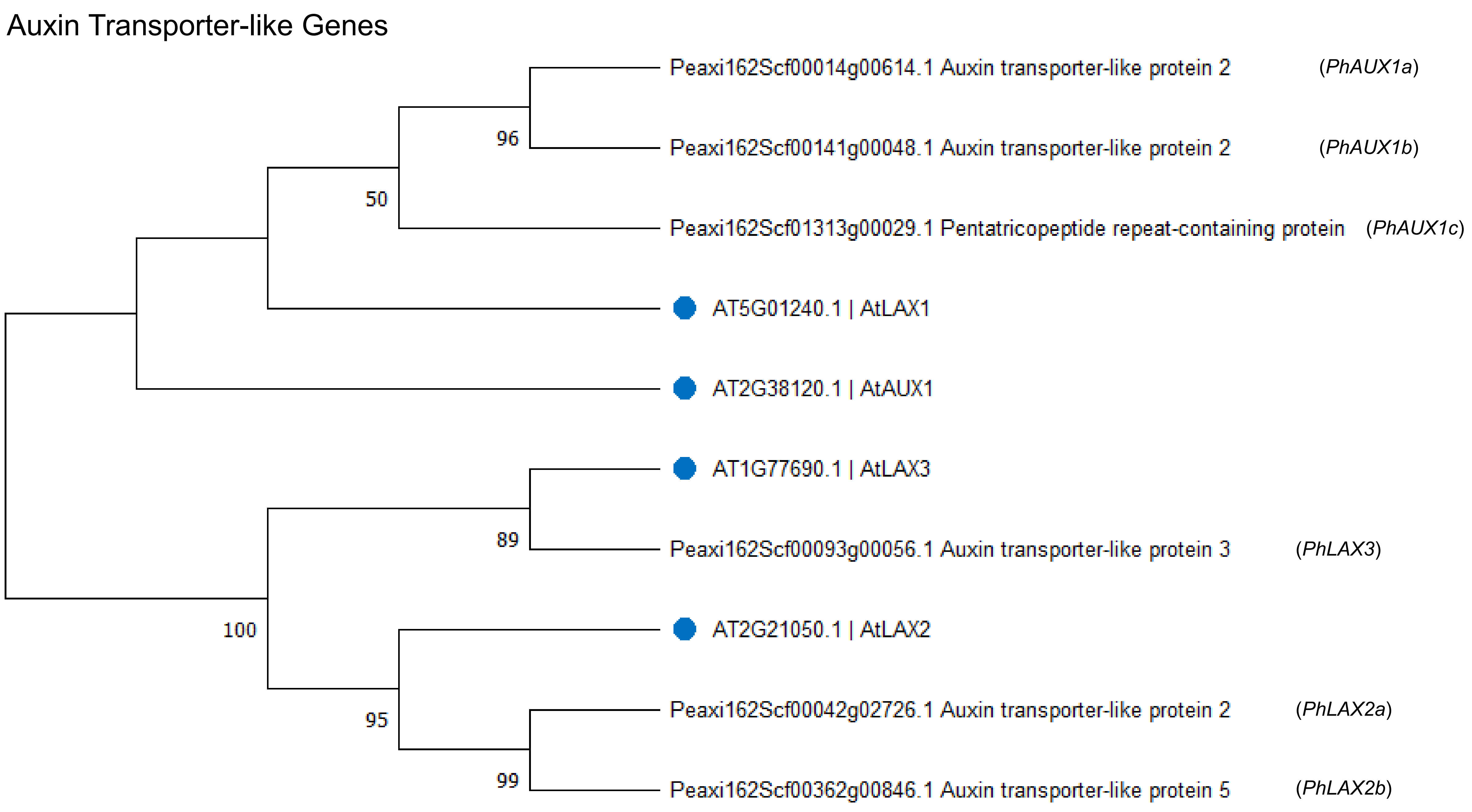

Auxin Transporter-like Genes
Peaxi162Scf00014g00614.1 Auxin transporter-like protein 2
(PhAUX1a)
96
Peaxi162Scf00141g00048.1 Auxin transporter-like protein 2
(PhAUX1b)
50
Peaxi162Scf01313g00029.1 Pentatricopeptide repeat-containing protein
(PhAUX1c)
AT5G01240.1 | AtLAX1
AT2G38120.1 | AtAUX1
AT1G77690.1 | AtLAX3
89
Peaxi162Scf00093g00056.1 Auxin transporter-like protein 3
(PhLAX3)
100
AT2G21050.1 | AtLAX2
95
Peaxi162Scf00042g02726.1 Auxin transporter-like protein 2
(PhLAX2a)
99
Peaxi162Scf00362g00846.1 Auxin transporter-like protein 5
(PhLAX2b)

Auxin Efflux Carrier Family Genes

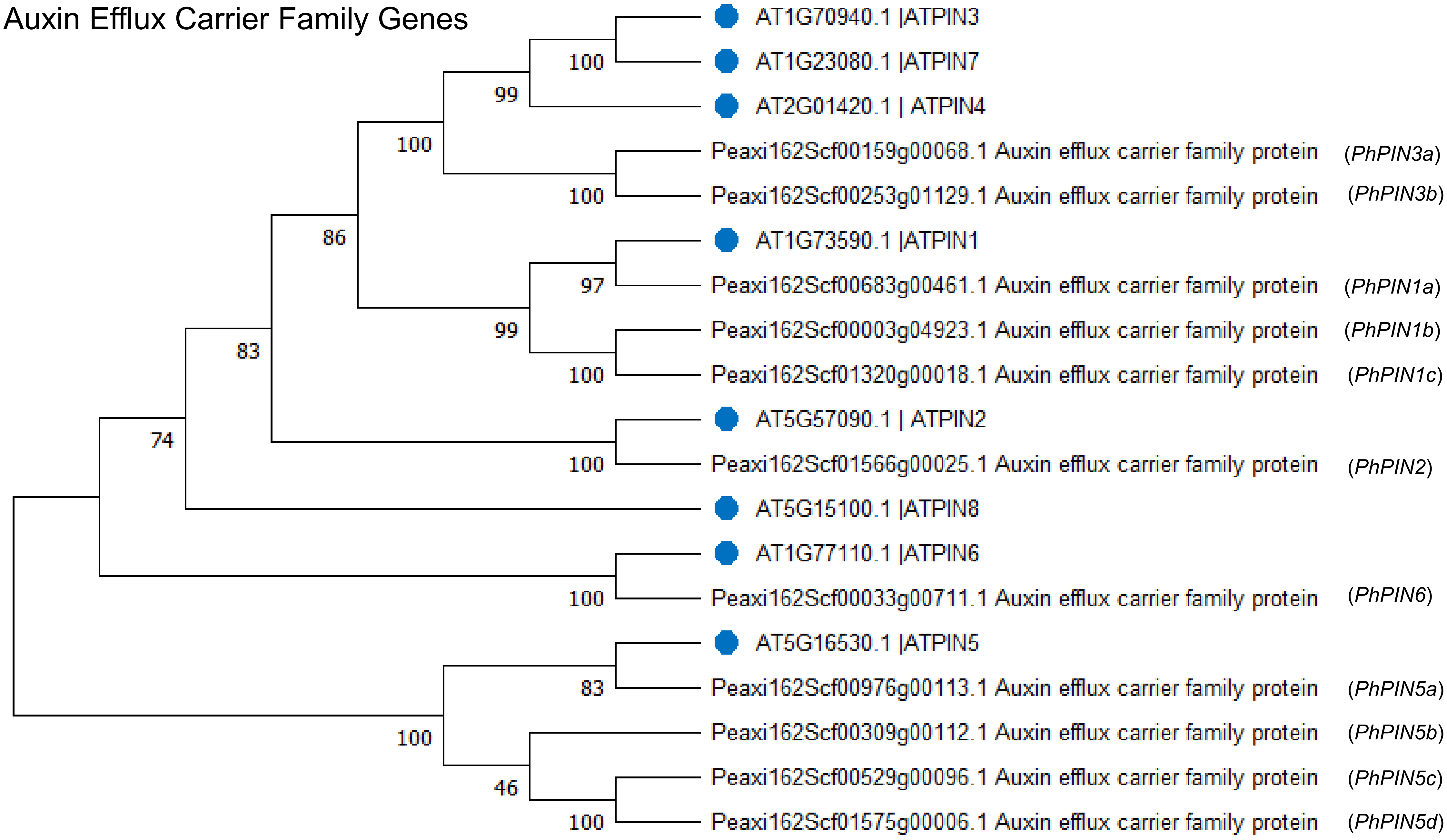

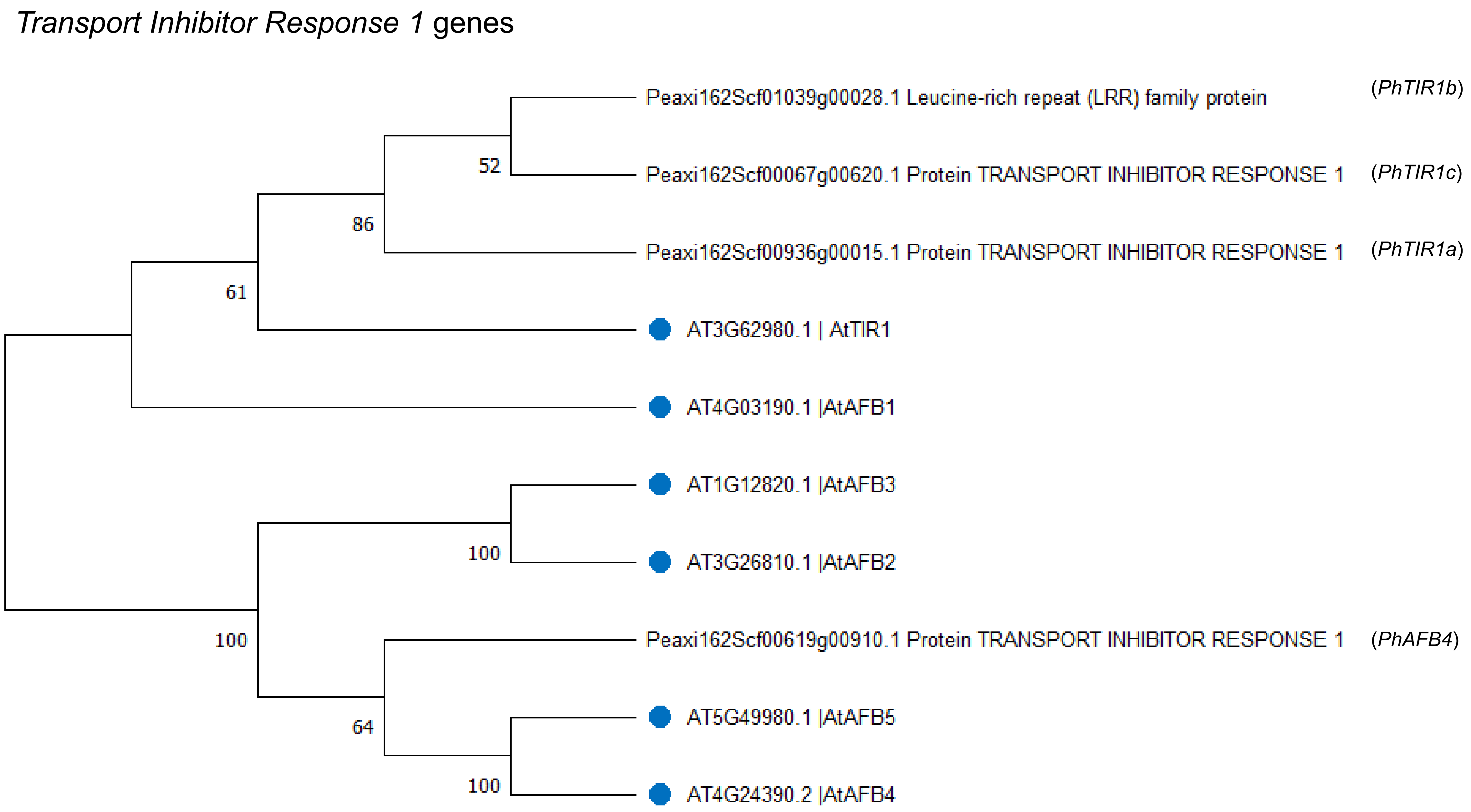
Transport Inhibitor Response 1 genes
Peaxi162Scf01039g00028.1 Leucine-rich repeat (LRR) family protein
(PhTIR1b)
52
Peaxi162Scf00067g00620.1 Protein TRANSPORT INHIBITOR RESPONSE 1
(PhTIR1c)
86
Peaxi162Scf00936g00015.1 Protein TRANSPORT INHIBITOR RESPONSE 1
(PhTIR1a)
61
AT3G62980.1 | AtTIR1
AT4G03190.1 |AtAFB1
AT1G12820.1 |AtAFB3
100
AT3G26810.1 |AtAFB2
100
Peaxi162Scf00619g00910.1 Protein TRANSPORT INHIBITOR RESPONSE 1
(PhAFB4)
64
AT5G49980.1 |AtAFB5
100
AT4G24390.2 |AtAFB4

Aux/IAA Genes

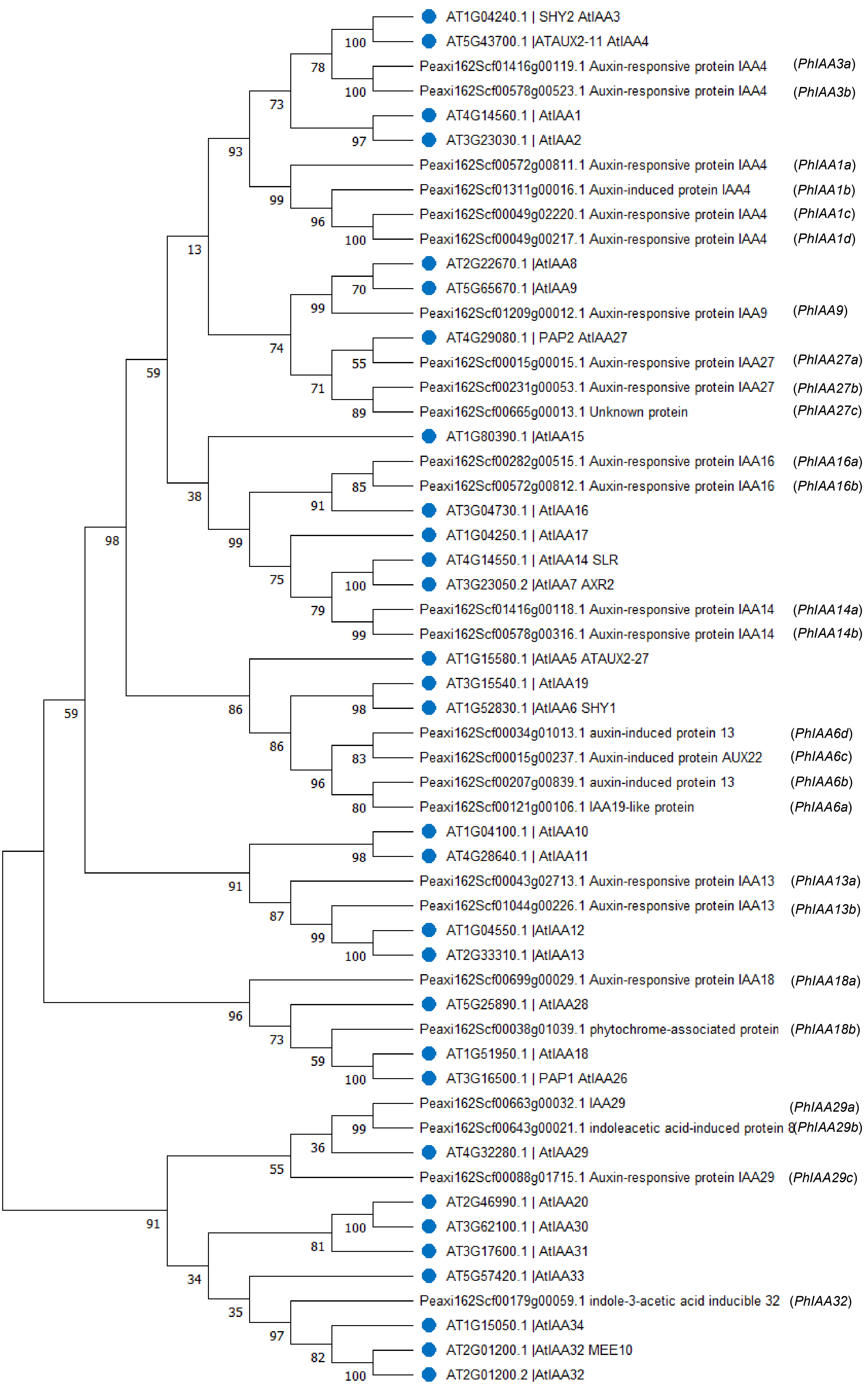

Auxin Response Factor Genes

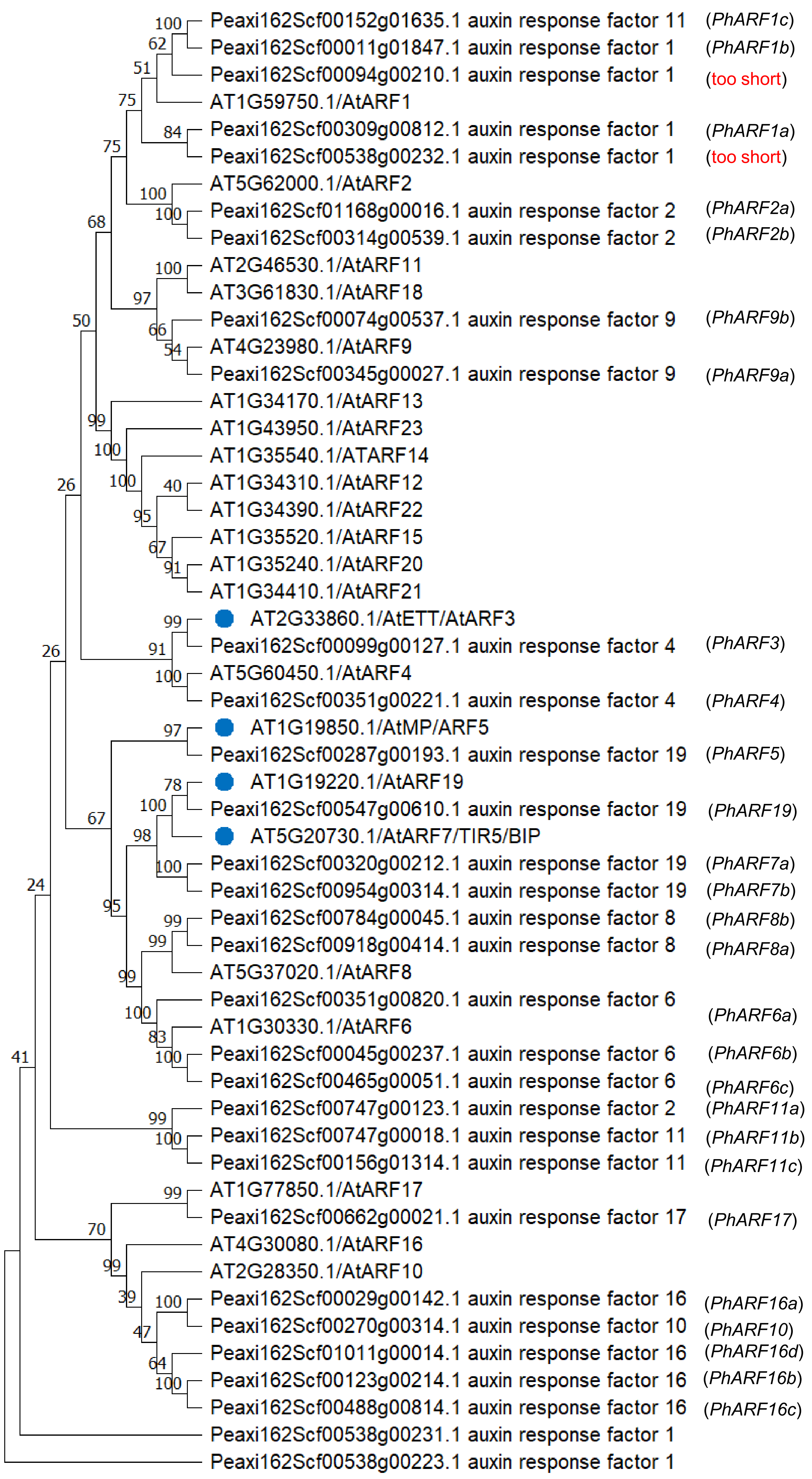

Gretchen Hagen 3 Genes

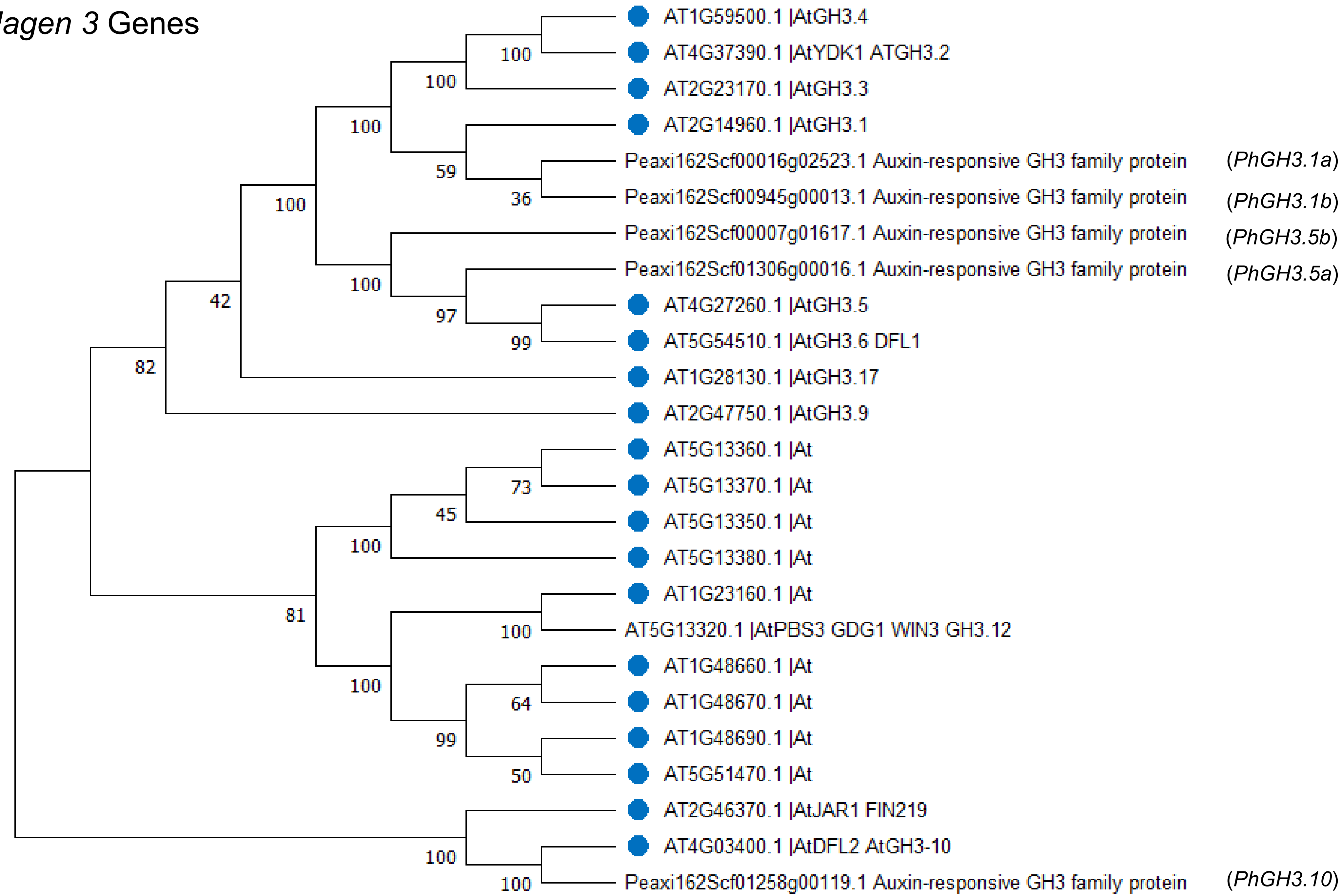

Supplement: Supplementary file 1 [file DataSheet1.pdf]
